# MCOF: A Multi-Channel Attention-Based Omics Fusion Framework for Cancer Subtyping and Candidate Biomarker Prioritization

**DOI:** 10.3390/ijms27198519

**Published:** 2026-09-24

**Authors:** Xuezhi Liang, Qi Wang, Kun Xie, Jianghui Zhang, Shiyu Feng, Xinyuan Zhong, Tingting Guo, Yi Zhang, Wen Chen

**Affiliations:** 1 Department of Medical Statistics, School of Public Health, Sun Yat-sen University, Guangzhou 510080, China; u3609265@connect.hku.hk (X.L.); wangq633@mail2.sysu.edu.cn (Q.W.);; 2 Center for Migrant Health Policy, Sun Yat-sen University, Guangzhou 510080, China

**Keywords:** multi-omics integration, cancer molecular subtyping, channel attention, integrated gradients, biomarker prioritization, deep learning

## Abstract

Multi-omics molecular subtyping is complicated by small cohorts, heterogeneous data layers, and sensitivity to data splitting and feature selection. We developed multi-channel attention-based omics fusion (MCOF), an end-to-end framework that combines omics-specific projection, one-dimensional convolution, and integrated gradients for candidate biomarker prioritization. MCOF was evaluated against six baselines using five repeated stratified holdouts across four multi-omics datasets. In the restricted four-class breast invasive carcinoma task, MCOF achieved the highest observed mean for all five metrics, including an accuracy of 0.900 ± 0.022 and a macro-averaged area under the receiver operating characteristic curve (macro-AUC) of 0.982 ± 0.003. In the restricted three-class stomach adenocarcinoma task, it achieved the highest observed macro-F1 (0.786 ± 0.035), macro-AUC (0.934 ± 0.020), and weighted-AUC (0.930 ± 0.016), whereas support vector machines had a slightly higher accuracy and weighted F1. It also achieved the highest or near-highest AUC on the two non-cancer datasets. Compared with MOGONET, MCOF used 30.6% fewer trainable parameters on both cancer tasks. Sensitivity analyses indicated that the contribution of multi-omics fusion varied by dataset and metric. Excluding the 28 prediction analysis of microarray 50 (PAM50) genes present in the retained BRCA RNA panel produced only a modest reduction in the macro-AUC, from 0.982 ± 0.003 to 0.978 ± 0.004. Implementation tests across 20 trained checkpoints detected no batch-order effect or cross-sample gradient, while the IG rankings were moderately stable across repeated holdouts and numerically consistent across class-weighting schemes and 25–100 integration steps. MCOF provides a practical framework for multi-omics classification and candidate prioritization.

## 1. Introduction

Molecularly defined disease subtypes, particularly in cancer, are rarely determined by a single layer of biology. Genomic, transcriptomic, epigenomic, proteomic, and metabolomic measurements capture complementary processes that shape tumor identity, progression, and treatment sensitivity. Integrating these layers can therefore improve molecular stratification and reveal signals that remain weak or invisible in any single modality. The practical challenge is that multi-omics cohorts are usually small relative to their dimensionality, while modalities differ markedly in their scale, noise, redundancy, and biological meaning [1,2].

Statistical integration methods such as a data integration analysis for biomarker discovery using latent components (DIABLO) provide sparse latent components and remain valuable when a compact, interpretable signature is the primary goal [3]. Conventional machine-learning classifiers can also perform well after feature concatenation. Their performance, however, is often dependent on datasets because concatenation treats modalities as exchangeable feature blocks and leaves cross-omics weighting to the downstream classifier. More broadly, benchmark studies have shown that apparent gains from multi-omics models can be dependent on the cohort size, feature selection, noise, and evaluation design, making repeated and transparent testing essential when performance differences are modest [4,5].

Deep learning approaches can learn task representations jointly with the prediction objective. Autoencoder-based methods are useful for unsupervised representation learning, whereas end-to-end networks and graph-based models can optimize directly for classification [6,7,8]. Graph models explicitly encode the sample similarity, but graph construction and multi-view fusion introduce additional modeling choices and computational costs. A more compact alternative is to project each omics layer into a shared latent space, learn modality weights, and then refine the fused representation with a lightweight classifier.

Channel attention offers a natural solution because each omics layer can be treated as a distinct information channel. The squeeze and excitation mechanisms learn channel weights from the data and can recalibrate modality contributions before downstream feature extraction [9]. When combined with one-dimensional convolution, this design permits modality-aware fusion followed by progressive representation learning without first constructing a patient graph. The question is not about whether either component is universally superior in isolation, but whether their combination provides a favorable and reproducible balance across datasets with different sample sizes, class structures, and omics compositions.

Interpretability creates a second challenge. A single high-ranking feature list can appear biologically persuasive while being unstable across data splits. Integrated gradients (IGs) provide model-aware attributions relative to a defined baseline and satisfy useful axioms for neural-network explanations [10]. For candidate biomarker prioritization, the attribution magnitude should be accompanied by evidence of repeatability. Whole-ranking correlations, selection frequencies, the top-k overlap, and the chance-adjusted overlap provide complementary views of stability [11,12].

We present multi-channel attention-based omics fusion (MCOF) for multi-omics sample classification and candidate biomarker prioritization. We evaluated MCOF using five prespecified stratified 80:20 holdouts across two cancer subtype tasks and two binary disease classification tasks. The model was compared with a statistical multi-omics method, four conventional machine-learning classifiers, and a graph-based deep-learning model; assessed through strict architectural ablation; and benchmarked for inference efficiency using trained checkpoints. To examine the robustness and potential sources of bias, we additionally evaluated models using only one omics layer and, where applicable, models excluding one layer at a time. We also assessed the sensitivity to the number of features selected per omics layer using training data only; repeated the breast cancer analysis after excluding the 28 genes from the prediction analysis of microarray 50 (PAM50) subtype classifier that were present in the retained RNA panel; reported the class-specific performance; verified that the predictions were independent of the batch composition and sample order; and evaluated the robustness of the feature rankings derived from integrated gradients (IGs) to the class aggregation and the number of numerical integration steps.

## 2. Results

### 2.1. Performance Across Cancer and Cross-Disease Classification Tasks

Table 1 summarizes the performance of MCOF and six comparators across five repeated stratified holdouts; repeat-level results are provided in Supplementary Table S1. The clearest advantage of MCOF was observed in the cancer subtype tasks. Across the breast invasive carcinoma (BRCA) and stomach adenocarcinoma (STAD) datasets, MCOF exceeded MOGONET on all ten reported metrics, while the two non-cancer datasets revealed more heterogeneous trade-offs.

For BRCA, MCOF achieved the highest mean for every reported metric: an accuracy of 0.900 ± 0.022, a macro-F1 of 0.877 ± 0.033, a weighted F1 of 0.899 ± 0.021, a macro-area under the ROC curve (macro-AUC) of 0.982 ± 0.003, and a weighted AUC of 0.979 ± 0.004. The corresponding mean advantages over the strongest conventional comparators were small—approximately 0.010–0.014 for the hard-label metrics and 0.003–0.004 for the AUC metrics, but they constantly favored MCOF at the level of the observed means.

For STAD, MCOF obtained the highest macro-F1 of 0.786 ± 0.035, macro-AUC of 0.934 ± 0.020, and weighted AUC of 0.930 ± 0.016. SVM achieved a higher accuracy (0.818 ± 0.045 versus 0.805 ± 0.030 for MCOF) and weighted F1 (0.812 ± 0.053 versus 0.806 ± 0.028).

On the Religious Orders Study and Memory and Aging Project (ROSMAP) dataset, MOGONET achieved the highest mean accuracy, F1, and Matthews correlation coefficient (MCC), whereas SVM had the highest mean AUC. MCOF reached an AUC of 0.836 ± 0.032, close to SVM (0.838 ± 0.015), but with lower hard-label metrics. On the schizophrenia (SCZ) dataset, SVM had the highest mean accuracy, F1, and MCC, whereas MCOF achieved the highest mean AUC (0.944 ± 0.040). Across all four datasets, MCOF ranked first on nine of the 18 metrics and exceeded MOGONET on 15 of the 18 shared metrics, with its strongest and most consistent advantage concentrated in BRCA and STAD. Exploratory paired analyses were consistent with the descriptive performance patterns, but had a limited inferential resolution because only five matched repetitions were available. The complete results are provided in Supplementary Data S2; accordingly, the comparisons were interpreted primarily in terms of the observed effect sizes, uncertainty, and metric-specific patterns.

The mean one-versus-rest receiver operating characteristic (ROC) curves further characterized subtype discrimination (Figure 1). For BRCA, the class-specific AUCs ranged from 0.958 ± 0.008 for luminal B to 1.000 ± 0.000 for basal-like; the HER2-enriched and luminal A subtypes achieved AUCs of 0.991 ± 0.005 and 0.978 ± 0.004, respectively. For STAD, the microsatellite instability (MSI) showed the highest AUC (0.975 ± 0.016), followed by chromosomal instability (CIN; 0.925 ± 0.022) and genomic stability (GS; 0.904 ± 0.053). These values showed strong within-cohort label recovery, but they do not constitute independent biological validation because the labels themselves are molecularly defined.

### 2.2. Sensitivity to Omics Composition, PAM50 Overlap, and Feature Budget

Single-omics and leave-one-omics-out analyses showed that the contribution of fusion depended on the dataset and metric (Supplementary Table S8). In BRCA, the RNA-only model had a slightly higher mean macro-F1 (0.885 ± 0.027) and macro-AUC (0.983 ± 0.001) than the full model (0.877 ± 0.033 and 0.982 ± 0.003), whereas the miRNA-only model was substantially weaker (macro-F1, 0.757 ± 0.034). In STAD, RNA alone had a higher mean macro-F1 (0.810 ± 0.060 versus 0.786 ± 0.035), but the full model had a higher macro-AUC (0.934 ± 0.020 versus 0.914 ± 0.029). These results indicate that RNA carried much of the cancer-label signal, while fusion changed the balance between the hard-label performance and the ranking discrimination.

The value of multiple modalities was clearer in the binary tasks. Removing DNA methylation from ROSMAP reduced the MCC from 0.504 ± 0.087 to 0.357 ± 0.081. In SCZ, the full model achieved an MCC of 0.655 ± 0.078, compared with 0.574 ± 0.213 for the protein alone and 0.602 ± 0.174 for the metabolite alone. The results therefore support modality complementarity in some settings, but not a universal claim that fusion outperforms the strongest individual modality.

To assess the label-feature overlap in BRCA, 28 PAM50-overlapping genes that were present in the frozen 1000-feature RNA panel were removed and the model was refitted using the same outer splits. The macro-F1 decreased from 0.877 ± 0.033 to 0.857 ± 0.026 and the macro-AUC from 0.982 ± 0.003 to 0.978 ± 0.004 (Supplementary Table S8). The modest decrease indicates that the direct inclusion of those 28 genes did not fully account for the observed discrimination. It does not, however, eliminate circularity because PAM50 labels are derived from expression, and correlated RNA features remain.

Training-set-only feature-budget analyses were performed within the fixed candidate panels (Supplementary Table S9). More compact panels did not provide a consistent benefit across datasets. STAD was the main exception: selecting 100 features per omics layer within the training data yielded a macro-F1 of 0.806 ± 0.056 and a macro-AUC of 0.952 ± 0.020. These results show that the original equal feature budgets were pragmatic fixed inputs rather than universally optimal values.

### 2.3. Class-Specific Performance

The class-specific results for MCOF are reported in Supplementary Table S10, and repeat-level confusion matrices for all seven methods are shown in Supplementary Figures S4 and S5. Basal-like BRCA had a sensitivity and one-versus-rest AUC of 1.000 ± 0.000, whereas HER2-enriched showed greater variability in the sensitivity (0.863 ± 0.135) and F1 (0.832 ± 0.086). Luminal B was also more difficult than basal-like and luminal A. In STAD, GS had a lower F1 than CIN and MSI. These patterns are important because the aggregate metrics alone would obscure the uncertainty of the smaller classes.

### 2.4. Strict Ablation Analysis

Figure 2 compares the complete MCOF with variants that remove channel attention or convolution on BRCA and STAD; the all-dataset analysis is shown in Supplementary Figure S1 and the values of repeats are shown in Supplementary Table S2. On BRCA, the three variants were close in their accuracy and F1, but the complete model achieved the highest accuracy (0.900 ± 0.022) and macro-AUC (0.982 ± 0.003). Removing channel attention reduced the macro-AUC to 0.979 ± 0.003, while removing convolution reduced the accuracy to 0.892 ± 0.011 and the weighted F1 to 0.891 ± 0.012.

The STAD results exposed complementary effects more clearly. Removing channel attention reduced the accuracy from 0.805 ± 0.030 to 0.782 ± 0.059, the macro-F1 from 0.786 ± 0.035 to 0.762 ± 0.059, and the macro-AUC from 0.934 ± 0.020 to 0.922 ± 0.031. In contrast, the variant without convolution produced a higher accuracy and higher F1 values, but a lower macro-AUC (0.930 ± 0.021). Across ROSMAP and SCZ, some ablated variants also improved hard-label metrics, whereas the complete model retained the highest SCZ AUC.

### 2.5. Computational Efficiency

Efficiency measurements from the existing checkpoints are summarized in Supplementary Table S3. On the two cancer datasets, MCOF used 890,760 trainable parameters for BRCA and 890,503 for STAD, compared with 1,283,948 and 1,283,326 for MOGONET. This corresponds to approximately 30.6% fewer parameters in both cancer tasks. The complete-test-set forward inference was 2.30-fold faster for MCOF on STAD (0.789 ± 0.005 ms versus 1.818 ± 0.006 ms).

The efficiency advantage was not universal. MCOF was also faster on ROSMAP and SCZ by 1.44-fold and 2.21-fold, respectively, whereas MOGONET was 1.47-fold faster on BRCA. The peak allocated memory was lower for MCOF only on BRCA and was lower for MOGONET on the other three datasets. These results define a practical, performance–efficiency trade-off of the dataset rather than a uniform reduction in all computational costs.

### 2.6. Candidate Prioritization, Implementation Checks, and Attribution Robustness

The feature rankings derived by the IG module were summarized across five complete repeats rather than truncated to a single run. Supplementary Table S4 reports the top 100 stability-ranked features, Supplementary Figure S2 shows the pairwise whole-ranking Spearman correlations, and Supplementary Table S5 and Supplementary Figure S3 report the raw and normalized overlap. Under prevalence-weighted aggregation, the mean Spearman correlations were 0.466 for BRCA, 0.474 for STAD, 0.142 for ROSMAP, and 0.384 for SCZ. Equal-class aggregation produced similar values (0.483, 0.469, 0.142, and 0.384, respectively), indicating that the main stability conclusions were not driven by class-frequency weighting.

Numerical checks further supported the use of 50 integration steps. Across datasets, the correlation between the rankings obtained with 50 and 100 steps was at least 0.998, and the corresponding top-100 Jaccard overlap was at least 0.935 (Supplementary Table S11). Complete repeat-level rankings and class-specific attribution summaries are provided in Supplementary Data S1.

The implemented model was also tested for unintended batch dependence. Across all 20 formal checkpoints, reversing the batch order produced a maximum logit difference of 0, and the maximum gradient of one sample’s selected logit with respect to any other batch row was 0. The maximum difference between evaluating a sample alone and within a batch was 2.193 × 10^−5^, below the prespecified tolerance of 1 × 10^−4^ (Supplementary Table S11).

In BRCA, the highest stability-ranked candidates were hsa-miR-4664, *PPP1R14C*, *VGLL1*, *CDH3*, and *PHYHD1*. hsa-miR-4664, *PPP1R14C*, and *VGLL1* appeared in the top 100 in all five repeats, while *CDH3* appeared in four. Other recurrent subtype-associated genes included *FOXC1* and *CXCL1*. In STAD, hsa-miR-181a-1 and *BCL2L10* appeared in the top 100 in four of five repeats, followed by *AC226118.1*, *RNF152*, *MAGOH2P*, *GPA33*, *CCND2*, and *MLH1* among the leading candidates.

The non-cancer datasets yielded biologically recognizable signals, but a lower overall ranking stability. ROSMAP repeatedly prioritized hsa-miR-132, with hsa-miR-129-5p appearing in the top 100 and top 200 in all five repeats; hsa-miR-29a was also recurrent. The highest-ranked SCZ features included P27169 (PON1), Q15166 (PON3), P49747 (COMP), P24593 (IGFBP5), and P01344 (IGF2). PON1 appeared in the top 100 in all five repeats.

DIABLO selected sparse, component feature sets across the same external repeats (Supplementary Table S6). These results provide a complementary comparison, but the two methods optimize different objectives. DIABLO selects variables for latent components, whereas IG attributes predictions from the fitted nonlinear classifier [3]. Because the overlaps of the repeats were modest, particularly for the smallest top-k sets, the reported features should be interpreted as prioritized candidates rather than definitive biomarkers. Biological interpretation in this study was based on repeat stability and concordance with prior literature.

## 3. Discussion

MCOF was designed to address a practical gap in multi-omics classification. The completed benchmark shows that its value was the clearest in cancer molecular subtyping. MCOF was the best-performing method on all five BRCA metrics and on three of five STAD metrics, including the macro-averaged metrics that are most sensitive to minority subtypes. The non-cancer datasets served as useful stress tests and prevented a misleading claim of universal superiority.

The cancer results are relevant because subtype models are often evaluated not only by their overall accuracy, but also by whether they preserve class-wise discrimination in imbalanced cohorts. On BRCA, the improvement over strong SVM and RF baselines was modest in absolute terms, but consistent across the accuracy, F1, and AUC. On STAD, SVM retained a slightly higher accuracy and weighted F1, whereas MCOF achieved a higher value for the macro-F1 and both AUC measures. This pattern shows that MCOF better preserved the average subtype separation, even when the final hard label boundary did not maximize the total accuracy. Such trade-offs are more informative than a single state-of-the-art score and are directly relevant to downstream molecular stratification.

ROSMAP and SCZ support why metric reporting is necessary. MOGONET produced a stronger hard-label performance on ROSMAP, and SVM did so on SCZ, while MCOF remained competitive in the AUC and achieved the highest SCZ AUC. The ROSMAP AUC difference between SVM and MCOF was only 0.003, which should not be interpreted as a practically decisive advantage. Instead, the results show that graph-based, kernel-based, and channel-fusion models emphasize different aspects of the decision problem. MCOF therefore offers broad transferability, but not a guarantee that one threshold or metric will be optimal for every cohort.

The strict ablation analysis also argues against an overly simple architectural narrative. Channel attention clearly benefited STAD and improved the BRCA AUC, supporting the premise that modality recalibration can help when tumor subtypes draw on uneven signals across RNA and miRNA. The convolutional module improved the BRCA hard-label performance and contributed to the highest SCZ AUC, yet the no-convolution variant achieved a slightly higher STAD accuracy and F1. These mixed, but interpretable, effects indicate that the modules are complementary and dataset-dependent. They also make the complete model easier to defend than a claim that added complexity is always beneficial.

The efficiency analysis adds a second dimension to the comparison. MCOF used approximately 30.6% fewer parameters than MOGONET on BRCA and STAD and completed inference faster on STAD, ROSMAP, and SCZ. MOGONET was faster on BRCA and used less peak memory on three datasets, so MCOF is not uniformly lighter. MCOF provided favorable parameter scaling on the cancer tasks and faster forward inference on three of four datasets. This performance–efficiency balance is particularly relevant when multi-omics models must be applied repeatedly across resampled cohorts or interpretation analyses.

The IG results provide biological validity. In BRCA, recurrent candidates included *PPP1R14C*, *VGLL1*, and *FOXC1*. *PPP1R14C* has been linked to triple-negative breast cancer progression, *VGLL1* is associated with the triple-negative basal-like phenotype, and *FOXC1* is an established basal-like marker [13,14,15]. In STAD, *BCL2L10* has experimental evidence for a tumor-suppressive feature in gastric cancer cells, while *GPA33* forms part of an intestinal differentiation panel associated with a favorable prognosis; *MLH1* is also biologically coherent with microsatellite instable subtyping [16,17]. These findings indicate that MCOF repeatedly recovers signals aligned with known subtype biology.

The rankings across different diseases showed a similar concordance with the established literature. miR-132 has functional links to adult hippocampal neurogenesis and memory in Alzheimer disease models, miR-129-5p has been associated with AD pathology and cognitive decline, and miR-29a has been proposed as a cerebrospinal-fluid biomarker [18,19,20]. In SCZ, the recurrent prioritization of PON1 is consistent with reports of reduced PON1 activity in treated schizophrenia and drug-naive first-episode psychosis [21,22]. These correspondences support biological plausibility, not causal validation. At the same time, candidates such as PHYHD1 in BRCA and RNF152, AC226118.1, and MAGOH2P in STAD remain comparatively underexplored in disease functional studies.

Several limitations should guide the interpretation. First, repeated holdout testing quantifies split sensitivity, but does not replace validation in an independent external cohort. Second, the default balanced feature budget retains the same number of candidate features per omics layer; this controls the dimensional dominance, but may not be optimal for every modality. Third, MOGONET was the only deep-learning baseline, and the study did not evaluate the survival or treatment–response outcomes. Fourth, no wet laboratory validation was performed. Finally, the original analysis-ready datasets were constructed before MCOF development by excluding 39 normal-like BRCA samples and 23 Epstein–Barr virus (EBV)-positive STAD samples. Future work should evaluate MCOF in independently assembled cohorts, include underrepresented subtypes, nest feature discovery within resampling, broaden contemporary comparisons, and experimentally validate both established and underexplored candidates.

## 4. Materials and Methods

### 4.1. Datasets and Preprocessing

Binary and multi-class classification datasets were constructed to evaluate the model performance. For the binary classification scenario, RNA, miRNA and DNA methylation data from Alzheimer’s disease (AD) patients and normal controls were retrieved from the Religious Orders Study and Memory and Aging Project (ROSMAP) [23]. In addition, proteomic and metabolite data from schizophrenia (SCZ) patients and controls were collected from another study [24]. For the multi-class classification scenario, RNA and miRNA data from breast invasive carcinoma (BRCA) and stomach adenocarcinoma (STAD) patients were collected from The Cancer Genome Atlas (TCGA) projects [25]. The labels were defined according to the PAM50 breast cancer subtypes and TCGA molecular subtypes of STAD samples [26]. The TCGA datasets were accessed through UCSC Xena (https://xenabrowser.net/datapages/; accessed on 20 May 2025), whereas the ROSMAP and SCZ datasets were obtained from the cited source studies.

The preprocessing procedures varied slightly across datasets. The ROSMAP dataset was preprocessed at acquisition and required no further handling. For the SCZ dataset, features with more than 50% missing values or zero values were removed, and only samples represented in all omics layers and in the label set were retained. The remaining features were log-transformed and Z-score-standardized, followed by a differential expression analysis for dimensionality reduction and the alignment of feature counts across modalities. For BRCA and STAD, non-frozen or adjacent-normal samples were removed, duplicate RNA entries were averaged at the gene level, and fixed 1000-feature panels were constructed for RNA and miRNA. During that upstream construction, 39 normal-like BRCA samples and 23 EBV-positive STAD samples were excluded. These exclusions preceded MCOF development and were not based on the observed model performance.

Because candidate-panel construction occurred before the five outer split assignments, the reported benchmark is a repeated stratified holdout evaluation conditional on fixed, preselected feature panels; it is not a fully nested assessment of feature discovery. After the fixed panels were defined, each dataset was divided into five prespecified stratified 80:20 training/test holdouts. Within every outer repetition, missing-value imputation and standardization used parameters estimated from the relevant training data and were applied to validation or held-out data without refitting. The held-out test labels were not used for early stopping, epoch selection, checkpoint selection, or model fitting.

For the additional feature-budget sensitivity analyses, analysis-of-variance F scores were fitted separately within each inner-training fold and, for final refitting, within the complete outer-training set. BRCA and STAD used 100, 250, or 500 selected features per modality; ROSMAP and SCZ used 50 or 100. These analyses assess the sensitivity within the fixed candidate panels and do not reconstruct the upstream high-dimensional feature-selection process. The dataset composition is summarized in Table 2.

### 4.2. Model Architecture

The overall framework of MCOF is illustrated in Figure 3a. Each omics layer is treated as a separate channel, processed by an omics-specific projector to a common latent dimension, recalibrated by a squeeze-and-excitation gate, and fused before convolutional classification. The projected representations are denoted by X′ in Figure 3. This projection step permits different raw feature dimensions across omics layers and avoids directly adding gene, microRNA, methylation, protein, and metabolite columns. All operations are performed independently for each sample; the batch axis is used only for parallel computations. The trained model is subsequently interpreted using integrated gradients (IGs), as shown in Figure 3c.

#### 4.2.1. Multi-Channel Attention Module

Assume that the multi-omics dataset is denoted as $\left\{X^{1},\;X^{2},\ldots ,{\;X}^{m},\ldots ,{\;X}^{M}\right\}$, where M is the number of omics types. Each omics matrix $X^{\;m}\;=\;\left(X_{1}^{m},\;{\;X}_{2}^{m},\;\ldots ,{\;X}_{n}^{m},\ldots ,\;X_{N}^{m}\right)’$ has dimensions N×P, with *N* samples and *P* features. The feature vector of sample n is $x_{n}^{m\;}=\;(x_{n,1}^{m},{\;x}_{n,2}^{m},\;\ldots ,{\;x}_{n,p}^{m},\ldots ,{\;x}_{n,P}^{m})$. A squeeze function $F_{sq}\left(\cdot \right)$ compresses each sample into a representative vector $P_{m}=({p_{1}}^{m},{p_{2}}^{m},\ldots ,{p_{n}}^{m},\ldots ,{p_{N}}^{m}{)}^{\prime }$ of dimensions N×M×P for each view, where the calculated ${p_{n}}^{m}$ is:

(1) $${p_{n}}^{m}=F_{sq}\left(X_{n}^{m}\right)=\frac{1}{P}\sum_{p=1}^{P}x_{n,p}^{m},m\;=1,2,\ldots M$$

The outputs are reshaped and concatenated across channels to form the multi-omics feature tensor $P_{0}$ with dimensions N×M×P×1. An excitation function $F_{ex}\left(\cdot \right)$, combining ReLU and sigmoid functions, assigns weights to each omics type:

(2) $$S\left(m\right)=F_{ex}\left(P_{m},W^{MCA}\right)=sigmoid\left[W_{\left(2\right)}^{MCA}\left(ReLU\left(W_{\left(1\right)}^{MCA}P_{m}\right)\right)\right]$$

where $W^{MCA}=(W_{\left(1\right)}^{MCA},W_{\left(2\right)}^{MCA})$ are learnable parameters. Finally, the fused multi-omics matrix is expressed as the weighted sum:

(3) $$X^{0}=\sum_{m=1}^{M}S\left(m\right)X^{m}$$

#### 4.2.2. Convolutional Classifier and Loss Module

The convolution module, constructed as a pair of feature-learning sequences, includes convolution, activation and max pooling layers configured with distinct hyperparameters. Combined with a classifier sequence comprising dropout, linear, activation and output layers, this module is deployed to extract latent representations from fused omics data and to calculate the predicted labels of samples. Inspired by AlexNet [27], we adopted one-dimensional convolution operations and a modest number of network layers to balance feature learning and the overfitting risk. A linear projector f(·) maps fused features $X^{0}$ into a latent task representation $\rho =f\left(X^{0}\right)$, which serves as the input to the convolution module. The convolutional operation is then defined as:

(4) $$pi=CNN\left(\rho ,W^{CNN}\right)=CNN\left(f\left(X^{0}\right),W^{CNN}\right)$$

where pi is the predicted probability of class k and $W^{CNN}$ are learnable parameters. The end-to-end MCOF framework is then represented as:

(5) $$pi=MCOF\left(X^{1},X^{2},\ldots ,X^{M},W^{MCA},W^{CNN}\right)=CNN\left(f\left(\sum_{m=1}^{M}S\left(m\right)X^{m}\right),W^{CNN}\right)$$

The network is trained by minimizing cross-entropy loss:

(6) $$\begin{array}{c} L_{MCOF}\left(X^{1},X^{2},\ldots ,X^{M},Y\right)=CrossEntropy\left(pi,Y\right)\\ =CrossEntropy\left(MCOF\left(X^{1},X^{2},\ldots ,X^{m},\ldots ,X^{M}\right),Y\right) \end{array}$$

where $Y\in R^{N\times K}$ is the matrix of one-hot-encoded true labels of samples.

#### 4.2.3. Integrated Gradient-Based Interpretation Module

To enhance interpretability, we incorporated the IG method [10]. For an input feature $x^{n}$, the corresponding baseline input ${\tilde{x}}^{n}$ is defined as an all-zero tensor. The IG of feature $x^{n}$ for predicted class *k* is:

(7) $$IG\left(x^{n},k\right)=\left(x^{n}-{\tilde{x}}^{n}\right)\int_{0}^{1}\frac{\partial MCOF\left({\tilde{x}}^{n}+\alpha \left(x^{n}-{\tilde{x}}^{n}\right)\right)}{\partial x^{n}}d\alpha$$

In practice, the integral $IG(x^{n},k)$ is approximated by discrete steps using interpolating points, where $x_{l}^{n}={\tilde{x}}^{n}+\alpha_{i}(x^{n}-{\tilde{x}}^{n})$, $\alpha_{i}\sim U[0,1]$. Thus,

(8) $$IG\left(x^{n},k\right)=\frac{\left(x^{n}-{\tilde{x}}^{n}\right)}{L}\sum_{l=1}^{L}\frac{\partial MCOF\left(x_{l}^{n}\right)}{\partial x^{n}}$$

For biomarker discovery, the absolute average of the IG values across all samples and categories is computed. This final feature importance score is:

(9) $$score\left(x\right)=\frac{1}{NK}\sum_{\begin{array}{c} n=1,\ldots ,N\\ k=1,\ldots ,K \end{array}}w\left|IG\left(x^{n},k\right)\right|$$

where w denotes the category weight proportional to the sample size. Features with higher scores are considered candidate biomarkers for downstream biological interpretation.

### 4.3. Repeated-Holdout Training and Model Selection

For each of the five outer repetitions, 80% of the samples formed the outer-training set and 20% formed a held-out test set. Five-fold stratified cross-validation was conducted only within the outer-training set. In every inner fold, preprocessing parameters were fitted on the inner-training portion and then applied to the validation portion. The model was re-initialized for every fold.

Early stopping monitored macro-F1 for the multiclass BRCA and STAD tasks and MCC for the binary ROSMAP and SCZ tasks, with a patience of 30 epochs. The epoch that gave the best validation metric was recorded for each fold, and the median of the five selected epochs determined the final training duration. A new model was then initialized and fitted on the complete outer-training set for that fixed number of epochs. The held-out test set was evaluated once. The resulting repeat-level checkpoint was used for efficiency measurements, class-specific evaluations, implementation tests, and attribution analyses. The full training and validation settings are listed in Supplementary Table S7.

### 4.4. Comparator, Ablation, and Sensitivity Analyses

Six comparator methods were evaluated using the same five outer split assignments. Conventional classifiers used concatenated omics features: k-nearest neighbors (KNN; 10 neighbors), support vector machine (SVM; radial-basis-function kernel, C = 1, gamma = ‘scale’, and probability estimation), Gaussian naïve Bayes (NB), and random forest (RF; 100 trees and repeat-specific random seeds). DIABLO retained separate omics blocks, used an off-diagonal design value of 0.1, and tuned keepX within the training data by five-fold cross-validation. MOGONET retained view-specific representations and used 500 pretraining epochs, 2500 joint-training epochs, and learning rates of 0.001, 0.0005, and 0.001 for pretraining, encoder updating, and the view-correlation discovery network, respectively. These settings and their tuning scope are summarized in Supplementary Table S7.

Two strict architectural variants removed the MCA module or the convolutional module. Additional sensitivity analyses fitted single-omics models for every available modality and leave-one-omics-out models for ROSMAP. The BRCA PAM50 sensitivity analysis removed the 28 PAM50 genes actually present in the frozen RNA panel. All sensitivity analyses used the same outer split assignments and training protocol as the full model (Supplementary Tables S8 and S9).

### 4.5. Evaluation Metrics and Experimental Setup

The model performance was evaluated in terms of the classification accuracy and discrimination. The accuracy was reported across all tasks. For binary classification, the additional metrics were the F1 score, AUC, and MCC [28]. For multi-class classification, the macro-F1 and weighted F1 were calculated together with the macro- and class-weighted AUCs. The results are reported as the mean ± sample standard deviation across five repeated stratified holdouts. Exploratory comparisons between MCOF and each comparator used exact two-sided sign-flip tests on the five matched repeat-level differences. The model was implemented in Python 3.9, DIABLO analyses were performed in R 4.4, and GPU experiments used NVIDIA A800 80GB PCIe devices (NVIDIA Corporation, Santa Clara, CA, USA).

## 5. Conclusions

MCOF integrates multi-channel attention, convolutional representation learning, and integrated gradients in a single framework for multi-omics classification and candidate biomarker prioritization. The model showed its strongest advantage in BRCA and STAD molecular subtyping, remained competitive on binary tasks, and offered a favorable inference speed on three datasets. The performance remained high after removing 28 PAM50-overlapping genes, while implementation tests confirmed the absence of cross-sample information flow and the attribution rankings were robust to class aggregation and integration-step choice. These results support MCOF as a practical and interpretable framework for multi-omics molecular stratification, while independent cohort validation and functional follow-up remain essential.

## Figures and Tables

**Figure 1:**
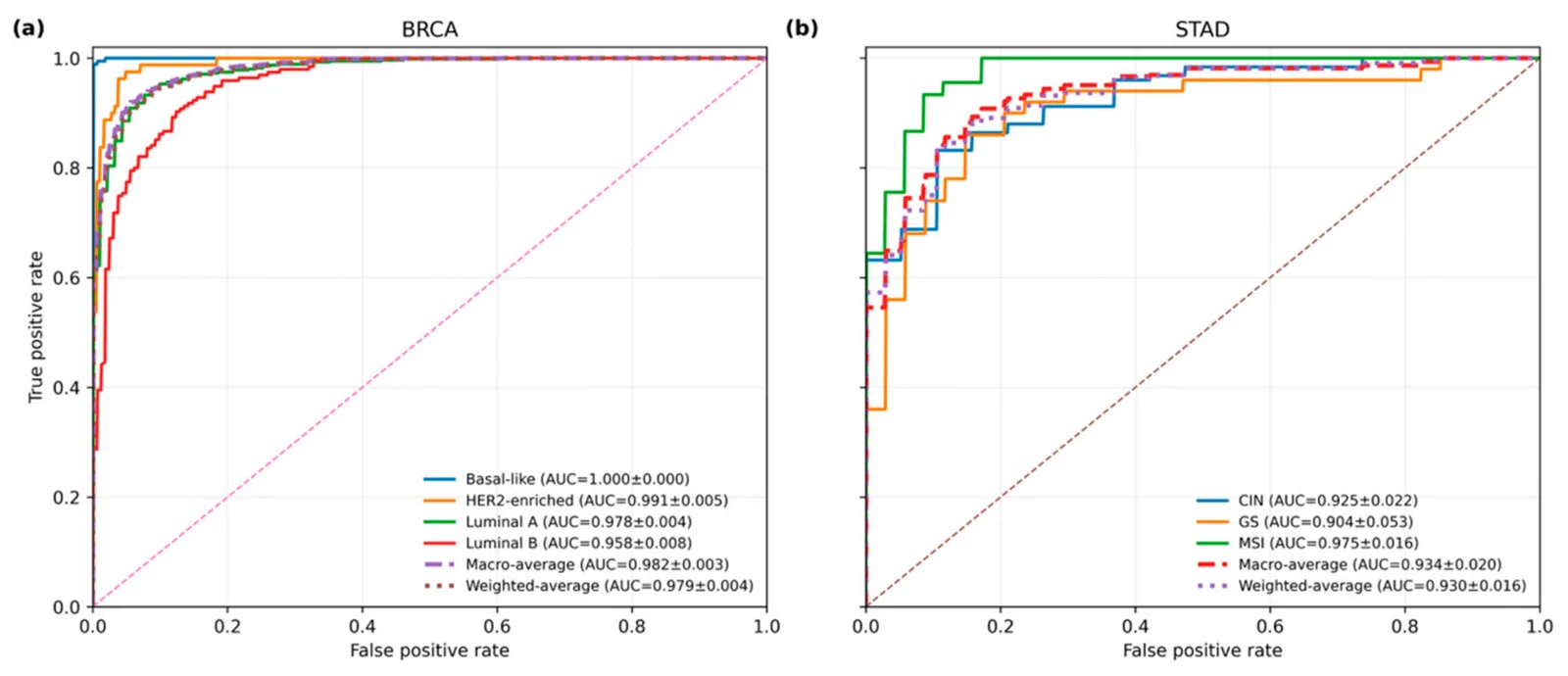
Mean one-versus-rest ROC curves of MCOF for cancer subtype classification tasks across five repeated stratified holdouts. (**a**) BRCA classification involving the basal-like, HER2-enriched, luminal A, and luminal B subtypes. (**b**) STAD classification involving the chromosomal instability (CIN), genomic stability (GS), and microsatellite instability (MSI) subtypes. Solid lines represent the mean class-specific curves, and macro-averaged and class-frequency-weighted ROC curves are also shown. AUC values in the legends are reported as the mean ± SD across the five repeated stratified holdouts. The diagonal reference line denotes chance-level discrimination. BRCA, breast invasive carcinoma; STAD, stomach adenocarcinoma; AUC, area under the receiver operating characteristic curve.

**Figure 2:**
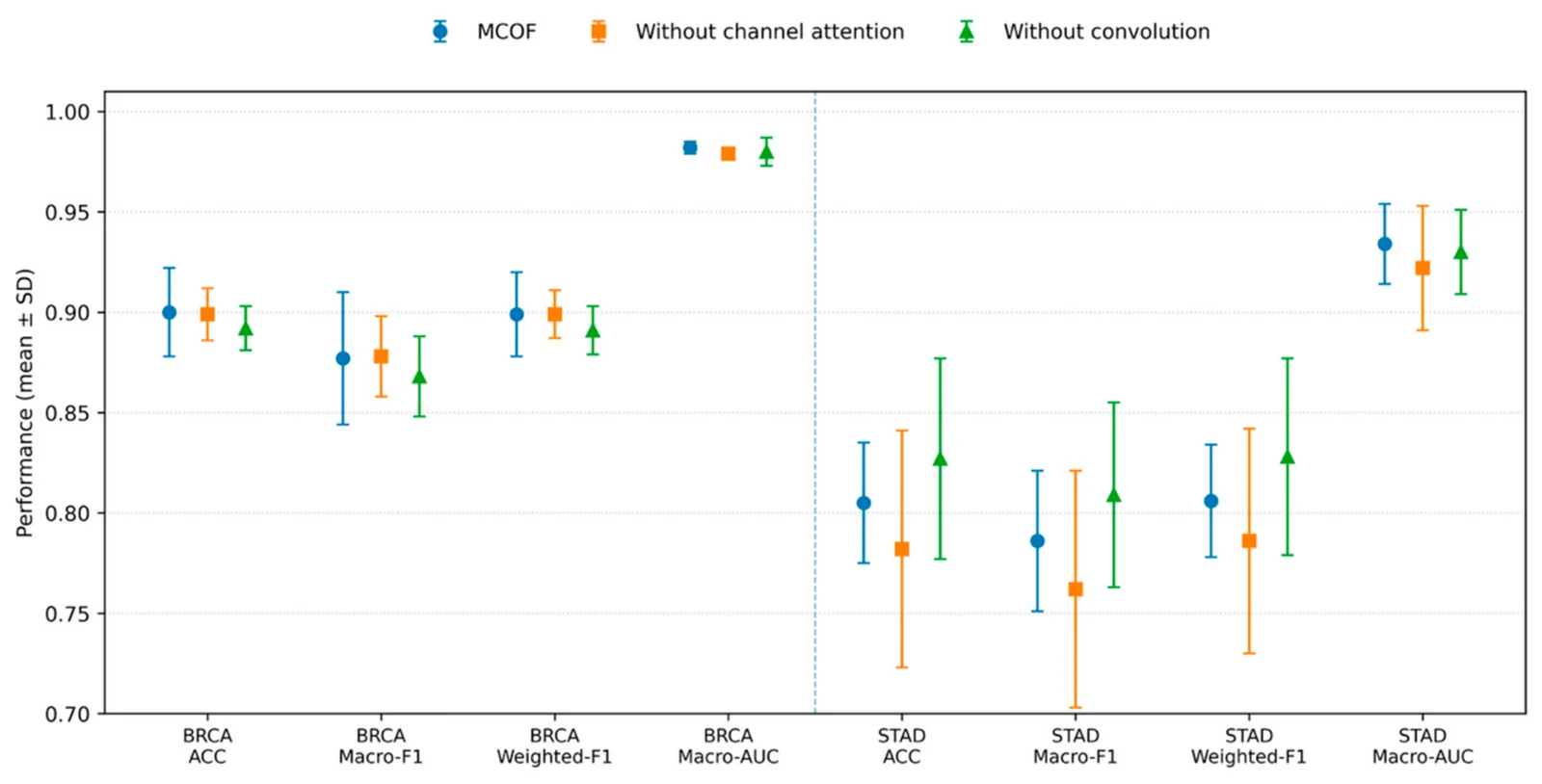
Strict ablation analysis of MCOF in cancer subtype classification. The complete model was compared with variants without channel attention and without convolution on BRCA and STAD. Points and error bars represent mean ± SD across five repeated stratified holdouts. The corresponding all-dataset analysis is provided in Supplementary Figure S1.

**Figure 3:**
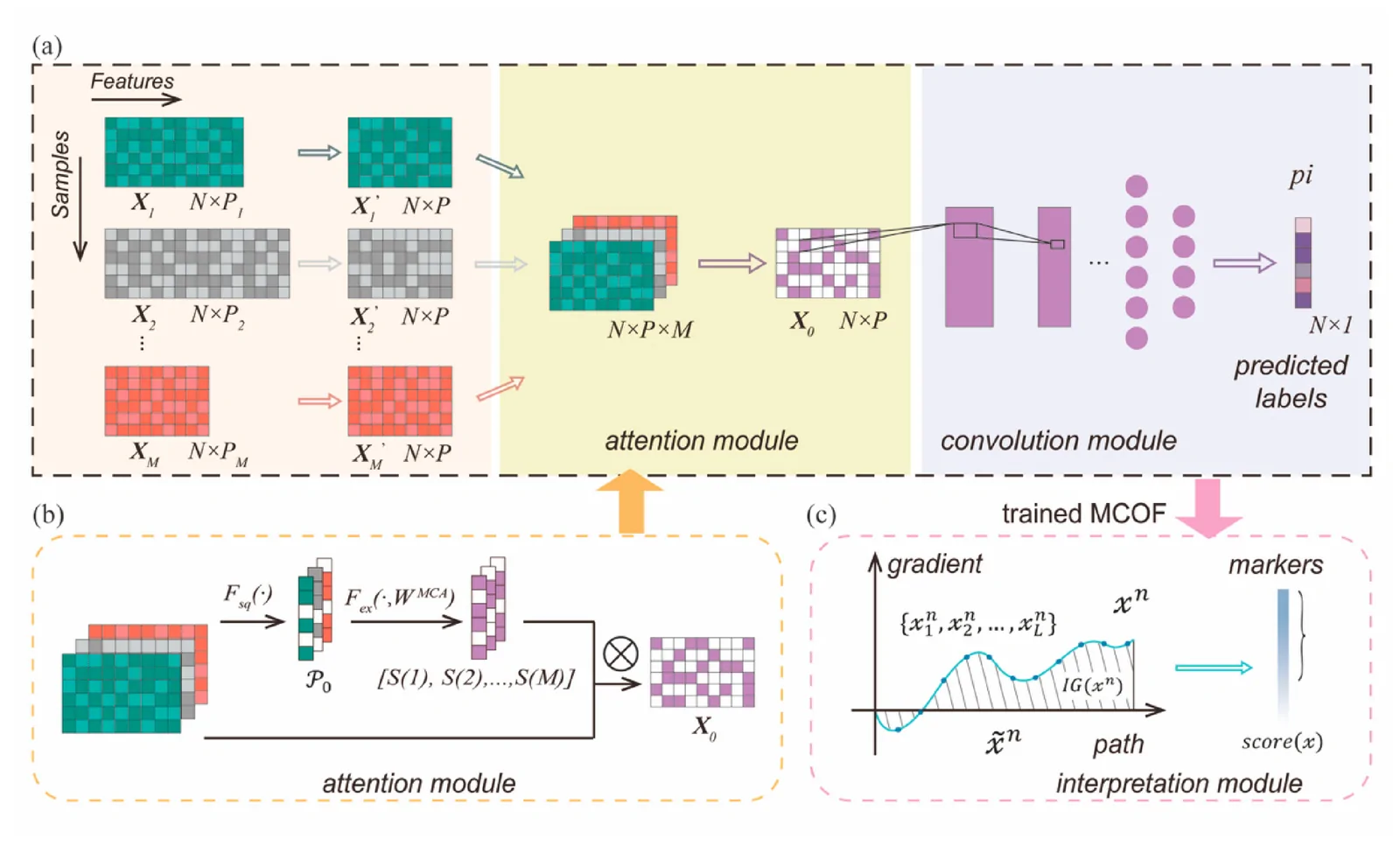
Framework of the multi-channel attention-based omics fusion (MCOF) model. (**a**) Overall architecture of MCOF. The differently colored input matrices represent distinct omics layers. Each omics layer $X_{m}$, with N samples and $P_{m}$ raw features, is independently projected to a common latent dimension, shown schematically as $X_{m}^{\prime }$ with width P. The projected omics representations are stacked across M omics layers and passed to the channel-attention module, which assigns sample-specific weights to the omics representations and produces the fused representation $X_{0}$. The fused latent representation is then processed by the one-dimensional convolutional classifier to generate class predictions. The rightmost N×1 element schematically denotes the predicted class label for each sample; internally, the classifier produces class logits and probabilities. Ellipses denote repeated omics layers, features, or network units and do not represent missing data or results. (**b**) Multi-channel attention module. The squeeze operation, $F_{sq}$, summarizes each projected omics representation, and the excitation operation, $F_{ex}$, transforms these summaries into sample-specific channel weights, denoted schematically by S(m). The circled multiplication symbol indicates channel reweighting; the weighted representations are then summed to obtain $X_{0}$. (**c**) Integrated gradient-based interpretation module. The curve and shaded area schematically illustrate gradient integration along a path from a baseline to the observed input. The resulting feature-attribution scores are used for candidate biomarker prioritization. Colored matrices distinguish omics layers, while purple blocks and circles represent schematic feature maps and network units.

**Table 1:** Classification performance of MCOF and baseline models across cancer subtyping and cross-disease tasks.

Metric	DIABLO	KNN	SVM	NB	RF	MOGONET	MCOF
**BRCA**							
ACC	0.822 ± 0.016	0.777 ± 0.004	0.890 ± 0.010	0.753 ± 0.022	0.885 ± 0.008	0.858 ± 0.025	**0.900 ± 0.022**
Macro-F1	0.817 ± 0.013	0.652 ± 0.005	0.863 ± 0.015	0.702 ± 0.028	0.859 ± 0.011	0.800 ± 0.053	**0.877 ± 0.033**
Weighted F1	0.830 ± 0.015	0.710 ± 0.007	0.887 ± 0.010	0.764 ± 0.020	0.880 ± 0.008	0.850 ± 0.029	**0.899 ± 0.021**
Macro-AUC	0.952 ± 0.004	0.911 ± 0.017	0.978 ± 0.004	0.861 ± 0.009	0.977 ± 0.005	0.937 ± 0.025	**0.982 ± 0.003**
Weighted AUC	0.949 ± 0.005	0.914 ± 0.015	0.976 ± 0.005	0.886 ± 0.011	0.974 ± 0.005	0.941 ± 0.022	**0.979 ± 0.004**
**STAD**							
ACC	0.745 ± 0.084	0.673 ± 0.061	**0.818 ± 0.045**	0.450 ± 0.073	0.795 ± 0.058	0.732 ± 0.044	0.805 ± 0.030
Macro-F1	0.712 ± 0.086	0.548 ± 0.079	0.779 ± 0.066	0.363 ± 0.088	0.742 ± 0.086	0.667 ± 0.075	**0.786 ± 0.035**
Weighted F1	0.757 ± 0.076	0.631 ± 0.065	**0.812 ± 0.053**	0.446 ± 0.075	0.778 ± 0.071	0.715 ± 0.058	0.806 ± 0.028
Macro-AUC	0.890 ± 0.037	0.831 ± 0.014	0.921 ± 0.030	0.528 ± 0.049	0.921 ± 0.034	0.801 ± 0.064	**0.934 ± 0.020**
Weighted AUC	0.907 ± 0.034	0.834 ± 0.016	0.922 ± 0.032	0.530 ± 0.052	0.922 ± 0.034	0.795 ± 0.063	**0.930 ± 0.016**
**ROSMAP**							
ACC	0.693 ± 0.035	0.707 ± 0.035	0.758 ± 0.025	0.732 ± 0.017	0.735 ± 0.023	**0.772 ± 0.035**	0.749 ± 0.040
F1	0.675 ± 0.048	0.665 ± 0.052	0.761 ± 0.026	0.726 ± 0.020	0.735 ± 0.032	**0.795 ± 0.028**	0.751 ± 0.037
AUC	0.765 ± 0.031	0.746 ± 0.045	**0.838 ± 0.015**	0.780 ± 0.015	0.802 ± 0.022	0.830 ± 0.047	0.836 ± 0.032
MCC	0.398 ± 0.067	0.445 ± 0.061	0.519 ± 0.054	0.473 ± 0.040	0.474 ± 0.043	**0.548 ± 0.069**	0.504 ± 0.087
**SCZ**							
ACC	0.705 ± 0.132	0.790 ± 0.054	**0.838 ± 0.054**	0.829 ± 0.072	0.810 ± 0.089	0.771 ± 0.040	0.819 ± 0.040
F1	0.713 ± 0.113	0.805 ± 0.055	**0.840 ± 0.055**	0.829 ± 0.083	0.805 ± 0.101	0.772 ± 0.042	0.814 ± 0.048
AUC	0.802 ± 0.140	0.887 ± 0.037	0.933 ± 0.043	0.907 ± 0.061	0.875 ± 0.105	0.882 ± 0.045	**0.944 ± 0.040**
MCC	0.414 ± 0.271	0.592 ± 0.117	**0.684 ± 0.111**	0.666 ± 0.143	0.630 ± 0.175	0.554 ± 0.077	0.655 ± 0.078

Values are the mean ± SD across five repeated stratified holdouts. Bold values indicate the highest observed mean for each dataset–metric combination. For BRCA and STAD, the macro- and class-frequency-weighted one-vs-rest AUCs are reported. ACC, accuracy; AUC, area under the receiver operating characteristic curve; MCC, Matthews correlation coefficient; DIABLO, data integration analysis for biomarker discovery using latent components; KNN, k-nearest neighbors; SVM, support vector machine; NB, naive Bayes; RF, random forest.

**Table 2:** Summary of sample composition and omics layers for the four multi-omics datasets used in this study.

**Panel A. Sample composition.**					
**Dataset**		**Sample Size (*n*)**		**Group**	**Group (*n*)**
ROSMAP		351		Normal Control	169
				Alzheimer’s Disease	182
SCZ		104		Normal Control	51
				Schizophrenia	53
BRCA		1002		Basal-Like	180
				HER2-Enriched	80
				Luminal A	547
				Luminal B	195
STAD		217		CIN	122
				GS	48
				MSI	47
**Panel B. Omics composition.**					
**Dataset**	**Omics Type**		**Feature Count**		
ROSMAP	mRNA		200		
	miRNA		200		
	DNA Methylation		200		
SCZ	Protein		200		
	Metabolite		200		
BRCA	RNA		1000		
	miRNA		1000		
STAD	RNA		1000		
	miRNA		1000		

Panel A summarizes the total sample size and class distribution for each dataset. Panel B summarizes the omics layers included in each dataset and the number of retained features per layer. ROSMAP, Religious Orders Study and Memory and Aging Project; SCZ, schizophrenia; BRCA, breast invasive carcinoma; basal-like, basal-like breast cancer; HER2-enriched, human epidermal growth factor receptor 2-enriched breast cancer; luminal A/B, luminal breast cancer subtypes; STAD, stomach adenocarcinoma; CIN, chromosomal instability; GS, genomic stability; MSI, microsatellite instability.

## Data Availability

The TCGA BRCA and STAD datasets analyzed in this study are publicly available through the UCSC Xena platform. The Religious Orders Study and Memory and Aging Project (ROSMAP) data are available through the AD Knowledge Portal/Synapse (Study ID: syn3219045), subject to the applicable data-use agreement and access approval. The schizophrenia plasma proteomics and metabolomics data are available through ProteomeXchange and Mass spectrometry Interactive Virtual Environment (MassIVE) under accession numbers PXD024474 and MSV000086975, respectively. The source code is provided as Supplementary Code for editorial and peer review. A public GitHub repository containing the source code and analysis scripts can be accessed as follows: https://github.com/xzliang-k/MCOF-multiomics.git (accessed on 16 September 2026). No new primary biological samples or participant data were collected in this study.

## Supplementary Materials

The following supporting information can be downloaded at: https://www.mdpi.com/article/10.3390/ijms27198519/s1.
