# Supplementary material for: MCOF: A Multi-Channel Attention-Based Omics Fusion Framework for Cancer Subtyping and Candidate Biomarker Prioritization
Source: Int J Mol Sci. 2026 Sep 24;27(19):8519. doi: 10.3390/ijms27198519 (PMC13664771; doi:10.3390/ijms27198519)
Supplement: Supplementary file 1 [file ijms-27-08519-s001.zip › ijms-4520828-supplementary tables and figures.pdf]

Supplementary Tables and Figures

Figure S1 Ablation study of MCOF across four multi-omics classification datasets........................2

Figure S2 Reproducibility of integrated gradients (IG)-based feature rankings across five repeated runs of MCOF 3

Figure S3 Stability of IG-selected top-ranked features across five repeated runs of MCOF 4

Figure S4 Confusion matrices of all evaluated models for the restricted four-class BRCA task 5

Figure S5 Confusion matrices of all evaluated models for the restricted three-class STAD task ...6

Table S1 The five-time performances of MCOF and baseline models on test sets of ROSMAP, SCZ, BRCA and STAD 7

Table S2 Classification performance of MCOF and its ablated variants across five independent repetitions on four multi-omics datasets...11

Table S3 Computational complexity and inference efficiency of MCOF and MOGONET across four datasets 16

Table S4 Cross-repeat stability of biomarkers identified by MCOF using integrated gradients..17

Table S5 Overall stability assessment of IG-derived biomarker rankings across five independent repeats...45

Table S6 Features selected by DIABLO across five independent repeats 46

Table S7 Training, validation, and comparator settings for MCOF and benchmark models.......72

Table S8 Sensitivity of MCOF performance to omics composition and exclusion of PAM50-overlapping genes from the BRCA RNA panel..73

Table S9 Sensitivity of MCOF performance to training-set-only feature budgets within the fixed candidate panels...75

Table S10 Class-specific held-out performance of MCOF on the restricted BRCA and STAD subtype tasks 77

Table S11 Implementation and numerical robustness checks for MCOF.......................................78

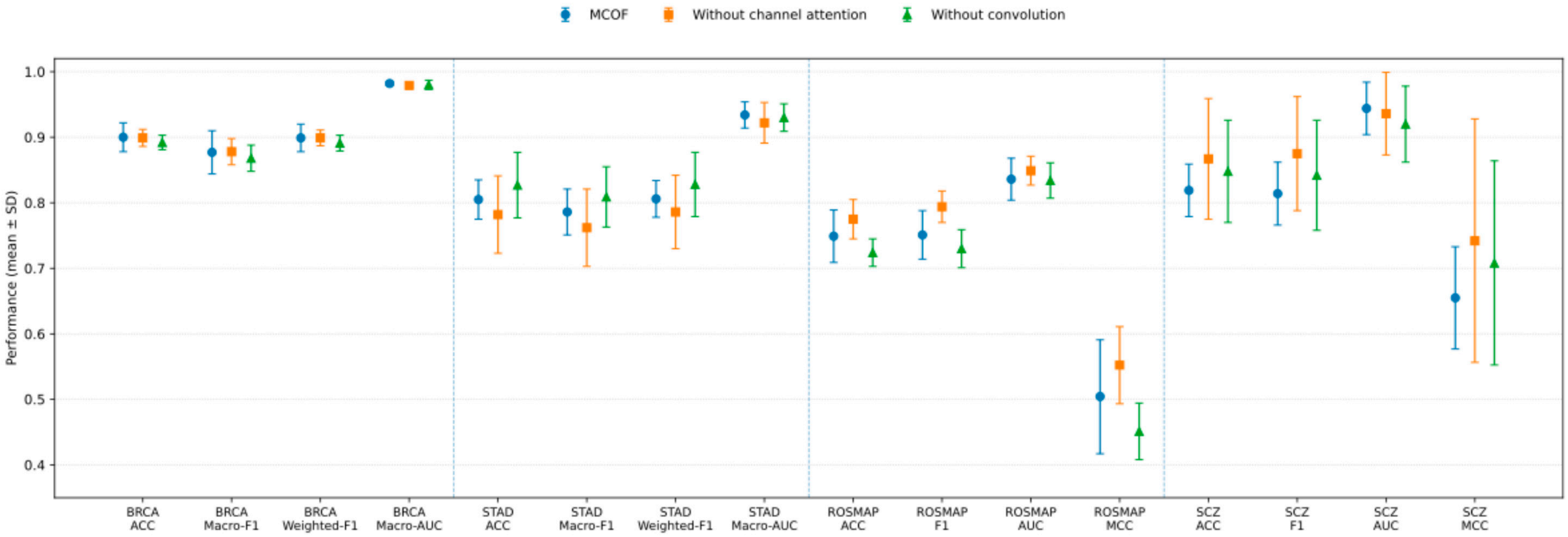

Figure S1 Ablation study of MCOF across four multi-omics classification datasets

Note: Points represent the mean test-set performance across five independent repetitions of data splitting, model training, and evaluation, and error bars indicate ±1 standard deviation. MCOF denotes the complete model we presented. "Without channel attention" denotes the ablated variant with the multi-channel attention module removed, whereas "Without convolution" denotes the variant with the convolutional feature-extraction module removed. Vertical dashed lines separate the four datasets. For the multi-class BRCA and STAD datasets, ACC, Macro-F1, Weighted-F1, and Macro-AUC are reported; for the binary ROSMAP and SCZ datasets, ACC, F1, AUC, and MCC are reported. ACC, accuracy; F1, F1 score; Macro-F1, macro-averaged F1 score; Weighted-F1, weighted-averaged F1 score; AUC, area under the receiver operating characteristic curve; Macro-AUC, macro-averaged AUC; MCC, Matthews correlation coefficient; BRCA, breast invasive carcinoma; STAD, stomach adenocarcinoma; ROSMAP, Religious Orders Study and Memory and Aging Project; SCZ, schizophrenia.

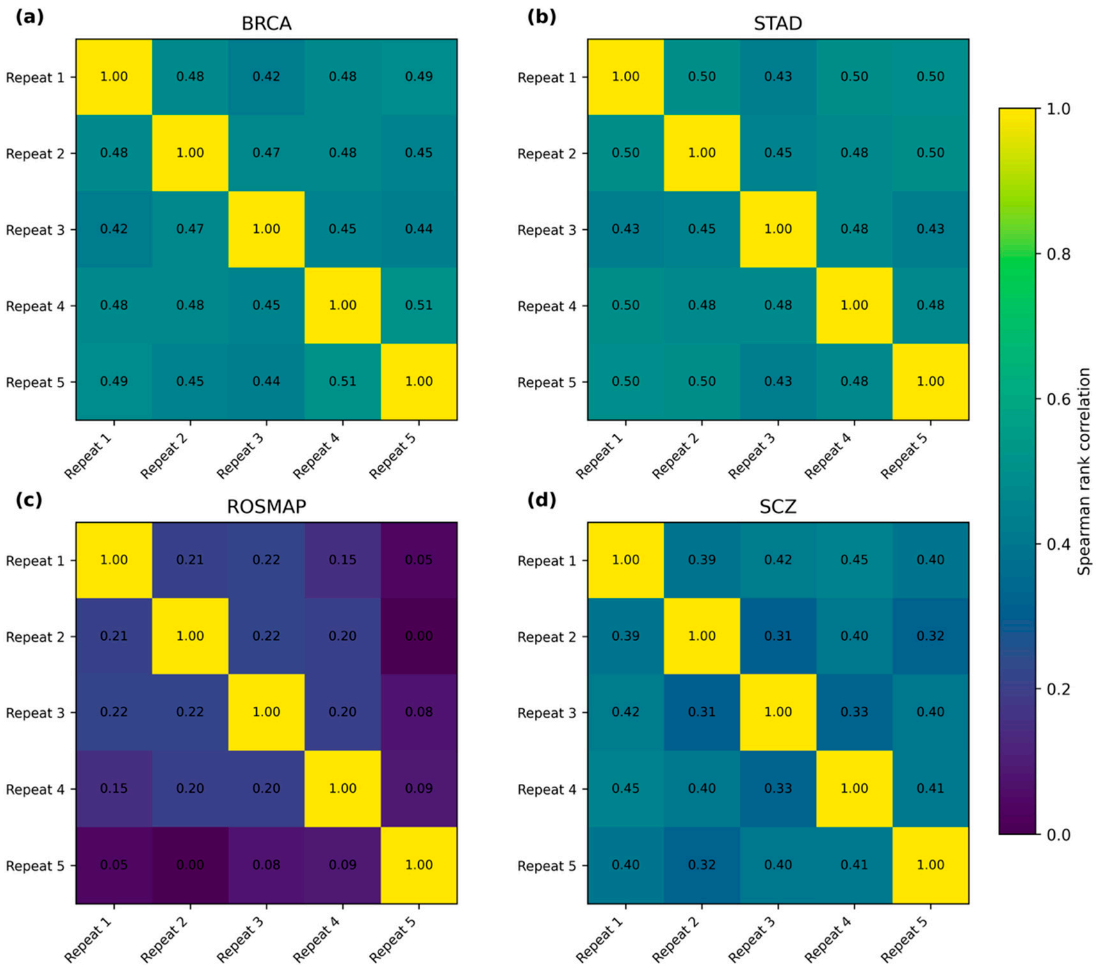

Figure S2 Reproducibility of integrated gradients (IG)-based feature rankings across five repeated runs of MCOF

Note: Heatmaps show pairwise Spearman rank correlations of IG importance scores between repeated run for each dataset: (a) BRCA, (b) STAD, (c) ROSMAP, and (d) SCZ. Each cell represents the rank correlation between two repeats, and the diagonal values are 1.00 by definition. Higher correlation indicates better reproducibility of the overall feature-importance ranking identified by MCOF. BRCA, breast invasive carcinoma; STAD, stomach adenocarcinoma; ROSMAP, Religious Orders Study and Memory and Aging Project; SCZ, schizophrenia.

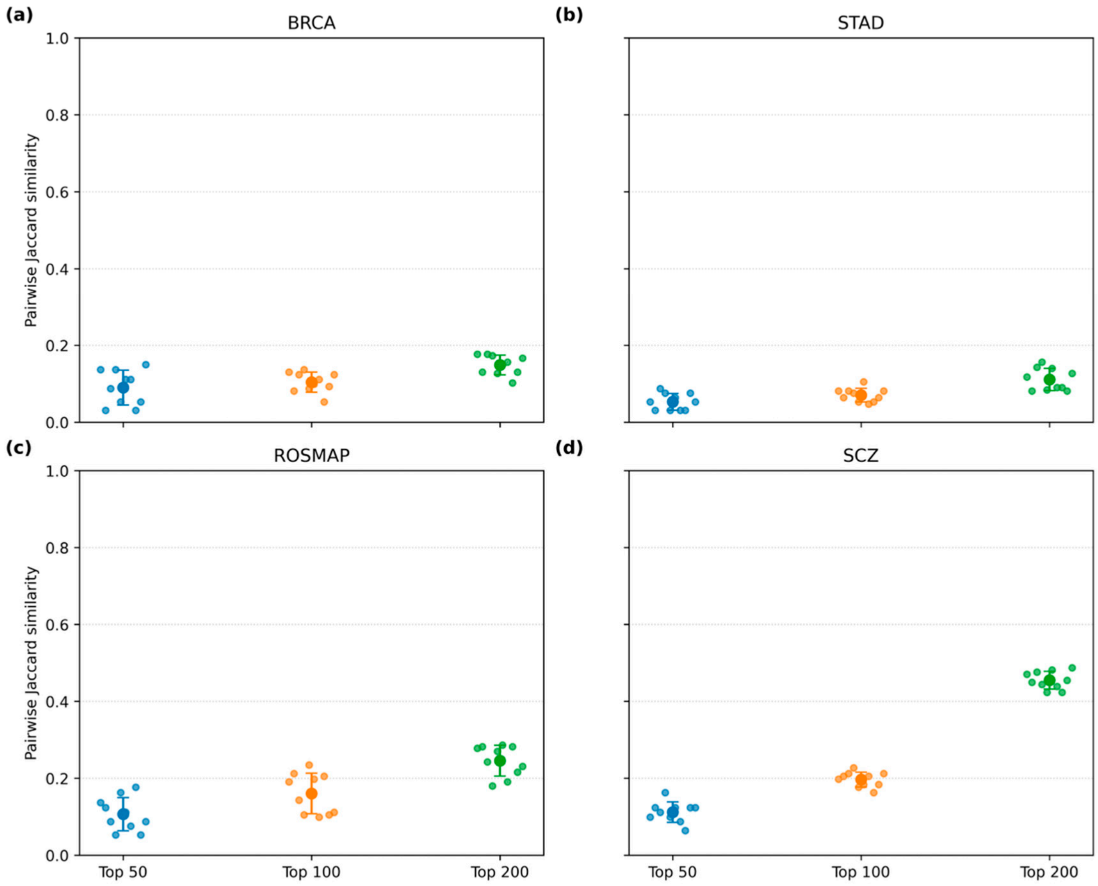

Figure S3 Stability of IG-selected top-ranked features across five repeated runs of MCOF

Note: Pairwise Jaccard similarities of the IG-selected top-ranked feature sets are shown for each dataset: (a) BRCA, (b) STAD, (c) ROSMAP, and (d) SCZ. For each dataset, the overlap stability was evaluated for the top 50, top 100, and top 200 features by comparing all pairwise combinations of repeated runs. Dots represent pairwise Jaccard similarity values, and the central error bars indicate the mean ± standard deviation. Higher Jaccard similarity indicates greater consistency of the selected biomarker set across repeated runs. BRCA, breast invasive carcinoma; STAD, stomach adenocarcinoma; ROSMAP, Religious Orders Study and Memory and Aging Project; SCZ, schizophrenia.

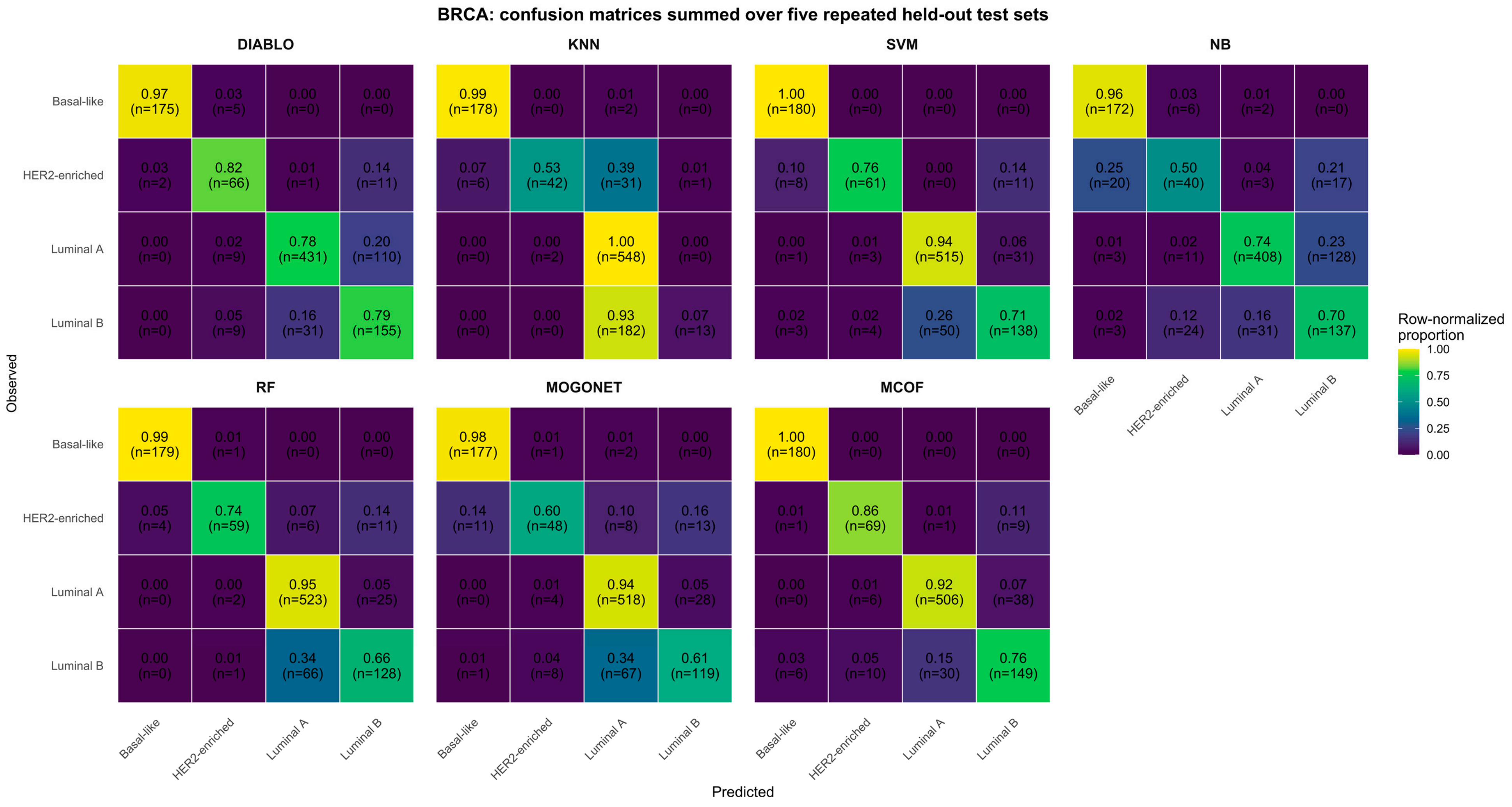

Figure S4 Confusion matrices of all evaluated models for the restricted four-class BRCA task

Note: Cells show row-normalized proportions, with summed classification counts in parentheses, across five repeated held-out test sets. Because a sample can occur in more than one repeated holdout, the summed counts are descriptive and are not treated as independent observations. The displayed values are derived from the repeat-level confusion-matrix counts supplied in Supplementary Data S2.

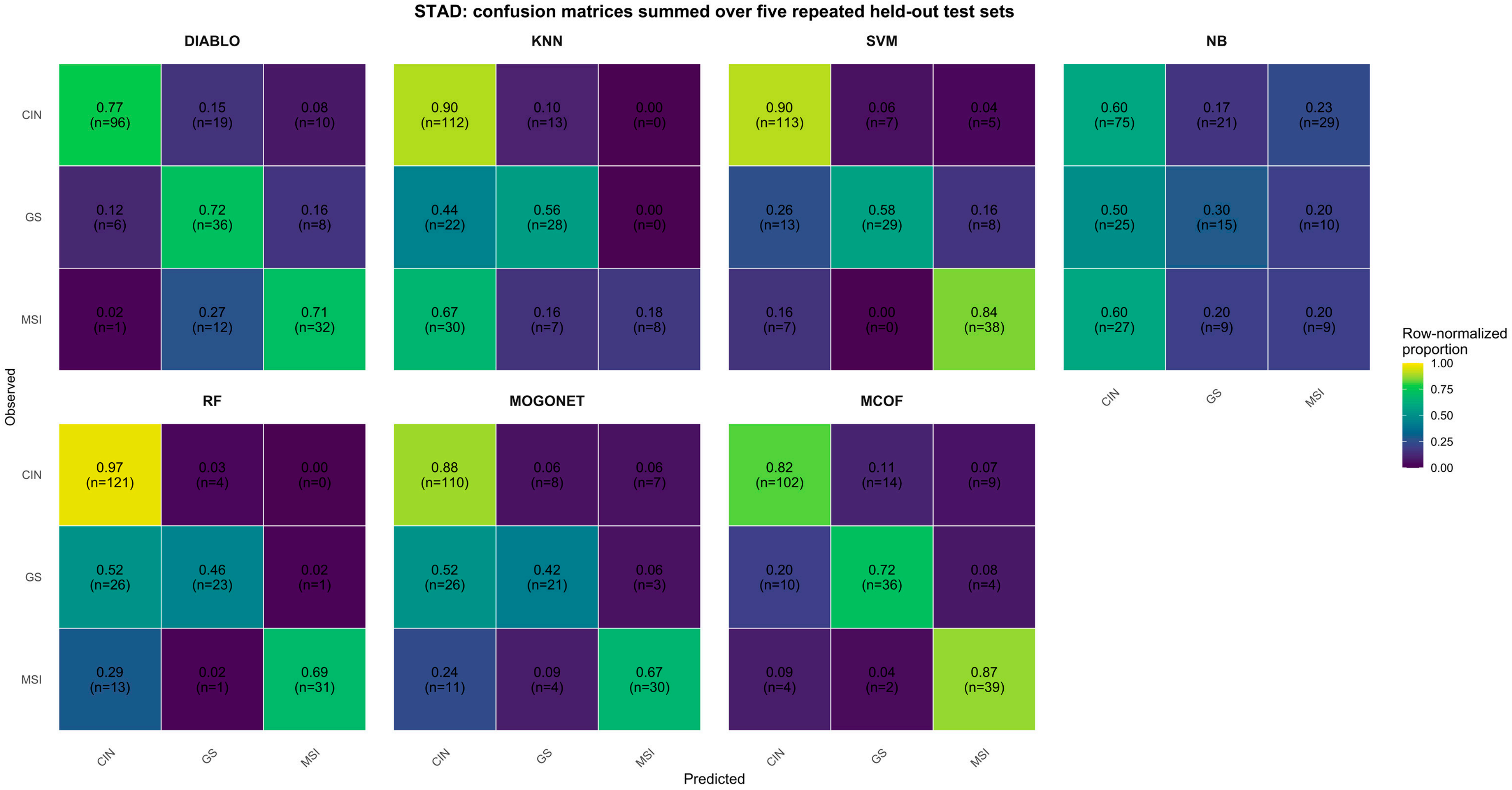

Figure S5 Confusion matrices of all evaluated models for the restricted three-class STAD task

Note: Cells show row-normalized proportions, with summed classification counts in parentheses, across five repeated held-out test sets. Because a sample can occur in more than one repeated holdout, the summed counts are descriptive and are not treated as independent observations. CIN, chromosomal instability; GS, genomically stable; MSI, microsatellite instability. Source counts are supplied in Supplementary Data S2.

Table S1 The five-time performances of MCOF and baseline models on test sets of ROSMAP, SCZ, BRCA and STAD

Models	Metrics	1	2	3	4	5	Mean	SD
**ROSMAP**								
DIABLO	ACC	0.662	0.690	0.746	0.704	0.662	0.693	0.035
	F1	0.625	0.686	0.750	0.667	0.647	0.675	0.048
	AUC	0.758	0.785	0.750	0.727	0.807	0.765	0.031
	MCC	0.344	0.385	0.494	0.436	0.332	0.398	0.067
KNN	ACC	0.704	0.704	0.761	0.662	0.704	0.707	0.035
	F1	0.656	0.656	0.746	0.600	0.667	0.665	0.052
	AUC	0.713	0.797	0.792	0.700	0.728	0.746	0.045
	MCC	0.447	0.447	0.535	0.362	0.436	0.445	0.061
SVM	ACC	0.732	0.761	0.775	0.789	0.732	0.758	0.025
	F1	0.740	0.761	0.795	0.776	0.732	0.761	0.026
	AUC	0.849	0.838	0.857	0.830	0.818	0.838	0.015
	MCC	0.465	0.524	0.550	0.592	0.467	0.519	0.054
NB	ACC	0.718	0.732	0.732	0.761	0.718	0.732	0.017
	F1	0.697	0.732	0.747	0.738	0.714	0.726	0.020
	AUC	0.799	0.794	0.768	0.771	0.770	0.780	0.015
	MCC	0.452	0.467	0.463	0.543	0.441	0.473	0.040
RF	ACC	0.704	0.761	0.746	0.718	0.746	0.735	0.023
	F1	0.704	0.767	0.757	0.697	0.750	0.735	0.032
	AUC	0.794	0.840	0.790	0.796	0.788	0.802	0.022
	MCC	0.411	0.521	0.492	0.452	0.494	0.474	0.043
MOGONET	ACC	0.746	0.746	0.761	0.831	0.775	0.772	0.035
	F1	0.780	0.769	0.779	0.838	0.810	0.795	0.028
	AUC	0.756	0.815	0.849	0.873	0.859	0.830	0.047
	MCC	0.500	0.493	0.520	0.661	0.567	0.548	0.069
MCOF	ACC	0.690	0.746	0.761	0.803	0.746	0.749	0.040
	F1	0.694	0.735	0.773	0.788	0.763	0.751	0.037
	AUC	0.828	0.849	0.867	0.850	0.784	0.836	0.032
	MCC	0.381	0.503	0.520	0.624	0.492	0.504	0.087
**SCZ**								
DIABLO	ACC	0.476	0.714	0.762	0.810	0.762	0.705	0.132
	F1	0.522	0.727	0.737	0.818	0.762	0.713	0.113
	AUC	0.555	0.864	0.827	0.873	0.891	0.802	0.140
	MCC	-0.055	0.427	0.552	0.618	0.527	0.414	0.271
KNN	ACC	0.714	0.762	0.810	0.857	0.810	0.790	0.054

Models	Metrics	1	2	3	4	5	Mean	SD
	F1	0.727	0.800	0.800	0.880	0.818	0.805	0.055
	AUC	0.827	0.905	0.877	0.918	0.909	0.887	0.037
	MCC	0.427	0.539	0.633	0.742	0.618	0.592	0.117
SVM	ACC	0.762	0.857	0.857	0.905	0.810	0.838	0.054
	F1	0.762	0.870	0.842	0.909	0.818	0.840	0.055
	AUC	0.864	0.936	0.964	0.973	0.927	0.933	0.043
	MCC	0.527	0.716	0.748	0.809	0.618	0.684	0.111
NB	ACC	0.714	0.905	0.810	0.857	0.857	0.829	0.072
	F1	0.700	0.917	0.800	0.870	0.857	0.829	0.083
	AUC	0.827	0.973	0.900	0.873	0.964	0.907	0.061
	MCC	0.440	0.823	0.633	0.716	0.718	0.666	0.143
RF	ACC	0.714	0.905	0.762	0.762	0.905	0.810	0.089
	F1	0.700	0.917	0.737	0.762	0.909	0.805	0.101
	AUC	0.705	0.932	0.895	0.864	0.982	0.875	0.105
	MCC	0.440	0.823	0.552	0.527	0.809	0.630	0.175
MOGONET	ACC	0.810	0.714	0.762	0.810	0.762	0.771	0.040
	F1	0.818	0.750	0.737	0.818	0.737	0.772	0.042
	AUC	0.873	0.836	0.945	0.909	0.845	0.882	0.045
	MCC	0.618	0.430	0.552	0.618	0.552	0.554	0.077
MCOF	ACC	0.857	0.810	0.810	0.762	0.857	0.819	0.040
	F1	0.857	0.833	0.800	0.737	0.842	0.814	0.048
	AUC	0.891	0.955	0.945	0.927	1.000	0.944	0.040
	MCC	0.718	0.626	0.633	0.552	0.748	0.655	0.078
**BRCA**								
DIABLO	ACC	0.816	0.846	0.826	0.801	0.821	0.822	0.016
	F1-macro	0.815	0.839	0.810	0.805	0.817	0.817	0.013
	F1-weighted	0.825	0.853	0.832	0.811	0.829	0.830	0.015
	AUC-macro	0.953	0.951	0.950	0.948	0.959	0.952	0.004
	AUC-weighted	0.950	0.950	0.944	0.944	0.957	0.949	0.005
KNN	ACC	0.776	0.776	0.781	0.781	0.771	0.777	0.004
	F1-macro	0.656	0.655	0.658	0.646	0.648	0.652	0.005
	F1-weighted	0.706	0.708	0.710	0.721	0.702	0.710	0.007
	AUC-macro	0.900	0.934	0.890	0.919	0.910	0.911	0.017
	AUC-weighted	0.912	0.929	0.889	0.922	0.916	0.914	0.015
SVM	ACC	0.886	0.886	0.881	0.891	0.905	0.890	0.010
	F1-macro	0.852	0.868	0.846	0.864	0.884	0.863	0.015

Models	Metrics	1	2	3	4	5	Mean	SD
	F1-weighted	0.883	0.885	0.877	0.886	0.904	0.887	0.010
	AUC-macro	0.978	0.974	0.983	0.974	0.981	0.978	0.004
	AUC-weighted	0.976	0.970	0.980	0.973	0.980	0.976	0.005
NB	ACC	0.786	0.741	0.761	0.726	0.751	0.753	0.022
	F1-macro	0.706	0.703	0.741	0.662	0.697	0.702	0.028
	F1-weighted	0.792	0.751	0.770	0.738	0.767	0.764	0.020
	AUC-macro	0.856	0.874	0.863	0.849	0.862	0.861	0.009
	AUC-weighted	0.901	0.895	0.878	0.880	0.876	0.886	0.011
RF	ACC	0.896	0.881	0.891	0.876	0.881	0.885	0.008
	F1-macro	0.856	0.876	0.863	0.853	0.847	0.859	0.011
	F1-weighted	0.891	0.879	0.886	0.869	0.878	0.880	0.008
	AUC-macro	0.983	0.975	0.979	0.971	0.975	0.977	0.005
	AUC-weighted	0.981	0.971	0.975	0.968	0.975	0.974	0.005
MOGONET	ACC	0.836	0.876	0.826	0.876	0.876	0.858	0.025
	F1-macro	0.739	0.837	0.747	0.835	0.845	0.800	0.053
	F1-weighted	0.821	0.872	0.816	0.870	0.871	0.850	0.029
	AUC-macro	0.905	0.951	0.922	0.938	0.969	0.937	0.025
	AUC-weighted	0.920	0.957	0.917	0.947	0.964	0.941	0.022
MCOF	ACC	0.866	0.891	0.920	0.910	0.910	0.900	0.022
	F1-macro	0.823	0.872	0.914	0.884	0.889	0.877	0.033
	F1-weighted	0.867	0.890	0.921	0.905	0.913	0.899	0.021
	AUC-macro	0.976	0.982	0.986	0.982	0.983	0.982	0.003
	AUC-weighted	0.974	0.978	0.982	0.979	0.983	0.979	0.004
**STAD**								
DIABLO	ACC	0.773	0.841	0.614	0.727	0.773	0.745	0.084
	F1-macro	0.735	0.819	0.581	0.692	0.733	0.712	0.086
	F1-weighted	0.782	0.845	0.637	0.744	0.776	0.757	0.076
	AUC-macro	0.883	0.938	0.836	0.907	0.887	0.890	0.037
	AUC-weighted	0.894	0.951	0.860	0.922	0.905	0.907	0.034
KNN	ACC	0.636	0.750	0.682	0.705	0.591	0.673	0.061
	F1-macro	0.530	0.673	0.519	0.558	0.459	0.548	0.079
	F1-weighted	0.609	0.725	0.622	0.651	0.548	0.631	0.065

Models	Metrics	1	2	3	4	5	Mean	SD
SVM	AUC-macro	0.818	0.845	0.817	0.847	0.829	0.831	0.014
	AUC-weighted	0.815	0.838	0.835	0.858	0.822	0.834	0.016
	ACC	0.773	0.864	0.773	0.864	0.818	0.818	0.045
	F1-macro	0.702	0.847	0.721	0.840	0.786	0.779	0.066
	F1-weighted	0.762	0.861	0.757	0.868	0.813	0.812	0.053
	AUC-macro	0.880	0.962	0.905	0.927	0.928	0.921	0.030
	AUC-weighted	0.880	0.960	0.900	0.942	0.928	0.922	0.032
NB	ACC	0.341	0.545	0.455	0.455	0.455	0.450	0.073
	F1-macro	0.242	0.482	0.375	0.390	0.324	0.363	0.088
	F1-weighted	0.336	0.546	0.456	0.460	0.431	0.446	0.075
	AUC-macro	0.465	0.596	0.513	0.551	0.515	0.528	0.049
	AUC-weighted	0.451	0.595	0.534	0.551	0.518	0.530	0.052
RF	ACC	0.727	0.841	0.750	0.864	0.795	0.795	0.058
	F1-macro	0.636	0.818	0.687	0.839	0.732	0.742	0.086
	F1-weighted	0.688	0.835	0.730	0.858	0.778	0.778	0.071
	AUC-macro	0.882	0.975	0.909	0.913	0.925	0.921	0.034
	AUC-weighted	0.876	0.970	0.909	0.929	0.924	0.922	0.034
MOGONET	ACC	0.682	0.795	0.705	0.750	0.727	0.732	0.044
	F1-macro	0.588	0.784	0.627	0.687	0.648	0.667	0.075
	F1-weighted	0.648	0.801	0.684	0.736	0.704	0.715	0.058
	AUC-macro	0.705	0.869	0.782	0.848	0.800	0.801	0.064
	AUC-weighted	0.698	0.864	0.782	0.837	0.796	0.795	0.063
MCOF	ACC	0.773	0.841	0.818	0.818	0.773	0.805	0.030
	F1-macro	0.754	0.836	0.789	0.799	0.753	0.786	0.035
	F1-weighted	0.778	0.842	0.808	0.825	0.778	0.806	0.028
	AUC-macro	0.926	0.960	0.951	0.911	0.924	0.934	0.020
	AUC-weighted	0.920	0.952	0.940	0.925	0.914	0.930	0.016

Table S2 Classification performance of MCOF and its ablated variants across five independent repetitions on four multi-omics datasets.

Panel A. ROSMAP

Model	Repeat	ACC	F1	AUC	MCC	Balanced ACC	Loss
MCOF	1	0.690	0.694	0.828	0.381	0.691	1.133
	2	0.746	0.735	0.849	0.503	0.750	1.293
	3	0.761	0.773	0.867	0.520	0.760	1.400
	4	0.803	0.788	0.850	0.624	0.807	1.172
	5	0.746	0.763	0.784	0.492	0.745	0.709
MCOF w/o channel attention	1	0.746	0.780	0.829	0.500	0.741	1.013
	2	0.746	0.775	0.847	0.496	0.742	1.083
	3	0.789	0.795	0.859	0.577	0.789	1.130
	4	0.775	0.784	0.831	0.548	0.774	0.938
	5	0.817	0.835	0.882	0.637	0.814	0.868
MCOF w/o convolution	1	0.746	0.750	0.827	0.494	0.747	1.425
	2	0.690	0.686	0.800	0.385	0.692	2.066
	3	0.718	0.744	0.863	0.436	0.715	1.234
	4	0.732	0.716	0.821	0.478	0.736	1.882
	5	0.732	0.753	0.859	0.464	0.730	1.373

Panel B. SCZ

Model	Repeat	ACC	F1	AUC	MCC	Balanced ACC	Loss
MCOF	1	0.857	0.857	0.891	0.718	0.859	0.715
	2	0.810	0.833	0.955	0.626	0.805	0.356
	3	0.810	0.800	0.945	0.633	0.814	0.541
	4	0.762	0.737	0.927	0.552	0.768	0.552
	5	0.857	0.842	1.000	0.748	0.864	0.220
MCOF w/o channel attention	1	0.714	0.727	0.836	0.427	0.714	1.220
	2	0.952	0.957	0.991	0.908	0.950	0.155
	3	0.905	0.909	0.927	0.809	0.905	0.589
	4	0.857	0.880	0.936	0.742	0.850	0.467
	5	0.905	0.900	0.991	0.826	0.909	0.361
MCOF w/o convolution	1	0.714	0.700	0.827	0.440	0.718	2.577
	2	0.905	0.909	0.900	0.809	0.905	0.440
	3	0.857	0.842	0.945	0.748	0.864	0.630
	4	0.857	0.857	0.955	0.718	0.859	0.482
	5	0.905	0.900	0.973	0.826	0.909	0.786

Panel C. BRCA

Model	Repeat	ACC	Macro F1	Weighted F1	Macro AUC	Weighted AUC	Balanced ACC	Loss
MCOF	1	0.866	0.823	0.867	0.976	0.974	0.821	0.768
	2	0.891	0.872	0.890	0.982	0.978	0.891	0.534
	3	0.920	0.914	0.921	0.986	0.982	0.921	0.291
	4	0.910	0.884	0.905	0.982	0.979	0.888	0.556
	5	0.910	0.889	0.913	0.983	0.983	0.912	0.509
MCOF w/o channel attention	1	0.900	0.875	0.899	0.978	0.978	0.860	0.565
	2	0.886	0.864	0.886	0.976	0.973	0.889	0.794
	3	0.915	0.906	0.915	0.982	0.978	0.906	0.503
	4	0.905	0.888	0.904	0.977	0.975	0.889	0.599
	5	0.886	0.857	0.888	0.981	0.982	0.875	0.635
MCOF w/o convolution	1	0.881	0.857	0.879	0.983	0.981	0.864	0.609
	2	0.886	0.868	0.888	0.982	0.979	0.879	0.693
	3	0.891	0.841	0.889	0.981	0.979	0.832	0.670
	4	0.891	0.880	0.890	0.968	0.965	0.878	0.810
	5	0.910	0.894	0.912	0.986	0.985	0.903	0.331

Panel D. STAD

Model	Repeat	ACC	Macro F1	Weighted F1	Macro AUC	Weighted AUC	Balanced ACC	Loss
	1	0.773	0.754	0.778	0.926	0.920	0.783	1.205
	2	0.841	0.836	0.842	0.960	0.952	0.843	0.615
MCOF	3	0.818	0.789	0.808	0.951	0.940	0.770	0.588
	4	0.818	0.799	0.825	0.911	0.925	0.830	1.181
	5	0.773	0.753	0.778	0.924	0.914	0.779	1.192
	1	0.727	0.705	0.730	0.877	0.873	0.736	2.130
	2	0.818	0.792	0.817	0.947	0.937	0.786	1.021
MCOF w/o channel attention	3	0.773	0.767	0.783	0.923	0.916	0.803	1.061
	4	0.864	0.842	0.863	0.953	0.956	0.833	0.752
	5	0.727	0.703	0.736	0.911	0.907	0.733	1.389
	1	0.795	0.783	0.796	0.897	0.890	0.820	1.594
	2	0.841	0.818	0.835	0.954	0.944	0.779	1.064
MCOF w/o convolution	3	0.795	0.779	0.800	0.936	0.930	0.820	0.794
	4	0.909	0.886	0.911	0.930	0.933	0.907	0.786
	5	0.795	0.779	0.798	0.932	0.926	0.793	0.925

Note: MCOF without channel attention denotes the ablated variant in which the channel-attention module was removed, whereas MCOF without convolution denotes the variant in which the convolutional module was removed. Loss denotes the mean cross-entropy loss on the external test set; lower values indicate better probabilistic performance, whereas higher values indicate better performance for all other metrics.

Table S3 Computational complexity and inference efficiency of MCOF and MOGONET across four datasets

Dataset	Model	Repeats	Trainable parameters	Forward pass, ms/test set	Latency, ms/sample	Peak allocated memory, MB
ROSMAP	MCOF	5	744,712	1.796 ± 0.008	0.025 ± 0.000	19.194 ± 0.000
	MOGONET	5	302,196	2.578 ± 0.006	0.036 ± 0.000	11.484 ± 0.004
SCZ	MCOF	5	685,446	0.792 ± 0.004	0.038 ± 0.000	15.084 ± 0.000
	MOGONET	5	201,434	1.748 ± 0.005	0.083 ± 0.000	10.110 ± 0.001
BRCA	MCOF	5	890,760	3.063 ± 0.013	0.015 ± 0.000	21.618 ± 0.000
	MOGONET	5	1,283,948	2.087 ± 0.022	0.010 ± 0.000	28.606 ± 0.022
STAD	MCOF	5	890,503	0.789 ± 0.005	0.018 ± 0.000	18.964 ± 0.000
	MOGONET	5	1,283,326	1.818 ± 0.006	0.041 ± 0.000	16.409 ± 0.002

Note: Timing, latency, and peak allocated GPU memory are reported as mean ± SD across five repeats. Trainable parameters are fixed model counts. All computational metrics are shown to three decimal places. All benchmarks were conducted under identical hardware and software settings.

Table S4 Cross-repeat stability of biomarkers identified by MCOF using integrated gradients

Panel A. ROSMAP

Stability rank	Biomarker	Omics	Mean |IG|	Median rank	Rank range	Top 50, n (frequency)	Top 100, n (frequency)	Top 200, n (frequency)
1	cg19485804	DNA methylation	0.263	30.000	16–75	4 (0.800)	5 (1.000)	5 (1.000)
2	ENSG00000168743.8	mRNA	0.408	1.000	1–123	4 (0.800)	4 (0.800)	5 (1.000)
3	ENSG00000142765.12	mRNA	0.264	35.000	4–209	4 (0.800)	4 (0.800)	4 (0.800)
4	ENSG00000242349.1	mRNA	0.263	21.000	2–366	4 (0.800)	4 (0.800)	4 (0.800)
5	hsa-miR-132	miRNA	0.275	31.000	3–463	4 (0.800)	4 (0.800)	4 (0.800)
6	cg27091787	DNA methylation	0.243	36.000	4–165	3 (0.600)	4 (0.800)	5 (1.000)
7	cg12981137	DNA methylation	0.227	42.000	29–134	3 (0.600)	4 (0.800)	5 (1.000)
8	cg00745735	DNA methylation	0.244	36.000	5–245	3 (0.600)	4 (0.800)	4 (0.800)
9	cg03894103	DNA methylation	0.270	36.000	2–542	3 (0.600)	4 (0.800)	4 (0.800)
10	ENSG00000155980.6	mRNA	0.231	21.000	4–213	3 (0.600)	3 (0.600)	4 (0.800)
11	ENSG00000196415.4	mRNA	0.256	43.000	3–324	3 (0.600)	3 (0.600)	4 (0.800)

Stability rank	Biomarker	Omics	Mean |IG|	Median rank	Rank range	Top 50, n (frequency)	Top 100, n (frequency)	Top 200, n (frequency)
12	ebv-miR-BART8	miRNA	0.230	24.000	14–314	3 (0.600)	3 (0.600)	3 (0.600)
13	cg24192663	DNA methylation	0.231	38.000	9–411	3 (0.600)	3 (0.600)	4 (0.800)
14	hsa-miR-146b-5p	miRNA	0.236	42.000	5–436	3 (0.600)	3 (0.600)	4 (0.800)
15	hsa-miR-885-5p	miRNA	0.243	43.000	6–493	3 (0.600)	3 (0.600)	4 (0.800)
16	ENSG00000182902.8	mRNA	0.172	40.000	24–497	3 (0.600)	3 (0.600)	3 (0.600)
17	hsa-miR-129-5p	miRNA	0.217	68.000	15–93	2 (0.400)	5 (1.000)	5 (1.000)
18	cg14847483	DNA methylation	0.212	51.000	29–166	2 (0.400)	4 (0.800)	5 (1.000)
19	ebv-miR-BART6-5p	miRNA	0.184	70.000	18–337	2 (0.400)	4 (0.800)	4 (0.800)
20	hsa-miR-29a	miRNA	0.251	54.000	10–418	2 (0.400)	4 (0.800)	4 (0.800)
21	cg07992625	DNA methylation	0.216	63.000	38–356	2 (0.400)	4 (0.800)	4 (0.800)
22	ENSG00000053702.10	mRNA	0.213	77.000	2–148	2 (0.400)	3 (0.600)	5 (1.000)
23	cg20793071	DNA methylation	0.233	84.000	7–235	2 (0.400)	3 (0.600)	4 (0.800)
24	hsa-miR-133a	miRNA	0.215	72.000	11–307	2 (0.400)	3 (0.600)	4 (0.800)

Stability rank	Biomarker	Omics	Mean |IG|	Median rank	Rank range	Top 50, n (frequency)	Top 100, n (frequency)	Top 200, n (frequency)
25	cg17237063	DNA methylation	0.209	60.000	14–328	2 (0.400)	3 (0.600)	3 (0.600)
26	cg04983977	DNA methylation	0.189	99.000	3–327	2 (0.400)	3 (0.600)	3 (0.600)
27	ENSG00000170276.4	mRNA	0.208	94.000	20–344	2 (0.400)	3 (0.600)	3 (0.600)
28	hsa-miR-335	miRNA	0.170	94.000	12–380	2 (0.400)	3 (0.600)	3 (0.600)
29	hcmv-miR-UL70-3p	miRNA	0.210	117.000	17–214	2 (0.400)	2 (0.400)	4 (0.800)
30	ENSG00000180777.9	mRNA	0.173	136.000	10–310	2 (0.400)	2 (0.400)	3 (0.600)
31	cg17886959	DNA methylation	0.209	158.000	16–382	2 (0.400)	2 (0.400)	4 (0.800)
32	cg17385448	DNA methylation	0.200	112.000	18–438	2 (0.400)	2 (0.400)	4 (0.800)
33	hsa-miR-34b	miRNA	0.209	122.000	14–548	2 (0.400)	2 (0.400)	4 (0.800)
34	hsa-miR-1266	miRNA	0.171	209.000	33–320	2 (0.400)	2 (0.400)	2 (0.400)
35	cg02085507	DNA methylation	0.150	255.000	11–409	2 (0.400)	2 (0.400)	2 (0.400)
36	ENSG00000086159.8	mRNA	0.152	214.000	23–383	2 (0.400)	2 (0.400)	2 (0.400)
37	hsa-miR-375	miRNA	0.203	290.000	6–362	2 (0.400)	2 (0.400)	2 (0.400)

Stability rank	Biomarker	Omics	Mean |IG|	Median rank	Rank range	Top 50, n (frequency)	Top 100, n (frequency)	Top 200, n (frequency)
38	cg25681177	DNA methylation	0.212	136.000	8–558	2 (0.400)	2 (0.400)	3 (0.600)
39	ENSG00000235501.1	mRNA	0.177	228.000	3–461	2 (0.400)	2 (0.400)	2 (0.400)
40	hsa-miR-379	miRNA	0.165	272.000	26–481	2 (0.400)	2 (0.400)	2 (0.400)
41	ENSG00000235823.1	mRNA	0.158	203.000	5–579	2 (0.400)	2 (0.400)	2 (0.400)
42	cg00754253	DNA methylation	0.203	294.000	7–445	2 (0.400)	2 (0.400)	2 (0.400)
43	hsa-miR-1	miRNA	0.185	218.000	17–557	2 (0.400)	2 (0.400)	2 (0.400)
44	ENSG00000065361.8	mRNA	0.175	275.000	6–584	2 (0.400)	2 (0.400)	2 (0.400)
45	cg24101578	DNA methylation	0.182	266.000	33–585	2 (0.400)	2 (0.400)	2 (0.400)
46	cg21838334	DNA methylation	0.172	277.000	40–598	2 (0.400)	2 (0.400)	2 (0.400)
47	cg00777121	DNA methylation	0.157	378.000	28–591	2 (0.400)	2 (0.400)	2 (0.400)
48	ENSG00000010310.4	mRNA	0.143	559.000	9–571	2 (0.400)	2 (0.400)	2 (0.400)
49	hsa-miR-126	miRNA	0.191	85.000	32–443	1 (0.200)	4 (0.800)	4 (0.800)
50	hsa-miR-23b	miRNA	0.171	99.000	6–572	1 (0.200)	4 (0.800)	4 (0.800)

Stability rank	Biomarker	Omics	Mean |IG|	Median rank	Rank range	Top 50, n (frequency)	Top 100, n (frequency)	Top 200, n (frequency)
51	hsa-miR-520e	miRNA	0.210	84.000	19–244	1 (0.200)	3 (0.600)	4 (0.800)
52	cg18001427	DNA methylation	0.197	90.000	30–262	1 (0.200)	3 (0.600)	4 (0.800)
53	cg10157098	DNA methylation	0.187	76.000	43–398	1 (0.200)	3 (0.600)	4 (0.800)
54	ENSG00000125534.4	mRNA	0.184	87.000	45–414	1 (0.200)	3 (0.600)	3 (0.600)
55	hsa-miR-517c_hsa-miR-519a	miRNA	0.191	76.000	36–473	1 (0.200)	3 (0.600)	4 (0.800)
56	cg06258834	DNA methylation	0.194	97.000	28–410	1 (0.200)	3 (0.600)	3 (0.600)
57	cg08952029	DNA methylation	0.182	72.000	37–593	1 (0.200)	3 (0.600)	3 (0.600)
58	ENSG00000116786.7	mRNA	0.149	92.000	37–541	1 (0.200)	3 (0.600)	3 (0.600)
59	ENSG00000198830.6	mRNA	0.175	119.000	37–214	1 (0.200)	2 (0.400)	4 (0.800)
60	ENSG00000171388.9	mRNA	0.201	106.000	28–302	1 (0.200)	2 (0.400)	4 (0.800)
61	ENSG00000267488.1	mRNA	0.203	111.000	13–290	1 (0.200)	2 (0.400)	4 (0.800)
62	cg12864235	DNA methylation	0.205	112.000	11–239	1 (0.200)	2 (0.400)	3 (0.600)
63	hsv1-miR-H3	miRNA	0.184	137.000	46–265	1 (0.200)	2 (0.400)	4 (0.800)

Stability rank	Biomarker	Omics	Mean |IG|	Median rank	Rank range	Top 50, n (frequency)	Top 100, n (frequency)	Top 200, n (frequency)
64	hsa-miR-199a-5p	miRNA	0.160	156.000	47–216	1 (0.200)	2 (0.400)	3 (0.600)
65	cg19832721	DNA methylation	0.205	142.000	21–343	1 (0.200)	2 (0.400)	4 (0.800)
66	cg01182697	DNA methylation	0.162	110.000	30–319	1 (0.200)	2 (0.400)	4 (0.800)
67	hsa-miR-548a-5p	miRNA	0.192	131.000	16–284	1 (0.200)	2 (0.400)	3 (0.600)
68	ENSG00000236609.3	mRNA	0.173	134.000	35–324	1 (0.200)	2 (0.400)	4 (0.800)
69	cg00095526	DNA methylation	0.182	138.000	45–259	1 (0.200)	2 (0.400)	3 (0.600)
70	ENSG00000196517.7	mRNA	0.174	149.000	22–293	1 (0.200)	2 (0.400)	3 (0.600)
71	cg00398048	DNA methylation	0.160	144.000	33–402	1 (0.200)	2 (0.400)	4 (0.800)
72	ENSG00000254858.2	mRNA	0.169	158.000	19–293	1 (0.200)	2 (0.400)	3 (0.600)
73	cg18220560	DNA methylation	0.173	124.000	46–426	1 (0.200)	2 (0.400)	4 (0.800)
74	cg15127733	DNA methylation	0.200	240.000	5–286	1 (0.200)	2 (0.400)	2 (0.400)
75	hsa-miR-548a-3p	miRNA	0.182	114.000	41–388	1 (0.200)	2 (0.400)	3 (0.600)

Stability rank	Biomarker	Omics	Mean |IG|	Median rank	Rank range	Top 50, n (frequency)	Top 100, n (frequency)	Top 200, n (frequency)
76	kshv-miR-K12-4-5p	miRNA	0.194	113.000	21–431	1 (0.200)	2 (0.400)	3 (0.600)
77	ENSG00000144057.11	mRNA	0.181	158.000	50–455	1 (0.200)	2 (0.400)	4 (0.800)
78	hsa-miR-577	miRNA	0.176	115.000	18–396	1 (0.200)	2 (0.400)	3 (0.600)
79	cg03453449	DNA methylation	0.193	140.000	19–533	1 (0.200)	2 (0.400)	4 (0.800)
80	cg04126866	DNA methylation	0.210	101.000	6–518	1 (0.200)	2 (0.400)	3 (0.600)
81	cg14387505	DNA methylation	0.180	138.000	23–448	1 (0.200)	2 (0.400)	3 (0.600)
82	cg15517609	DNA methylation	0.164	114.000	12–593	1 (0.200)	2 (0.400)	3 (0.600)
83	ENSG00000105643.3	mRNA	0.175	170.000	26–427	1 (0.200)	2 (0.400)	3 (0.600)
84	cg06690548	DNA methylation	0.138	193.000	45–597	1 (0.200)	2 (0.400)	3 (0.600)
85	cg16003238	DNA methylation	0.167	275.000	15–447	1 (0.200)	2 (0.400)	2 (0.400)
86	hsa-miR-660	miRNA	0.166	262.000	17–473	1 (0.200)	2 (0.400)	2 (0.400)
87	hsa-miR-551b	miRNA	0.140	232.000	25–501	1 (0.200)	2 (0.400)	2 (0.400)

Stability rank	Biomarker	Omics	Mean |IG|	Median rank	Rank range	Top 50, n (frequency)	Top 100, n (frequency)	Top 200, n (frequency)
88	cg14613972	DNA methylation	0.194	283.000	4–428	1 (0.200)	2 (0.400)	2 (0.400)
89	cg15013019	DNA methylation	0.172	194.000	49–588	1 (0.200)	2 (0.400)	3 (0.600)
90	hsa-miR-423-3p	miRNA	0.166	318.000	26–435	1 (0.200)	2 (0.400)	2 (0.400)
91	ENSG00000177025.2	mRNA	0.150	147.000	48–568	1 (0.200)	2 (0.400)	3 (0.600)
92	cg08367223	DNA methylation	0.181	334.000	10–504	1 (0.200)	2 (0.400)	2 (0.400)
93	ENSG00000129250.7	mRNA	0.142	223.000	39–534	1 (0.200)	2 (0.400)	2 (0.400)
94	cg02654291	DNA methylation	0.138	406.000	8–475	1 (0.200)	2 (0.400)	2 (0.400)
95	cg24322623	DNA methylation	0.189	384.000	2–512	1 (0.200)	2 (0.400)	2 (0.400)
96	hsa-miR-34c-5p	miRNA	0.189	113.000	9–164	1 (0.200)	1 (0.200)	5 (1.000)
97	ENSG00000198624.8	mRNA	0.171	127.000	37–187	1 (0.200)	1 (0.200)	5 (1.000)
98	cg13468685	DNA methylation	0.222	107.000	3–296	1 (0.200)	1 (0.200)	4 (0.800)
99	cg11761535	DNA methylation	0.188	123.000	44–234	1 (0.200)	1 (0.200)	4 (0.800)

Stability rank	Biomarker	Omics	Mean |IG|	Median rank	Rank range	Top 50, n (frequency)	Top 100, n (frequency)	Top 200, n (frequency)
100	cg18149207	DNA methylation	0.183	133.000	41–223	1 (0.200)	1 (0.200)	4 (0.800)

Panel B. SCZ

Stability rank	Biomarker	Omics	Mean |IG|	Median rank	Rank range	Top 50, n (frequency)	Top 100, n (frequency)	Top 200, n (frequency)
1	P27169	Protein	0.199	38.000	17–100	4 (0.800)	5 (1.000)	5 (1.000)
2	Q15166	Protein	0.199	31.000	15–113	4 (0.800)	4 (0.800)	5 (1.000)
3	P49747	Protein	0.188	40.000	7–197	4 (0.800)	4 (0.800)	5 (1.000)
4	P24593	Protein	0.185	37.000	16–184	3 (0.600)	4 (0.800)	5 (1.000)
5	P01857	Protein	0.195	50.000	14–169	3 (0.600)	4 (0.800)	5 (1.000)
6	P01344	Protein	0.203	24.000	4–198	3 (0.600)	4 (0.800)	5 (1.000)
7	Q9UHG3	Protein	0.214	48.000	1–251	3 (0.600)	4 (0.800)	4 (0.800)
8	182	Metabolite	0.186	46.000	13–106	3 (0.600)	3 (0.600)	5 (1.000)
9	226	Metabolite	0.180	46.000	19–134	3 (0.600)	3 (0.600)	5 (1.000)
10	P49908	Protein	0.177	50.000	15–156	3 (0.600)	3 (0.600)	5 (1.000)
11	1036	Metabolite	0.176	48.000	20–200	3 (0.600)	3 (0.600)	5 (1.000)
12	629	Metabolite	0.175	47.000	8–236	3 (0.600)	3 (0.600)	4 (0.800)
13	P35908	Protein	0.170	45.000	21–216	3 (0.600)	3 (0.600)	4 (0.800)
14	P22792	Protein	0.194	47.000	2–268	3 (0.600)	3 (0.600)	4 (0.800)
15	P06396	Protein	0.163	46.000	27–245	3 (0.600)	3 (0.600)	3 (0.600)
16	P00738	Protein	0.205	18.000	3–367	3 (0.600)	3 (0.600)	3 (0.600)

Stability rank	Biomarker	Omics	Mean |IG|	Median rank	Rank range	Top 50, n (frequency)	Top 100, n (frequency)	Top 200, n (frequency)
17	657	Metabolite	0.204	54.000	7–113	2 (0.400)	4 (0.800)	5 (1.000)
18	P35443	Protein	0.193	76.000	4–168	2 (0.400)	4 (0.800)	5 (1.000)
19	H7C013	Protein	0.203	67.000	1–200	2 (0.400)	4 (0.800)	5 (1.000)
20	P01019	Protein	0.174	73.000	13–169	2 (0.400)	4 (0.800)	5 (1.000)
21	P17936	Protein	0.194	64.000	9–285	2 (0.400)	4 (0.800)	4 (0.800)
22	408	Metabolite	0.167	55.000	28–267	2 (0.400)	4 (0.800)	4 (0.800)
23	Q14624	Protein	0.174	78.000	7–296	2 (0.400)	4 (0.800)	4 (0.800)
24	E9PFZ2	Protein	0.179	57.000	12–161	2 (0.400)	3 (0.600)	5 (1.000)
25	683	Metabolite	0.190	82.000	6–191	2 (0.400)	3 (0.600)	5 (1.000)
26	1032	Metabolite	0.181	62.000	2–228	2 (0.400)	3 (0.600)	4 (0.800)
27	992	Metabolite	0.166	58.000	33–203	2 (0.400)	3 (0.600)	4 (0.800)
28	336	Metabolite	0.185	75.000	2–254	2 (0.400)	3 (0.600)	4 (0.800)
29	P25774	Protein	0.207	82.000	5–289	2 (0.400)	3 (0.600)	4 (0.800)
30	1443	Metabolite	0.185	62.000	3–247	2 (0.400)	3 (0.600)	4 (0.800)
31	Q9Y6Z7	Protein	0.176	91.000	15–265	2 (0.400)	3 (0.600)	4 (0.800)
32	E7ETH0	Protein	0.191	85.000	8–264	2 (0.400)	3 (0.600)	4 (0.800)
33	1474	Metabolite	0.172	79.000	6–248	2 (0.400)	3 (0.600)	4 (0.800)

Stability rank	Biomarker	Omics	Mean |IG|	Median rank	Rank range	Top 50, n (frequency)	Top 100, n (frequency)	Top 200, n (frequency)
34	1038	Metabolite	0.158	89.000	23–264	2 (0.400)	3 (0.600)	4 (0.800)
35	P01011	Protein	0.161	53.000	44–302	2 (0.400)	3 (0.600)	4 (0.800)
36	59	Metabolite	0.183	58.000	10–299	2 (0.400)	3 (0.600)	4 (0.800)
37	A0A0C4DH38	Protein	0.173	80.000	13–262	2 (0.400)	3 (0.600)	3 (0.600)
38	P05090	Protein	0.162	93.000	15–273	2 (0.400)	3 (0.600)	3 (0.600)
39	704	Metabolite	0.210	66.000	2–305	2 (0.400)	3 (0.600)	3 (0.600)
40	H0Y300	Protein	0.160	90.000	26–292	2 (0.400)	3 (0.600)	3 (0.600)
41	Q13790	Protein	0.163	55.000	20–356	2 (0.400)	3 (0.600)	4 (0.800)
42	Q9NPH3	Protein	0.177	76.000	3–346	2 (0.400)	3 (0.600)	3 (0.600)
43	P00748	Protein	0.204	107.000	6–118	2 (0.400)	2 (0.400)	5 (1.000)
44	Q16610	Protein	0.229	155.000	1–190	2 (0.400)	2 (0.400)	5 (1.000)
45	P01859	Protein	0.181	117.000	9–224	2 (0.400)	2 (0.400)	4 (0.800)
46	P00450	Protein	0.156	135.000	41–176	2 (0.400)	2 (0.400)	5 (1.000)
47	Q06033	Protein	0.160	160.000	11–244	2 (0.400)	2 (0.400)	4 (0.800)
48	P20851	Protein	0.171	106.000	11–243	2 (0.400)	2 (0.400)	3 (0.600)
49	367	Metabolite	0.157	178.000	14–205	2 (0.400)	2 (0.400)	4 (0.800)
50	P33151	Protein	0.182	134.000	8–313	2 (0.400)	2 (0.400)	4 (0.800)

Stability rank	Biomarker	Omics	Mean |IG|	Median rank	Rank range	Top 50, n (frequency)	Top 100, n (frequency)	Top 200, n (frequency)
51	1259	Metabolite	0.163	152.000	15–231	2 (0.400)	2 (0.400)	3 (0.600)
52	P00747	Protein	0.157	110.000	38–285	2 (0.400)	2 (0.400)	4 (0.800)
53	296	Metabolite	0.183	141.000	4–364	2 (0.400)	2 (0.400)	4 (0.800)
54	P05362	Protein	0.174	153.000	11–263	2 (0.400)	2 (0.400)	3 (0.600)
55	400	Metabolite	0.176	208.000	4–263	2 (0.400)	2 (0.400)	2 (0.400)
56	1124	Metabolite	0.157	194.000	14–305	2 (0.400)	2 (0.400)	3 (0.600)
57	829	Metabolite	0.150	154.000	19–349	2 (0.400)	2 (0.400)	3 (0.600)
58	973	Metabolite	0.148	193.000	40–356	2 (0.400)	2 (0.400)	3 (0.600)
59	P25311	Protein	0.169	271.000	12–310	2 (0.400)	2 (0.400)	2 (0.400)
60	9	Metabolite	0.188	68.000	5–108	1 (0.200)	4 (0.800)	5 (1.000)
61	987	Metabolite	0.164	78.000	30–274	1 (0.200)	4 (0.800)	4 (0.800)
62	890	Metabolite	0.174	67.000	9–177	1 (0.200)	3 (0.600)	5 (1.000)
63	A0A087WWT3	Protein	0.165	86.000	13–149	1 (0.200)	3 (0.600)	5 (1.000)
64	165	Metabolite	0.180	99.000	3–145	1 (0.200)	3 (0.600)	5 (1.000)
65	906	Metabolite	0.183	65.000	6–220	1 (0.200)	3 (0.600)	4 (0.800)
66	236	Metabolite	0.164	52.000	17–222	1 (0.200)	3 (0.600)	4 (0.800)
67	838	Metabolite	0.162	88.000	45–178	1 (0.200)	3 (0.600)	5 (1.000)

Stability rank	Biomarker	Omics	Mean |IG|	Median rank	Rank range	Top 50, n (frequency)	Top 100, n (frequency)	Top 200, n (frequency)
68	950	Metabolite	0.154	94.000	39–198	1 (0.200)	3 (0.600)	5 (1.000)
69	38	Metabolite	0.156	89.000	49–203	1 (0.200)	3 (0.600)	4 (0.800)
70	P01034	Protein	0.183	86.000	4–230	1 (0.200)	3 (0.600)	4 (0.800)
71	Q6UXB8	Protein	0.169	84.000	16–315	1 (0.200)	3 (0.600)	4 (0.800)
72	774	Metabolite	0.147	86.000	35–276	1 (0.200)	3 (0.600)	4 (0.800)
73	P08514	Protein	0.149	73.000	41–325	1 (0.200)	3 (0.600)	4 (0.800)
74	30	Metabolite	0.152	67.000	35–253	1 (0.200)	3 (0.600)	3 (0.600)
75	P10909	Protein	0.151	63.000	40–274	1 (0.200)	3 (0.600)	3 (0.600)
76	541	Metabolite	0.145	97.000	34–350	1 (0.200)	3 (0.600)	4 (0.800)
77	207	Metabolite	0.143	67.000	19–339	1 (0.200)	3 (0.600)	3 (0.600)
78	Q12805	Protein	0.167	133.000	22–177	1 (0.200)	2 (0.400)	5 (1.000)
79	P13671	Protein	0.157	106.000	27–238	1 (0.200)	2 (0.400)	4 (0.800)
80	Q15063	Protein	0.154	127.000	22–180	1 (0.200)	2 (0.400)	5 (1.000)
81	331	Metabolite	0.155	143.000	9–204	1 (0.200)	2 (0.400)	4 (0.800)
82	P41222	Protein	0.158	114.000	12–277	1 (0.200)	2 (0.400)	4 (0.800)
83	192	Metabolite	0.153	140.000	40–193	1 (0.200)	2 (0.400)	5 (1.000)
84	157	Metabolite	0.148	111.000	43–213	1 (0.200)	2 (0.400)	4 (0.800)

Stability rank	Biomarker	Omics	Mean |IG|	Median rank	Rank range	Top 50, n (frequency)	Top 100, n (frequency)	Top 200, n (frequency)
85	464	Metabolite	0.151	122.000	36–223	1 (0.200)	2 (0.400)	4 (0.800)
86	345	Metabolite	0.152	102.000	42–294	1 (0.200)	2 (0.400)	4 (0.800)
87	P00740	Protein	0.174	177.000	7–195	1 (0.200)	2 (0.400)	5 (1.000)
88	G3XAK1	Protein	0.150	107.000	47–250	1 (0.200)	2 (0.400)	4 (0.800)
89	248	Metabolite	0.150	144.000	23–243	1 (0.200)	2 (0.400)	4 (0.800)
90	P01714	Protein	0.155	128.000	13–270	1 (0.200)	2 (0.400)	4 (0.800)
91	670	Metabolite	0.147	140.000	25–282	1 (0.200)	2 (0.400)	4 (0.800)
92	454	Metabolite	0.149	104.000	45–246	1 (0.200)	2 (0.400)	4 (0.800)
93	467	Metabolite	0.152	158.000	17–242	1 (0.200)	2 (0.400)	4 (0.800)
94	B4DPQ0	Protein	0.151	144.000	31–259	1 (0.200)	2 (0.400)	4 (0.800)
95	A0A075B6J9	Protein	0.155	134.000	11–260	1 (0.200)	2 (0.400)	4 (0.800)
96	P06681	Protein	0.142	145.000	50–202	1 (0.200)	2 (0.400)	4 (0.800)
97	P20742	Protein	0.146	128.000	37–323	1 (0.200)	2 (0.400)	4 (0.800)
98	244	Metabolite	0.141	193.000	46–240	1 (0.200)	2 (0.400)	3 (0.600)
99	775	Metabolite	0.146	127.000	26–345	1 (0.200)	2 (0.400)	3 (0.600)
100	141	Metabolite	0.146	101.000	33–335	1 (0.200)	2 (0.400)	3 (0.600)

Panel C. BRCA

Stability rank	Biomarker	Omics	Mean |IG|	Median rank	Rank range	Top 50, n (frequency)	Top 100, n (frequency)	Top 200, n (frequency)
1	hsa-mir-4664	miRNA	0.100	15.000	1–52	4 (0.800)	5 (1.000)	5 (1.000)
2	PPP1R14C	mRNA	0.088	25.000	2–82	4 (0.800)	5 (1.000)	5 (1.000)
3	VGLL1	mRNA	0.078	30.000	17–88	4 (0.800)	5 (1.000)	5 (1.000)
4	CDH3	mRNA	0.081	23.000	2–194	4 (0.800)	4 (0.800)	5 (1.000)
5	PHYHD1	mRNA	0.078	26.000	5–608	4 (0.800)	4 (0.800)	4 (0.800)
6	hsa-mir-548e	miRNA	0.079	34.000	7–216	3 (0.600)	4 (0.800)	4 (0.800)
7	LAMP5	mRNA	0.074	38.000	10–363	3 (0.600)	4 (0.800)	4 (0.800)
8	hsa-mir-185	miRNA	0.080	46.000	4–1115	3 (0.600)	4 (0.800)	4 (0.800)
9	hsa-let-7c	miRNA	0.075	41.000	7–186	3 (0.600)	3 (0.600)	5 (1.000)
10	FOXC1	mRNA	0.076	27.000	6–295	3 (0.600)	3 (0.600)	4 (0.800)
11	CXCL1	mRNA	0.072	35.000	21–291	3 (0.600)	3 (0.600)	3 (0.600)
12	hsa-mir-204	miRNA	0.076	31.000	9–421	3 (0.600)	3 (0.600)	4 (0.800)
13	hsa-mir-618	miRNA	0.083	17.000	1–695	3 (0.600)	3 (0.600)	4 (0.800)
14	hsa-mir-26a-1	miRNA	0.073	30.000	4–1136	3 (0.600)	3 (0.600)	3 (0.600)
15	hsa-mir-200b	miRNA	0.072	31.000	7–1249	3 (0.600)	3 (0.600)	3 (0.600)
16	hsa-mir-6764	miRNA	0.073	39.000	10–1090	3 (0.600)	3 (0.600)	3 (0.600)

Stability rank	Biomarker	Omics	Mean |IG|	Median rank	Rank range	Top 50, n (frequency)	Top 100, n (frequency)	Top 200, n (frequency)
17	TMEM215	mRNA	0.073	56.000	5–392	2 (0.400)	4 (0.800)	4 (0.800)
18	hsa-mir-4756	miRNA	0.072	53.000	16–793	2 (0.400)	3 (0.600)	4 (0.800)
19	PI3	mRNA	0.066	90.000	9–789	2 (0.400)	3 (0.600)	4 (0.800)
20	MID1	mRNA	0.074	61.000	1–999	2 (0.400)	3 (0.600)	3 (0.600)
21	hsa-mir-581	miRNA	0.066	83.000	13–1413	2 (0.400)	3 (0.600)	3 (0.600)
22	hsa-mir-3613	miRNA	0.067	158.000	10–245	2 (0.400)	2 (0.400)	4 (0.800)
23	hsa-mir-99a	miRNA	0.073	129.000	6–472	2 (0.400)	2 (0.400)	3 (0.600)
24	hsa-mir-5698	miRNA	0.065	114.000	14–719	2 (0.400)	2 (0.400)	4 (0.800)
25	hsa-mir-2115	miRNA	0.075	254.000	2–557	2 (0.400)	2 (0.400)	2 (0.400)
26	MAPT-AS1	mRNA	0.063	155.000	24–990	2 (0.400)	2 (0.400)	4 (0.800)
27	hsa-mir-3934	miRNA	0.063	322.000	20–759	2 (0.400)	2 (0.400)	2 (0.400)
28	hsa-mir-376b	miRNA	0.057	280.000	25–821	2 (0.400)	2 (0.400)	2 (0.400)
29	hsa-mir-15b	miRNA	0.066	251.000	5–1294	2 (0.400)	2 (0.400)	2 (0.400)
30	PYY	mRNA	0.054	264.000	28–1163	2 (0.400)	2 (0.400)	2 (0.400)
31	hsa-mir-4639	miRNA	0.064	189.000	15–1375	2 (0.400)	2 (0.400)	3 (0.600)
32	hsa-mir-147b	miRNA	0.052	669.000	30–985	2 (0.400)	2 (0.400)	2 (0.400)
33	PTTG1	mRNA	0.055	417.000	9–1236	2 (0.400)	2 (0.400)	2 (0.400)

Stability rank	Biomarker	Omics	Mean |IG|	Median rank	Rank range	Top 50, n (frequency)	Top 100, n (frequency)	Top 200, n (frequency)
34	hsa-mir-3177	miRNA	0.055	578.000	29–1501	2 (0.400)	2 (0.400)	2 (0.400)
35	RP11-774O3.3	mRNA	0.066	95.000	25–762	1 (0.200)	4 (0.800)	4 (0.800)
36	hsa-mir-4707	miRNA	0.066	85.000	30–155	1 (0.200)	3 (0.600)	5 (1.000)
37	hsa-mir-598	miRNA	0.063	70.000	20–445	1 (0.200)	3 (0.600)	3 (0.600)
38	hsa-mir-5691	miRNA	0.066	98.000	39–601	1 (0.200)	3 (0.600)	4 (0.800)
39	hsa-mir-3928	miRNA	0.066	94.000	13–855	1 (0.200)	3 (0.600)	4 (0.800)
40	ELOVL2-AS1	mRNA	0.059	100.000	11–937	1 (0.200)	3 (0.600)	3 (0.600)
41	RP11-1136G11.8	mRNA	0.055	92.000	26–1231	1 (0.200)	3 (0.600)	3 (0.600)
42	hsa-mir-30c-2	miRNA	0.065	112.000	36–315	1 (0.200)	2 (0.400)	4 (0.800)
43	hsa-mir-4640	miRNA	0.065	117.000	20–311	1 (0.200)	2 (0.400)	3 (0.600)
44	hsa-mir-5581	miRNA	0.061	224.000	50–275	1 (0.200)	2 (0.400)	2 (0.400)
45	hsa-mir-5187	miRNA	0.060	171.000	48–409	1 (0.200)	2 (0.400)	3 (0.600)
46	SCEL	mRNA	0.065	134.000	7–806	1 (0.200)	2 (0.400)	4 (0.800)
47	hsa-mir-4449	miRNA	0.061	119.000	43–799	1 (0.200)	2 (0.400)	4 (0.800)
48	hsa-mir-130b	miRNA	0.068	285.000	4–460	1 (0.200)	2 (0.400)	2 (0.400)
49	hsa-mir-92b	miRNA	0.059	351.000	26–463	1 (0.200)	2 (0.400)	2 (0.400)
50	hsa-mir-6882	miRNA	0.061	104.000	10–583	1 (0.200)	2 (0.400)	3 (0.600)

Stability rank	Biomarker	Omics	Mean |IG|	Median rank	Rank range	Top 50, n (frequency)	Top 100, n (frequency)	Top 200, n (frequency)
51	hsa-mir-3614	miRNA	0.056	265.000	21–582	1 (0.200)	2 (0.400)	2 (0.400)
52	SKA1	mRNA	0.058	256.000	44–761	1 (0.200)	2 (0.400)	2 (0.400)
53	hsa-mir-4802	miRNA	0.059	119.000	38–882	1 (0.200)	2 (0.400)	3 (0.600)
54	hsa-mir-509-3	miRNA	0.059	114.000	40–655	1 (0.200)	2 (0.400)	3 (0.600)
55	ATP13A5	mRNA	0.058	126.000	14–979	1 (0.200)	2 (0.400)	3 (0.600)
56	hsa-mir-92a-2	miRNA	0.058	115.000	28–996	1 (0.200)	2 (0.400)	3 (0.600)
57	hsa-mir-6820	miRNA	0.057	347.000	21–770	1 (0.200)	2 (0.400)	2 (0.400)
58	AC079776.2	mRNA	0.054	264.000	41–965	1 (0.200)	2 (0.400)	2 (0.400)
59	hsa-mir-5092	miRNA	0.060	123.000	42–1027	1 (0.200)	2 (0.400)	3 (0.600)
60	hsa-mir-202	miRNA	0.055	225.000	47–1019	1 (0.200)	2 (0.400)	2 (0.400)
61	NDC80	mRNA	0.051	456.000	33–859	1 (0.200)	2 (0.400)	2 (0.400)
62	hsa-mir-125b-2	miRNA	0.056	375.000	42–1221	1 (0.200)	2 (0.400)	2 (0.400)
63	hsa-mir-101-1	miRNA	0.057	137.000	22–1151	1 (0.200)	2 (0.400)	3 (0.600)
64	hsa-mir-3199-2	miRNA	0.058	261.000	31–968	1 (0.200)	2 (0.400)	2 (0.400)
65	hsa-mir-203b	miRNA	0.053	304.000	29–1110	1 (0.200)	2 (0.400)	2 (0.400)
66	hsa-mir-6875	miRNA	0.050	318.000	32–1005	1 (0.200)	2 (0.400)	2 (0.400)
67	hsa-mir-329-2	miRNA	0.051	546.000	42–1035	1 (0.200)	2 (0.400)	2 (0.400)

Stability rank	Biomarker	Omics	Mean |IG|	Median rank	Rank range	Top 50, n (frequency)	Top 100, n (frequency)	Top 200, n (frequency)
68	hsa-mir-33b	miRNA	0.050	488.000	34–1087	1 (0.200)	2 (0.400)	2 (0.400)
69	CTC-558O2.2	mRNA	0.051	517.000	41–1154	1 (0.200)	2 (0.400)	2 (0.400)
70	SMC1B	mRNA	0.054	539.000	24–1278	1 (0.200)	2 (0.400)	2 (0.400)
71	hsa-mir-22	miRNA	0.062	237.000	4–1448	1 (0.200)	2 (0.400)	2 (0.400)
72	hsa-mir-1307	miRNA	0.063	195.000	11–435	1 (0.200)	1 (0.200)	3 (0.600)
73	MAG	mRNA	0.057	234.000	18–431	1 (0.200)	1 (0.200)	2 (0.400)
74	hsa-mir-1269a	miRNA	0.064	185.000	12–579	1 (0.200)	1 (0.200)	3 (0.600)
75	hsa-mir-103b-1	miRNA	0.061	170.000	23–578	1 (0.200)	1 (0.200)	3 (0.600)
76	hsa-mir-6793	miRNA	0.060	176.000	28–696	1 (0.200)	1 (0.200)	3 (0.600)
77	RHCG	mRNA	0.057	245.000	25–649	1 (0.200)	1 (0.200)	2 (0.400)
78	GTSE1	mRNA	0.054	286.000	39–460	1 (0.200)	1 (0.200)	2 (0.400)
79	BMPR1B	mRNA	0.062	215.000	8–663	1 (0.200)	1 (0.200)	2 (0.400)
80	hsa-mir-3682	miRNA	0.058	206.000	38–598	1 (0.200)	1 (0.200)	2 (0.400)
81	hsa-mir-6716	miRNA	0.060	341.000	16–635	1 (0.200)	1 (0.200)	2 (0.400)
82	hsa-mir-153-2	miRNA	0.059	162.000	37–779	1 (0.200)	1 (0.200)	3 (0.600)
83	hsa-mir-329-1	miRNA	0.057	164.000	43–724	1 (0.200)	1 (0.200)	3 (0.600)
84	hsa-mir-4424	miRNA	0.056	286.000	24–710	1 (0.200)	1 (0.200)	2 (0.400)

Stability rank	Biomarker	Omics	Mean |IG|	Median rank	Rank range	Top 50, n (frequency)	Top 100, n (frequency)	Top 200, n (frequency)
85	hsa-mir-1284	miRNA	0.056	232.000	32–672	1 (0.200)	1 (0.200)	2 (0.400)
86	hsa-mir-375	miRNA	0.062	274.000	6–848	1 (0.200)	1 (0.200)	2 (0.400)
87	hsa-mir-6503	miRNA	0.059	209.000	18–979	1 (0.200)	1 (0.200)	2 (0.400)
88	CENPA	mRNA	0.057	360.000	38–714	1 (0.200)	1 (0.200)	2 (0.400)
89	hsa-mir-1228	miRNA	0.062	438.000	6–732	1 (0.200)	1 (0.200)	2 (0.400)
90	hsa-mir-4762	miRNA	0.054	221.000	50–820	1 (0.200)	1 (0.200)	1 (0.200)
91	SERPINA6	mRNA	0.055	320.000	33–697	1 (0.200)	1 (0.200)	2 (0.400)
92	hsa-mir-6516	miRNA	0.058	372.000	3–891	1 (0.200)	1 (0.200)	2 (0.400)
93	hsa-mir-3158-1	miRNA	0.054	393.000	45–609	1 (0.200)	1 (0.200)	1 (0.200)
94	ANLN	mRNA	0.050	363.000	32–594	1 (0.200)	1 (0.200)	1 (0.200)
95	hsa-mir-125a	miRNA	0.058	267.000	14–1073	1 (0.200)	1 (0.200)	2 (0.400)
96	hsa-mir-509-2	miRNA	0.057	248.000	27–1099	1 (0.200)	1 (0.200)	2 (0.400)
97	CCDC170	mRNA	0.052	536.000	36–634	1 (0.200)	1 (0.200)	2 (0.400)
98	hsa-mir-6510	miRNA	0.059	337.000	9–707	1 (0.200)	1 (0.200)	1 (0.200)
99	hsa-mir-5003	miRNA	0.065	349.000	1–817	1 (0.200)	1 (0.200)	1 (0.200)
100	CYP21A2	mRNA	0.054	488.000	37–796	1 (0.200)	1 (0.200)	2 (0.400)

Panel D. STAD

Stability rank	Biomarker	Omics	Mean |IG|	Median rank	Rank range	Top 50, n (frequency)	Top 100, n (frequency)	Top 200, n (frequency)
1	hsa-mir-181a-1	miRNA	0.103	37.000	1–316	3 (0.600)	4 (0.800)	4 (0.800)
2	BCL2L10	mRNA	0.101	36.000	1–682	3 (0.600)	4 (0.800)	4 (0.800)
3	AC226118.1	mRNA	0.102	47.000	1–209	3 (0.600)	3 (0.600)	4 (0.800)
4	RNF152	mRNA	0.094	34.000	5–468	3 (0.600)	3 (0.600)	3 (0.600)
5	MAGOH2P	mRNA	0.090	38.000	21–993	3 (0.600)	3 (0.600)	4 (0.800)
6	AC013275.2	mRNA	0.096	9.000	4–1387	3 (0.600)	3 (0.600)	4 (0.800)
7	GPA33	mRNA	0.086	36.000	18–895	3 (0.600)	3 (0.600)	3 (0.600)
8	CCND2	mRNA	0.085	38.000	7–1307	3 (0.600)	3 (0.600)	3 (0.600)
9	hsa-mir-4652	miRNA	0.092	82.000	5–496	2 (0.400)	3 (0.600)	4 (0.800)
10	RP11-260M19.2	mRNA	0.091	78.000	5–445	2 (0.400)	3 (0.600)	3 (0.600)
11	hsa-mir-452	miRNA	0.084	54.000	24–629	2 (0.400)	3 (0.600)	3 (0.600)
12	NINL	mRNA	0.089	83.000	6–983	2 (0.400)	3 (0.600)	4 (0.800)
13	VIPR1-AS1	mRNA	0.086	100.000	4–1229	2 (0.400)	3 (0.600)	3 (0.600)
14	MLH1	mRNA	0.094	129.000	7–208	2 (0.400)	2 (0.400)	4 (0.800)
15	ZP3	mRNA	0.086	168.000	9–539	2 (0.400)	2 (0.400)	3 (0.600)
16	ZIC5	mRNA	0.081	200.000	27–528	2 (0.400)	2 (0.400)	3 (0.600)

Stability rank	Biomarker	Omics	Mean |IG|	Median rank	Rank range	Top 50, n (frequency)	Top 100, n (frequency)	Top 200, n (frequency)
17	LINC01006	mRNA	0.086	134.000	7–534	2 (0.400)	2 (0.400)	3 (0.600)
18	CCDC129	mRNA	0.091	165.000	1–697	2 (0.400)	2 (0.400)	3 (0.600)
19	PCDHB11	mRNA	0.084	109.000	13–826	2 (0.400)	2 (0.400)	3 (0.600)
20	ADAMTSL5	mRNA	0.085	135.000	14–719	2 (0.400)	2 (0.400)	3 (0.600)
21	SLC25A23	mRNA	0.084	221.000	11–941	2 (0.400)	2 (0.400)	2 (0.400)
22	THAP5	mRNA	0.081	186.000	25–776	2 (0.400)	2 (0.400)	3 (0.600)
23	ABCG8	mRNA	0.082	402.000	3–645	2 (0.400)	2 (0.400)	2 (0.400)
24	SLC22A3	mRNA	0.080	155.000	19–857	2 (0.400)	2 (0.400)	3 (0.600)
25	VWA5A	mRNA	0.088	113.000	4–1262	2 (0.400)	2 (0.400)	3 (0.600)
26	FAH	mRNA	0.079	256.000	12–1237	2 (0.400)	2 (0.400)	2 (0.400)
27	CCL5	mRNA	0.088	268.000	5–1369	2 (0.400)	2 (0.400)	2 (0.400)
28	KLF7	mRNA	0.078	219.000	22–1298	2 (0.400)	2 (0.400)	2 (0.400)
29	ABLIM3	mRNA	0.080	139.000	13–1466	2 (0.400)	2 (0.400)	3 (0.600)
30	RP4-594A5.1	mRNA	0.076	264.000	14–1476	2 (0.400)	2 (0.400)	2 (0.400)
31	METTL20	mRNA	0.074	133.000	35–1488	2 (0.400)	2 (0.400)	3 (0.600)
32	hsa-let-7c	miRNA	0.076	452.000	8–1565	2 (0.400)	2 (0.400)	2 (0.400)
33	FASLG	mRNA	0.072	326.000	20–1608	2 (0.400)	2 (0.400)	2 (0.400)

Stability rank	Biomarker	Omics	Mean |IG|	Median rank	Rank range	Top 50, n (frequency)	Top 100, n (frequency)	Top 200, n (frequency)
34	hsa-mir-4741	miRNA	0.070	1163.000	27–1207	2 (0.400)	2 (0.400)	2 (0.400)
35	GUCY2C	mRNA	0.089	75.000	17–816	1 (0.200)	4 (0.800)	4 (0.800)
36	MLLT4-AS1	mRNA	0.087	73.000	11–418	1 (0.200)	3 (0.600)	3 (0.600)
37	hsa-mir-552	miRNA	0.078	67.000	26–1025	1 (0.200)	3 (0.600)	3 (0.600)
38	hsa-mir-3662	miRNA	0.074	82.000	30–1424	1 (0.200)	3 (0.600)	3 (0.600)
39	EMX1	mRNA	0.085	148.000	22–308	1 (0.200)	2 (0.400)	4 (0.800)
40	RP11-284F21.10	mRNA	0.084	125.000	18–328	1 (0.200)	2 (0.400)	3 (0.600)
41	HABP2	mRNA	0.085	156.000	13–418	1 (0.200)	2 (0.400)	4 (0.800)
42	hsa-mir-7846	miRNA	0.083	162.000	34–507	1 (0.200)	2 (0.400)	4 (0.800)
43	FAM3B	mRNA	0.082	207.000	15–432	1 (0.200)	2 (0.400)	2 (0.400)
44	ESPN	mRNA	0.081	254.000	30–592	1 (0.200)	2 (0.400)	2 (0.400)
45	ZNF683	mRNA	0.081	132.000	50–1013	1 (0.200)	2 (0.400)	4 (0.800)
46	RP11-369K16.1	mRNA	0.077	340.000	35–698	1 (0.200)	2 (0.400)	2 (0.400)
47	hsa-mir-181b-2	miRNA	0.075	377.000	44–650	1 (0.200)	2 (0.400)	2 (0.400)
48	hsa-mir-5589	miRNA	0.077	122.000	47–984	1 (0.200)	2 (0.400)	3 (0.600)
49	CDR1	mRNA	0.079	191.000	18–781	1 (0.200)	2 (0.400)	3 (0.600)
50	PROC	mRNA	0.080	131.000	21–890	1 (0.200)	2 (0.400)	3 (0.600)

Stability rank	Biomarker	Omics	Mean |IG|	Median rank	Rank range	Top 50, n (frequency)	Top 100, n (frequency)	Top 200, n (frequency)
51	ULBP3	mRNA	0.080	119.000	25–1210	1 (0.200)	2 (0.400)	3 (0.600)
52	SPATA18	mRNA	0.078	123.000	32–1220	1 (0.200)	2 (0.400)	3 (0.600)
53	hsa-mir-3199-1	miRNA	0.077	146.000	21–1029	1 (0.200)	2 (0.400)	3 (0.600)
54	PNPLA2	mRNA	0.073	415.000	37–735	1 (0.200)	2 (0.400)	2 (0.400)
55	WNT11	mRNA	0.077	142.000	19–1258	1 (0.200)	2 (0.400)	3 (0.600)
56	ANO7	mRNA	0.074	172.000	44–1485	1 (0.200)	2 (0.400)	3 (0.600)
57	RP11-44F21.5	mRNA	0.081	237.000	2–1002	1 (0.200)	2 (0.400)	2 (0.400)
58	hsa-mir-766	miRNA	0.076	293.000	10–1459	1 (0.200)	2 (0.400)	2 (0.400)
59	CAMK2N1	mRNA	0.071	360.000	43–1092	1 (0.200)	2 (0.400)	2 (0.400)
60	hsa-mir-25	miRNA	0.078	790.000	1–963	1 (0.200)	2 (0.400)	2 (0.400)
61	hsa-mir-96	miRNA	0.069	234.000	39–1633	1 (0.200)	2 (0.400)	2 (0.400)
62	hsa-mir-190a	miRNA	0.076	800.000	2–1124	1 (0.200)	2 (0.400)	2 (0.400)
63	CTD-2385L22.1	mRNA	0.072	420.000	23–1397	1 (0.200)	2 (0.400)	2 (0.400)
64	HOXA6	mRNA	0.070	351.000	43–1398	1 (0.200)	2 (0.400)	2 (0.400)
65	BTC	mRNA	0.075	992.000	2–1179	1 (0.200)	2 (0.400)	2 (0.400)
66	hsa-mir-6506	miRNA	0.069	346.000	13–1673	1 (0.200)	2 (0.400)	2 (0.400)
67	AC009014.3	mRNA	0.079	221.000	23–372	1 (0.200)	1 (0.200)	2 (0.400)

Stability rank	Biomarker	Omics	Mean |IG|	Median rank	Rank range	Top 50, n (frequency)	Top 100, n (frequency)	Top 200, n (frequency)
68	CXCL10	mRNA	0.082	174.000	12–418	1 (0.200)	1 (0.200)	3 (0.600)
69	C14orf105	mRNA	0.078	304.000	24–532	1 (0.200)	1 (0.200)	2 (0.400)
70	RP11-145A3.1	mRNA	0.081	181.000	10–714	1 (0.200)	1 (0.200)	3 (0.600)
71	FBXO25	mRNA	0.077	273.000	38–521	1 (0.200)	1 (0.200)	2 (0.400)
72	SULT4A1	mRNA	0.077	251.000	33–544	1 (0.200)	1 (0.200)	2 (0.400)
73	ADRA2A	mRNA	0.083	169.000	2–759	1 (0.200)	1 (0.200)	3 (0.600)
74	RP11-115D19.1	mRNA	0.076	317.000	42–547	1 (0.200)	1 (0.200)	2 (0.400)
75	CRABP2	mRNA	0.079	272.000	8–557	1 (0.200)	1 (0.200)	1 (0.200)
76	CXCL9	mRNA	0.077	301.000	23–573	1 (0.200)	1 (0.200)	1 (0.200)
77	FAM174A	mRNA	0.081	238.000	3–750	1 (0.200)	1 (0.200)	1 (0.200)
78	XXbac-B135H6.15	mRNA	0.077	331.000	24–540	1 (0.200)	1 (0.200)	2 (0.400)
79	hsa-mir-1185-2	miRNA	0.074	300.000	21–591	1 (0.200)	1 (0.200)	1 (0.200)
80	LEFTY1	mRNA	0.076	283.000	40–723	1 (0.200)	1 (0.200)	2 (0.400)
81	CDX2	mRNA	0.075	365.000	26–616	1 (0.200)	1 (0.200)	1 (0.200)
82	hsa-mir-181a-2	miRNA	0.073	285.000	48–772	1 (0.200)	1 (0.200)	2 (0.400)
83	TTLL2	mRNA	0.075	196.000	45–721	1 (0.200)	1 (0.200)	3 (0.600)
84	NXPE2	mRNA	0.075	391.000	16–846	1 (0.200)	1 (0.200)	2 (0.400)

Stability rank	Biomarker	Omics	Mean |IG|	Median rank	Rank range	Top 50, n (frequency)	Top 100, n (frequency)	Top 200, n (frequency)
85	hsa-mir-146a	miRNA	0.078	222.000	9–986	1 (0.200)	1 (0.200)	2 (0.400)
86	MUC6	mRNA	0.077	367.000	11–714	1 (0.200)	1 (0.200)	2 (0.400)
87	LINC00475	mRNA	0.074	296.000	36–858	1 (0.200)	1 (0.200)	2 (0.400)
88	hsa-mir-937	miRNA	0.071	262.000	38–760	1 (0.200)	1 (0.200)	1 (0.200)
89	ESPNP	mRNA	0.074	311.000	27–1102	1 (0.200)	1 (0.200)	2 (0.400)
90	hsa-mir-1258	miRNA	0.077	173.000	9–1240	1 (0.200)	1 (0.200)	3 (0.600)
91	GAS6-AS1	mRNA	0.074	392.000	11–793	1 (0.200)	1 (0.200)	1 (0.200)
92	OPN1SW	mRNA	0.075	466.000	15–788	1 (0.200)	1 (0.200)	2 (0.400)
93	CD55	mRNA	0.072	540.000	18–754	1 (0.200)	1 (0.200)	2 (0.400)
94	hsa-mir-145	miRNA	0.074	209.000	48–1161	1 (0.200)	1 (0.200)	2 (0.400)
95	MGC50722	mRNA	0.074	454.000	20–1132	1 (0.200)	1 (0.200)	2 (0.400)
96	PARM1	mRNA	0.073	388.000	15–1007	1 (0.200)	1 (0.200)	1 (0.200)
97	TMEM43	mRNA	0.077	258.000	7–1008	1 (0.200)	1 (0.200)	2 (0.400)
98	ANKRD16	mRNA	0.071	407.000	35–813	1 (0.200)	1 (0.200)	1 (0.200)
99	ZADH2	mRNA	0.075	183.000	10–1187	1 (0.200)	1 (0.200)	3 (0.600)
100	RP11-834C11.4	mRNA	0.073	300.000	25–1199	1 (0.200)	1 (0.200)	2 (0.400)

Note: Panels A–D correspond to the ROSMAP, SCZ, BRCA, and STAD datasets, respectively. Biomarker importance was quantified using the absolute integrated-gradients attribution score and summarized across five independent repeats. Mean |IG| represents the mean absolute attribution score across repeats. Median rank and rank range represent the median and minimum–maximum positions, respectively, of each biomarker in the feature-importance rankings across the five repeats. For each Top-k threshold, n denotes the number of repeats in which the biomarker was ranked among the top k features, and the corresponding proportion of the five repeats is shown in parentheses. Higher mean |IG| values, lower median ranks, narrower rank ranges, and higher Top-k frequencies indicate greater biomarker importance and cross-repeat stability. IG, integrated gradients; |IG|, absolute integrated-gradients attribution; BRCA, breast invasive carcinoma; STAD, stomach adenocarcinoma; ROSMAP, Religious Orders Study and Memory and Aging Project; SCZ, schizophrenia.

Table S5 Overall stability assessment of IG-derived biomarker rankings across five independent repeats

dataset	Number of repeat pairs	Spearman correlation (mean)	Spearman correlation (SD)	Top-50 Jaccard similarity	Top-100 Jaccard similarity	Top-200 Jaccard similarity	Number of features evaluated
BRCA	10	0.466	0.027	0.090	0.104	0.149	2000
STAD	10	0.474	0.028	0.053	0.070	0.111	2000
ROSMAP	10	0.142	0.081	0.106	0.160	0.246	600
SCZ	10	0.384	0.046	0.112	0.196	0.455	400

Note: Stability was assessed across five independent repeats using pairwise Spearman rank correlation of IG importance scores and Jaccard similarity of top-ranked feature sets. Values are presented as mean ± SD across all repeat pairs. IG, integrated gradients.

Table S6 Features selected by DIABLO across five independent repeats

dataset	repeat	block	component	feature
BRCA	1	mRNA	1	MLPH
	1	mRNA	1	FOXA1
	1	mRNA	1	TTC6
	1	mRNA	1	AGR3
	1	mRNA	1	LINC00504
	1	mRNA	1	CT62
	1	mRNA	1	AGR2
	1	mRNA	1	CTA-292E10.9
	1	mRNA	1	ESR1
	1	mRNA	1	CA12
	1	mRNA	1	SLC44A4
	1	mRNA	1	GATA3
	1	mRNA	1	DEGS2
	1	mRNA	1	PRR15
	1	mRNA	1	TBC1D9
	1	mRNA	1	TFF3
	1	mRNA	1	AR
	1	mRNA	1	SPDEF
	1	mRNA	1	C5AR2
	1	mRNA	1	C1orf64
	1	mRNA	1	ERBB4
	1	mRNA	1	CCDC170
	1	mRNA	1	THSD4
	1	mRNA	1	DNALI1
	1	mRNA	1	AC007255.8
	1	mRNA	1	ART3
	1	mRNA	1	AC005077.14
	1	mRNA	1	RP11-503N18.1
	1	mRNA	1	RP11-321G12.1
	1	mRNA	1	TFF1
	1	mRNA	2	AURKA
	1	mRNA	2	RRM2
	1	mRNA	2	CDKN3
	1	mRNA	2	CDC6
	1	mRNA	2	UBE2C
	1	mRNA	2	SGOL1
	1	mRNA	2	NCAPG
	1	mRNA	2	MYBL2
	1	mRNA	2	BIRC5
	1	mRNA	3	LINC01105
	1	mRNA	3	PMAIP1
	1	mRNA	3	ASB13

1	mRNA	3	SERPINA3
1	mRNA	3	NUDT6
1	mRNA	3	DBNDD2
1	mRNA	3	RP11-17M16.2
1	mRNA	3	NPY1R
1	mRNA	3	MPHOSPH6
1	mRNA	3	PPM1J
1	mRNA	3	MFSD2A
1	mRNA	3	NUDT8
1	mRNA	3	IGF1R
1	mRNA	3	C1orf226
1	mRNA	3	RP11-93K22.6
1	mRNA	3	SIAH2
1	mRNA	3	ZNF516
1	mRNA	3	ATP13A5
1	mRNA	3	PRRT2
1	mRNA	3	RP11-299G20.5
1	mRNA	3	CELSR2
1	mRNA	3	RP11-166N6.1
1	mRNA	3	NAGS
1	mRNA	3	RP11-299G20.2
1	mRNA	3	RERG
1	mRNA	3	PCSK6
1	mRNA	3	RBM24
1	mRNA	3	SIAH2-AS1
1	mRNA	3	RLN2
1	mRNA	3	TRPS1
1	miRNA	1	hsa-mir-934
1	miRNA	1	hsa-mir-577
1	miRNA	1	hsa-mir-18a
1	miRNA	1	hsa-mir-190b
1	miRNA	1	hsa-mir-135b
1	miRNA	1	hsa-mir-17
1	miRNA	1	hsa-mir-505
1	miRNA	1	hsa-mir-106b
1	miRNA	1	hsa-mir-584
1	miRNA	1	hsa-mir-519a-1
1	miRNA	1	hsa-mir-532
1	miRNA	1	hsa-mir-224
1	miRNA	1	hsa-mir-942
1	miRNA	1	hsa-mir-92a-1
1	miRNA	2	hsa-mir-454
1	miRNA	2	hsa-mir-3677
1	miRNA	2	hsa-mir-1301

1	miRNA	2	hsa-mir-4664
1	miRNA	2	hsa-mir-301a
1	miRNA	3	hsa-mir-2115
1	miRNA	3	hsa-mir-4728
1	miRNA	3	hsa-mir-7158
1	miRNA	3	hsa-mir-30a
1	miRNA	3	hsa-mir-3150b
1	miRNA	3	hsa-mir-187
1	miRNA	3	hsa-mir-4446
1	miRNA	3	hsa-mir-30c-2
1	miRNA	3	hsa-mir-6715a
2	mRNA	1	MLPH
2	mRNA	1	FOXA1
2	mRNA	1	TTC6
2	mRNA	1	AGR3
2	mRNA	1	LINC00504
2	mRNA	1	CT62
2	mRNA	1	CTA-292E10.9
2	mRNA	1	ESR1
2	mRNA	1	AGR2
2	mRNA	1	DEGS2
2	mRNA	1	CA12
2	mRNA	1	SLC44A4
2	mRNA	1	PRR15
2	mRNA	1	GATA3
2	mRNA	1	AR
2	mRNA	1	TBC1D9
2	mRNA	1	TFF3
2	mRNA	1	ERBB4
2	mRNA	1	AC005077.14
2	mRNA	1	C1orf64
2	mRNA	1	SPDEF
2	mRNA	1	C5AR2
2	mRNA	1	THSD4
2	mRNA	1	RP11-503N18.1
2	mRNA	1	TFF1
2	mRNA	1	CCDC170
2	mRNA	1	AC007255.8
2	mRNA	1	RP11-321G12.1
2	mRNA	1	KCNJ11
2	mRNA	1	AC008268.1
2	mRNA	2	AURKA
2	mRNA	2	CDC6
2	mRNA	2	RRM2

2	mRNA	2	CDKN3
2	mRNA	2	NCAPG
2	mRNA	2	SGOL1
2	mRNA	2	NCAPH
2	mRNA	2	UBE2C
2	mRNA	2	TROAP
2	mRNA	2	SHCBP1
2	mRNA	3	LINC01105
2	mRNA	3	PMAIP1
2	mRNA	3	ASB13
2	mRNA	3	DBNDD2
2	mRNA	3	SERPINA3
2	mRNA	3	NUDT6
2	mRNA	3	MPHOSPH6
2	mRNA	3	PPM1J
2	mRNA	3	ATP13A5
2	mRNA	3	NUDT8
2	mRNA	3	NPY1R
2	mRNA	3	RP11-93K22.6
2	mRNA	3	MFSD2A
2	mRNA	3	IGF1R
2	mRNA	3	SIAH2
2	mRNA	3	PRRT2
2	miRNA	1	hsa-mir-934
2	miRNA	1	hsa-mir-18a
2	miRNA	1	hsa-mir-577
2	miRNA	1	hsa-mir-135b
2	miRNA	1	hsa-mir-190b
2	miRNA	1	hsa-mir-17
2	miRNA	1	hsa-mir-505
2	miRNA	1	hsa-mir-106b
2	miRNA	1	hsa-mir-584
2	miRNA	1	hsa-mir-942
2	miRNA	1	hsa-mir-92a-1
2	miRNA	1	hsa-mir-92a-2
2	miRNA	1	hsa-mir-19a
2	miRNA	1	hsa-mir-532
2	miRNA	1	hsa-mir-590
2	miRNA	1	hsa-mir-130b
2	miRNA	1	hsa-mir-877
2	miRNA	1	hsa-mir-197
2	miRNA	1	hsa-mir-20a
2	miRNA	1	hsa-mir-519a-1
2	miRNA	1	hsa-mir-378a

2	miRNA	1	hsa-mir-455
2	miRNA	1	hsa-mir-188
2	miRNA	1	hsa-mir-19b-1
2	miRNA	1	hsa-mir-502
2	miRNA	1	hsa-mir-224
2	miRNA	1	hsa-mir-9-3
2	miRNA	1	hsa-mir-9-2
2	miRNA	1	hsa-mir-9-1
2	miRNA	1	hsa-mir-500b
2	miRNA	2	hsa-mir-454
2	miRNA	2	hsa-mir-3677
2	miRNA	2	hsa-let-7c
2	miRNA	2	hsa-mir-4664
2	miRNA	2	hsa-mir-4756
2	miRNA	3	hsa-mir-4728
2	miRNA	3	hsa-mir-7158
2	miRNA	3	hsa-mir-2115
2	miRNA	3	hsa-mir-6715a
2	miRNA	3	hsa-mir-30a
2	miRNA	3	hsa-mir-3150b
2	miRNA	3	hsa-mir-187
2	miRNA	3	hsa-mir-30c-2
2	miRNA	3	hsa-mir-4446
2	miRNA	3	hsa-mir-5685
2	miRNA	3	hsa-mir-342
2	miRNA	3	hsa-mir-30c-1
2	miRNA	3	hsa-mir-5691
2	miRNA	3	hsa-mir-148a
2	miRNA	3	hsa-mir-485
2	miRNA	3	hsa-mir-548t
2	miRNA	3	hsa-mir-4714
2	miRNA	3	hsa-mir-5698
2	miRNA	3	hsa-mir-889
2	miRNA	3	hsa-mir-190b
2	miRNA	3	hsa-mir-495
2	miRNA	3	hsa-mir-6509
2	miRNA	3	hsa-mir-3934
2	miRNA	3	hsa-mir-224
2	miRNA	3	hsa-mir-452
3	mRNA	1	MLPH
3	mRNA	1	FOXA1
3	mRNA	1	TTC6
3	mRNA	1	LINC00504
3	mRNA	1	AGR3

3	mRNA	1	CT62
3	mRNA	1	CTA-292E10.9
3	mRNA	1	CA12
3	mRNA	1	ESR1
3	mRNA	1	AGR2
3	mRNA	1	SLC44A4
3	mRNA	1	GATA3
3	mRNA	1	PRR15
3	mRNA	1	TBC1D9
3	mRNA	1	AR
3	mRNA	1	TFF3
3	mRNA	1	SPDEF
3	mRNA	1	DEGS2
3	mRNA	1	C1orf64
3	mRNA	1	ERBB4
3	mRNA	1	C5AR2
3	mRNA	1	AC005077.14
3	mRNA	1	CCDC170
3	mRNA	1	AC007255.8
3	mRNA	1	RP11-321G12.1
3	mRNA	1	TFF1
3	mRNA	1	THSD4
3	mRNA	1	DNALI1
3	mRNA	1	RP11-503N18.1
3	mRNA	1	ART3
3	mRNA	2	RRM2
3	mRNA	2	AURKA
3	mRNA	2	CDKN3
3	mRNA	2	CDC6
3	mRNA	2	NCAPG
3	mRNA	2	SGOL1
3	mRNA	2	SHCBP1
3	mRNA	2	NCAPH
3	mRNA	3	LINC01105
3	mRNA	3	MPHOSPH6
3	mRNA	3	PMAIP1
3	mRNA	3	ATP13A5
3	mRNA	3	NUDT8
3	mRNA	3	DBNDD2
3	mRNA	3	ASB13
3	mRNA	3	MFSD2A
3	mRNA	3	IGF1R
3	mRNA	3	RP11-93K22.6
3	mRNA	3	RP11-17M16.2

3	mRNA	3	RP3-443C4.2
3	mRNA	3	SERPINA3
3	mRNA	3	PRRT2
3	mRNA	3	HSD17B2
3	mRNA	3	NPY1R
3	mRNA	3	TRPV6
3	mRNA	3	NUDT6
3	mRNA	3	STX19
3	mRNA	3	SIAH2
3	mRNA	3	PPM1J
3	mRNA	3	RP11-28F1.2
3	mRNA	3	LPPR3
3	mRNA	3	ESR1
3	mRNA	3	RP11-166N6.1
3	miRNA	1	hsa-mir-934
3	miRNA	1	hsa-mir-577
3	miRNA	1	hsa-mir-18a
3	miRNA	1	hsa-mir-135b
3	miRNA	1	hsa-mir-190b
3	miRNA	1	hsa-mir-17
3	miRNA	1	hsa-mir-505
3	miRNA	1	hsa-mir-584
3	miRNA	1	hsa-mir-106b
3	miRNA	1	hsa-mir-92a-1
3	miRNA	1	hsa-mir-19a
3	miRNA	1	hsa-mir-92a-2
3	miRNA	1	hsa-mir-20a
3	miRNA	1	hsa-mir-532
3	miRNA	1	hsa-mir-942
3	miRNA	1	hsa-mir-519a-1
3	miRNA	1	hsa-mir-224
3	miRNA	1	hsa-mir-455
3	miRNA	1	hsa-mir-590
3	miRNA	1	hsa-mir-130b
3	miRNA	1	hsa-mir-19b-1
3	miRNA	1	hsa-mir-452
3	miRNA	1	hsa-mir-500a
3	miRNA	1	hsa-mir-500b
3	miRNA	1	hsa-mir-197
3	miRNA	1	hsa-mir-877
3	miRNA	1	hsa-mir-378a
3	miRNA	1	hsa-mir-9-3
3	miRNA	1	hsa-mir-9-1
3	miRNA	1	hsa-mir-9-2

3	miRNA	2	hsa-mir-4728
3	miRNA	2	hsa-mir-3677
3	miRNA	2	hsa-mir-454
3	miRNA	2	hsa-mir-4446
3	miRNA	2	hsa-mir-30a
3	miRNA	2	hsa-mir-423
3	miRNA	2	hsa-mir-210
3	miRNA	2	hsa-mir-1301
3	miRNA	2	hsa-mir-301a
3	miRNA	2	hsa-mir-4756
3	miRNA	3	hsa-mir-7158
3	miRNA	3	hsa-mir-2115
3	miRNA	3	hsa-mir-342
3	miRNA	3	hsa-mir-190b
3	miRNA	3	hsa-mir-4728
3	miRNA	3	hsa-mir-4714
3	miRNA	3	hsa-mir-889
3	miRNA	3	hsa-mir-99b
3	miRNA	3	hsa-mir-30b
3	miRNA	3	hsa-mir-769
3	miRNA	3	hsa-mir-6715a
3	miRNA	3	hsa-mir-410
3	miRNA	3	hsa-mir-125a
3	miRNA	3	hsa-mir-30c-2
3	miRNA	3	hsa-mir-339
3	miRNA	3	hsa-mir-149
3	miRNA	3	hsa-mir-6783
3	miRNA	3	hsa-mir-30c-1
3	miRNA	3	hsa-mir-187
3	miRNA	3	hsa-mir-5691
3	miRNA	3	hsa-mir-454
3	miRNA	3	hsa-mir-328
3	miRNA	3	hsa-mir-548t
3	miRNA	3	hsa-mir-16-2
3	miRNA	3	hsa-let-7e
3	miRNA	3	hsa-mir-452
3	miRNA	3	hsa-mir-16-1
3	miRNA	3	hsa-mir-381
3	miRNA	3	hsa-mir-98
3	miRNA	3	hsa-mir-380
4	mRNA	1	MLPH
4	mRNA	1	FOXA1
4	mRNA	1	TTC6
4	mRNA	1	LINC00504

4	mRNA	1	CT62
4	mRNA	1	AGR3
4	mRNA	1	CTA-292E10.9
4	mRNA	1	ESR1
4	mRNA	1	CA12
4	mRNA	1	AGR2
4	mRNA	1	SLC44A4
4	mRNA	1	PRR15
4	mRNA	1	GATA3
4	mRNA	1	TBC1D9
4	mRNA	1	TFF3
4	mRNA	1	SPDEF
4	mRNA	1	AR
4	mRNA	1	DEGS2
4	mRNA	1	C1orf64
4	mRNA	1	C5AR2
4	mRNA	1	AC005077.14
4	mRNA	1	AC007255.8
4	mRNA	1	CCDC170
4	mRNA	1	ERBB4
4	mRNA	1	THSD4
4	mRNA	2	AURKA
4	mRNA	2	RRM2
4	mRNA	2	CDKN3
4	mRNA	2	CDC6
4	mRNA	2	NCAPG
4	mRNA	2	SGOL1
4	mRNA	2	NCAPH
4	mRNA	2	SHCBP1
4	mRNA	2	BIRC5
4	mRNA	2	TPX2
4	mRNA	2	UBE2C
4	mRNA	2	MYBL2
4	mRNA	2	TROAP
4	mRNA	2	SKA1
4	mRNA	3	LINC01105
4	mRNA	3	PMAIP1
4	mRNA	3	ASB13
4	mRNA	3	DBNDD2
4	mRNA	3	NUDT6
4	mRNA	3	NUDT8
4	mRNA	3	MPHOSPH6
4	mRNA	3	RP11-17M16.2
4	mRNA	3	SERPINA3

4	mRNA	3	MFSD2A
4	mRNA	3	RP11-93K22.6
4	mRNA	3	C1orf226
4	mRNA	3	RERG
4	mRNA	3	NPY1R
4	mRNA	3	IGF1R
4	mRNA	3	SIAH2-AS1
4	mRNA	3	ATP13A5
4	mRNA	3	SIAH2
4	mRNA	3	PPM1J
4	mRNA	3	RP11-166N6.1
4	mRNA	3	ZNF516
4	mRNA	3	CELSR2
4	mRNA	3	BCL2
4	mRNA	3	TRPS1
4	mRNA	3	LPPR3
4	mRNA	3	S100A8
4	mRNA	3	NAGS
4	mRNA	3	S100A9
4	mRNA	3	RP11-28F1.2
4	mRNA	3	RP11-299G20.5
4	miRNA	1	hsa-mir-934
4	miRNA	1	hsa-mir-577
4	miRNA	1	hsa-mir-18a
4	miRNA	1	hsa-mir-190b
4	miRNA	1	hsa-mir-135b
4	miRNA	1	hsa-mir-17
4	miRNA	2	hsa-mir-454
4	miRNA	2	hsa-mir-3677
4	miRNA	2	hsa-mir-301a
4	miRNA	2	hsa-mir-4664
4	miRNA	2	hsa-mir-1301
4	miRNA	2	hsa-mir-130b
4	miRNA	2	hsa-mir-423
4	miRNA	2	hsa-mir-1307
4	miRNA	2	hsa-mir-210
4	miRNA	2	hsa-mir-3074
4	miRNA	3	hsa-mir-2115
4	miRNA	3	hsa-mir-7158
4	miRNA	3	hsa-mir-4728
4	miRNA	3	hsa-mir-30a
4	miRNA	3	hsa-mir-187
4	miRNA	3	hsa-mir-3150b
4	miRNA	3	hsa-mir-30c-2

4	miRNA	3	hsa-mir-6715a
5	mRNA	1	MLPH
5	mRNA	1	FOXA1
5	mRNA	1	TTC6
5	mRNA	1	LINC00504
5	mRNA	1	AGR3
5	mRNA	1	CT62
5	mRNA	1	ESR1
5	mRNA	1	CTA-292E10.9
5	mRNA	1	CA12
5	mRNA	1	AGR2
5	mRNA	1	GATA3
5	mRNA	1	SLC44A4
5	mRNA	1	DEGS2
5	mRNA	1	TBC1D9
5	mRNA	1	PRR15
5	mRNA	1	TFF3
5	mRNA	1	C1orf64
5	mRNA	1	C5AR2
5	mRNA	1	SPDEF
5	mRNA	1	AC005077.14
5	mRNA	1	AR
5	mRNA	1	DNALI1
5	mRNA	1	CCDC170
5	mRNA	1	THSD4
5	mRNA	1	ERBB4
5	mRNA	1	AC007255.8
5	mRNA	1	KCNJ11
5	mRNA	1	SLC7A8
5	mRNA	1	RP11-503N18.1
5	mRNA	1	TFF1
5	mRNA	2	AURKA
5	mRNA	2	CDKN3
5	mRNA	2	CDC6
5	mRNA	2	RRM2
5	mRNA	2	SGOL1
5	mRNA	2	NCAPG
5	mRNA	2	SHCBP1
5	mRNA	2	UBE2C
5	mRNA	2	FAM83D
5	mRNA	2	NCAPH
5	mRNA	3	PMAIP1
5	mRNA	3	LINC01105
5	mRNA	3	ASB13

5	mRNA	3	DBNDD2
5	mRNA	3	NUDT6
5	mRNA	3	RP11-17M16.2
5	mRNA	3	MPHOSPH6
5	mRNA	3	PPM1J
5	mRNA	3	SERPINA3
5	mRNA	3	NUDT8
5	mRNA	3	NPY1R
5	mRNA	3	ATP13A5
5	mRNA	3	RP11-93K22.6
5	mRNA	3	SIAH2
5	mRNA	3	ZNF516
5	mRNA	3	MFSD2A
5	mRNA	3	IGF1R
5	mRNA	3	C1orf226
5	mRNA	3	PRRT2
5	mRNA	3	RERG
5	mRNA	3	RP11-166N6.1
5	mRNA	3	SIAH2-AS1
5	mRNA	3	C18orf65
5	mRNA	3	NAGS
5	mRNA	3	BCL2
5	miRNA	1	hsa-mir-934
5	miRNA	1	hsa-mir-577
5	miRNA	1	hsa-mir-18a
5	miRNA	1	hsa-mir-190b
5	miRNA	1	hsa-mir-135b
5	miRNA	1	hsa-mir-17
5	miRNA	1	hsa-mir-505
5	miRNA	1	hsa-mir-106b
5	miRNA	1	hsa-mir-584
5	miRNA	1	hsa-mir-224
5	miRNA	1	hsa-mir-92a-1
5	miRNA	1	hsa-mir-942
5	miRNA	1	hsa-mir-92a-2
5	miRNA	1	hsa-mir-519a-1
5	miRNA	1	hsa-mir-452
5	miRNA	1	hsa-mir-19a
5	miRNA	1	hsa-mir-378a
5	miRNA	1	hsa-mir-20a
5	miRNA	1	hsa-mir-590
5	miRNA	1	hsa-mir-455
5	miRNA	1	hsa-mir-532
5	miRNA	1	hsa-mir-130b

	5	miRNA	1	hsa-mir-197
	5	miRNA	1	hsa-mir-188
	5	miRNA	1	hsa-mir-877
	5	miRNA	1	hsa-mir-19b-1
	5	miRNA	1	hsa-mir-9-3
	5	miRNA	1	hsa-mir-301b
	5	miRNA	1	hsa-mir-9-2
	5	miRNA	1	hsa-mir-9-1
	5	miRNA	2	hsa-mir-3677
	5	miRNA	2	hsa-mir-4664
	5	miRNA	2	hsa-mir-4446
	5	miRNA	2	hsa-mir-454
	5	miRNA	2	hsa-mir-301a
	5	miRNA	2	hsa-let-7c
	5	miRNA	2	hsa-mir-940
	5	miRNA	3	hsa-mir-4728
	5	miRNA	3	hsa-mir-7158
	5	miRNA	3	hsa-mir-2115
	5	miRNA	3	hsa-mir-30a
	5	miRNA	3	hsa-mir-30c-2
	5	miRNA	3	hsa-mir-187
	5	miRNA	3	hsa-mir-3150b
	5	miRNA	3	hsa-mir-6715a
	5	miRNA	3	hsa-mir-342
	5	miRNA	3	hsa-mir-4446
	5	miRNA	3	hsa-mir-30c-1
	5	miRNA	3	hsa-mir-125a
	5	miRNA	3	hsa-mir-5691
	5	miRNA	3	hsa-mir-4714
STAD	1	mRNA	1	C1QTNF2
	1	mRNA	1	PLAC9
	1	mRNA	1	C16orf45
	1	mRNA	1	FLNC
	1	mRNA	1	SSBP2
	1	mRNA	1	SYNC
	1	mRNA	1	ZNF385D
	1	mRNA	1	NFATC4
	1	mRNA	1	SRPX
	1	mRNA	1	SPON1
	1	mRNA	1	KIAA1644
	1	mRNA	1	GNAL
	1	mRNA	1	TUB
	1	mRNA	1	ZNF454
	1	mRNA	2	MLH1

1	mRNA	2	LINC01006
1	mRNA	2	MLLT4-AS1
1	mRNA	2	BCL2L10
1	mRNA	2	PROC
1	mRNA	2	NINL
1	mRNA	2	SPIN3
1	mRNA	2	AC013275.2
1	mRNA	2	CCDC129
1	mRNA	2	SYT7
1	miRNA	1	hsa-mir-141
1	miRNA	1	hsa-mir-200c
1	miRNA	1	hsa-mir-99a
1	miRNA	1	hsa-mir-1307
1	miRNA	1	hsa-mir-130b
1	miRNA	1	hsa-mir-106b
1	miRNA	1	hsa-let-7c
1	miRNA	1	hsa-mir-96
1	miRNA	1	hsa-mir-100
1	miRNA	1	hsa-mir-652
1	miRNA	1	hsa-mir-3926-2
1	miRNA	1	hsa-mir-182
1	miRNA	1	hsa-mir-616
1	miRNA	1	hsa-mir-1-1
1	miRNA	1	hsa-mir-576
1	miRNA	1	hsa-mir-455
1	miRNA	1	hsa-mir-7-1
1	miRNA	1	hsa-mir-1-2
1	miRNA	1	hsa-mir-222
1	miRNA	1	hsa-mir-93
1	miRNA	1	hsa-mir-200a
1	miRNA	1	hsa-mir-15b
1	miRNA	1	hsa-mir-133a-1
1	miRNA	1	hsa-mir-133a-2
1	miRNA	1	hsa-mir-3127
1	miRNA	1	hsa-mir-219a-1
1	miRNA	1	hsa-mir-589
1	miRNA	1	hsa-mir-197
1	miRNA	1	hsa-mir-148b
1	miRNA	1	hsa-mir-139
1	miRNA	2	hsa-mir-181b-2
1	miRNA	2	hsa-mir-181b-1
1	miRNA	2	hsa-mir-934
1	miRNA	2	hsa-mir-181d
1	miRNA	2	hsa-mir-4652

2	mRNA	1	C1QTNF2
2	mRNA	1	NFATC4
2	mRNA	1	C16orf45
2	mRNA	1	ZNF385D
2	mRNA	1	SPON1
2	mRNA	1	FLNC
2	mRNA	1	SYNC
2	mRNA	1	SSBP2
2	mRNA	1	SRPX
2	mRNA	1	PLAC9
2	mRNA	1	ZNF454
2	mRNA	1	KIAA1644
2	mRNA	1	NUDT10
2	mRNA	1	BMPER
2	mRNA	2	MLH1
2	mRNA	2	LINC01006
2	mRNA	2	MLLT4-AS1
2	mRNA	2	PROC
2	mRNA	2	NINL
2	mRNA	2	BCL2L10
2	mRNA	2	SPIN3
2	miRNA	1	hsa-mir-141
2	miRNA	1	hsa-mir-200c
2	miRNA	1	hsa-mir-99a
2	miRNA	1	hsa-let-7c
2	miRNA	1	hsa-mir-1307
2	miRNA	1	hsa-mir-106b
2	miRNA	1	hsa-mir-96
2	miRNA	1	hsa-mir-1-1
2	miRNA	1	hsa-mir-1-2
2	miRNA	1	hsa-mir-100
2	miRNA	1	hsa-mir-200a
2	miRNA	1	hsa-mir-652
2	miRNA	1	hsa-mir-3926-2
2	miRNA	1	hsa-mir-133a-2
2	miRNA	1	hsa-mir-200b
2	miRNA	1	hsa-mir-133a-1
2	miRNA	1	hsa-mir-93
2	miRNA	1	hsa-mir-130b
2	miRNA	1	hsa-mir-616
2	miRNA	1	hsa-mir-576
2	miRNA	1	hsa-mir-182
2	miRNA	1	hsa-mir-15b
2	miRNA	1	hsa-mir-17

2	miRNA	1	hsa-mir-589
2	miRNA	1	hsa-mir-222
2	miRNA	1	hsa-mir-7-1
2	miRNA	1	hsa-mir-429
2	miRNA	1	hsa-mir-139
2	miRNA	1	hsa-mir-148b
2	miRNA	1	hsa-mir-133b
2	miRNA	2	hsa-mir-181d
2	miRNA	2	hsa-mir-181b-2
2	miRNA	2	hsa-mir-181c
2	miRNA	2	hsa-mir-181b-1
2	miRNA	2	hsa-mir-4652
3	mRNA	1	C1QTNF2
3	mRNA	1	NFATC4
3	mRNA	1	SRPX
3	mRNA	1	ZNF385D
3	mRNA	1	FLNC
3	mRNA	1	KIAA1644
3	mRNA	1	SPON1
3	mRNA	1	NUDT10
3	mRNA	1	SYNC
3	mRNA	1	C16orf45
3	mRNA	1	SSBP2
3	mRNA	1	PLAC9
3	mRNA	1	BMPER
3	mRNA	1	MCC
3	mRNA	2	LINC01006
3	mRNA	2	MLH1
3	mRNA	2	MLLT4-AS1
3	mRNA	2	NINL
3	mRNA	2	PROC
3	mRNA	2	BCL2L10
3	mRNA	2	SYT7
3	mRNA	2	C2orf70
3	mRNA	2	SPIN3
3	mRNA	2	AC013275.2
3	mRNA	2	CCND2
3	mRNA	2	TMEM136
3	mRNA	2	CCDC129
3	mRNA	2	ERICH5
3	mRNA	2	RP11-218M22.1
3	mRNA	2	GPA33
3	mRNA	2	HEPH
3	mRNA	2	NEURL3

3	mRNA	2	HABP2
3	mRNA	2	CFAP46
3	mRNA	2	SLC25A23
3	mRNA	2	TMEM171
3	mRNA	2	FAM131C
3	mRNA	2	DEPDC7
3	mRNA	2	ITGAE
3	mRNA	2	SPATA18
3	mRNA	2	LEFTY1
3	mRNA	2	FAM213A
3	mRNA	2	TTLL2
3	mRNA	2	ZIC5
3	miRNA	1	hsa-mir-141
3	miRNA	1	hsa-mir-200c
3	miRNA	1	hsa-mir-1307
3	miRNA	1	hsa-mir-106b
3	miRNA	1	hsa-mir-652
3	miRNA	1	hsa-mir-96
3	miRNA	1	hsa-let-7c
3	miRNA	1	hsa-mir-616
3	miRNA	1	hsa-mir-99a
3	miRNA	1	hsa-mir-130b
3	miRNA	2	hsa-mir-181d
3	miRNA	2	hsa-mir-582
3	miRNA	2	hsa-mir-181b-2
3	miRNA	2	hsa-mir-934
3	miRNA	2	hsa-mir-4652
4	mRNA	1	C1QTNF2
4	mRNA	1	NFATC4
4	mRNA	1	ZNF385D
4	mRNA	1	SRPX
4	mRNA	1	C16orf45
4	mRNA	1	PLAC9
4	mRNA	1	TUB
4	mRNA	1	SSBP2
4	mRNA	1	MCC
4	mRNA	1	NUDT10
4	mRNA	1	SPON1
4	mRNA	1	PDZRN3
4	mRNA	1	TUBB6
4	mRNA	1	SYNC
4	mRNA	1	MAGI2
4	mRNA	1	FLNC
4	mRNA	1	DSG2

4	mRNA	1	ZNF454
4	mRNA	2	MLH1
4	mRNA	2	LINC01006
4	mRNA	2	MLLT4-AS1
4	mRNA	2	NINL
4	mRNA	2	BCL2L10
4	mRNA	2	PROC
4	mRNA	2	SPIN3
4	mRNA	2	CCND2
4	mRNA	2	SPATA18
4	miRNA	1	hsa-mir-99a
4	miRNA	1	hsa-mir-100
4	miRNA	1	hsa-let-7c
4	miRNA	1	hsa-mir-141
4	miRNA	1	hsa-mir-200c
4	miRNA	1	hsa-mir-96
4	miRNA	1	hsa-mir-3926-2
4	miRNA	1	hsa-mir-1307
4	miRNA	1	hsa-mir-576
4	miRNA	1	hsa-mir-130b
4	miRNA	1	hsa-mir-652
4	miRNA	1	hsa-mir-1-1
4	miRNA	1	hsa-mir-182
4	miRNA	1	hsa-mir-1-2
4	miRNA	1	hsa-mir-616
4	miRNA	1	hsa-mir-106b
4	miRNA	1	hsa-mir-3127
4	miRNA	1	hsa-mir-200b
4	miRNA	2	hsa-mir-181b-2
4	miRNA	2	hsa-mir-181b-1
4	miRNA	2	hsa-mir-181d
4	miRNA	2	hsa-mir-181a-2
4	miRNA	2	hsa-mir-181a-1
5	mRNA	1	NFATC4
5	mRNA	1	C1QTNF2
5	mRNA	1	ZNF385D
5	mRNA	1	TMEM52
5	mRNA	1	SRPX
5	mRNA	2	LINC01006
5	mRNA	2	MLLT4-AS1
5	mRNA	2	PROC
5	mRNA	2	NINL
5	mRNA	2	MLH1
5	mRNA	2	BCL2L10

	5	mRNA	2	C2orf70
	5	mRNA	2	CCND2
	5	mRNA	2	CCDC129
	5	mRNA	2	SPATA18
	5	mRNA	2	RP11-44F21.5
	5	mRNA	2	GPA33
	5	mRNA	2	FAM213A
	5	mRNA	2	HEPH
	5	mRNA	2	SLC12A2
	5	mRNA	2	SPIN3
	5	miRNA	1	hsa-mir-200c
	5	miRNA	1	hsa-mir-141
	5	miRNA	1	hsa-let-7c
	5	miRNA	1	hsa-mir-99a
	5	miRNA	1	hsa-mir-1307
	5	miRNA	1	hsa-mir-100
	5	miRNA	1	hsa-mir-96
	5	miRNA	1	hsa-mir-652
	5	miRNA	1	hsa-mir-3926-2
	5	miRNA	2	hsa-mir-181d
	5	miRNA	2	hsa-mir-181b-2
	5	miRNA	2	hsa-mir-181b-1
	5	miRNA	2	hsa-mir-4652
	5	miRNA	2	hsa-mir-934
	5	miRNA	2	hsa-mir-582
	5	miRNA	2	hsa-mir-181c
	5	miRNA	2	hsa-mir-181a-2
	5	miRNA	2	hsa-mir-155
	5	miRNA	2	hsa-mir-338
	5	miRNA	2	hsa-mir-181a-1
	5	miRNA	2	hsa-mir-937
	5	miRNA	2	hsa-mir-1275
	5	miRNA	2	hsa-mir-142
	5	miRNA	2	hsa-mir-766
	5	miRNA	2	hsa-mir-1245a
	5	miRNA	2	hsa-mir-147b
	5	miRNA	2	hsa-mir-361
	5	miRNA	2	hsa-mir-4733
	5	miRNA	2	hsa-mir-4658
ROSMAP	1	mRNA	1	ENSG00000196517.7
	1	mRNA	1	ENSG00000011426.6
	1	mRNA	1	ENSG00000111181.8
	1	mRNA	1	ENSG00000198624.8
	1	mRNA	1	ENSG00000088836.7

1	mRNA	1	ENSG00000105643.3
1	mRNA	1	ENSG00000196154.7
1	mRNA	1	ENSG00000125534.4
1	miRNA	1	hsa-miR-132
1	miRNA	1	hsa-miR-129-5p
1	miRNA	1	hsa-miR-29a
1	miRNA	1	hsa-miR-100
1	miRNA	1	hsa-miR-133b
1	miRNA	1	hsa-miR-362-3p
1	miRNA	1	ebv-miR-BART8
1	DNA methylation	1	cg12845808
1	DNA methylation	1	cg04983977
1	DNA methylation	1	cg15780361
1	DNA methylation	1	cg11052143
1	DNA methylation	1	cg00745735
1	DNA methylation	1	cg03670238
2	mRNA	1	ENSG00000196154.7
2	mRNA	1	ENSG00000111181.8
2	mRNA	1	ENSG00000198624.8
2	mRNA	1	ENSG00000196517.7
2	mRNA	1	ENSG00000011426.6
2	mRNA	1	ENSG00000105643.3
2	mRNA	1	ENSG00000188783.5
2	mRNA	1	ENSG00000125534.4
2	mRNA	1	ENSG00000088836.7
2	mRNA	1	ENSG00000170759.10
2	mRNA	1	ENSG00000021300.7
2	mRNA	1	ENSG00000171388.9
2	miRNA	1	hsa-miR-132
2	miRNA	1	hsa-miR-129-5p
2	miRNA	1	hsa-miR-29a
2	miRNA	1	ebv-miR-BART8
2	miRNA	1	hsa-miR-517c_hsa-miR-519a
2	miRNA	1	hsa-miR-433
2	miRNA	1	hsa-miR-432
2	DNA methylation	1	cg12845808
2	DNA methylation	1	cg04983977
2	DNA methylation	1	cg15780361
2	DNA methylation	1	cg11052143
2	DNA methylation	1	cg22442730
2	DNA methylation	1	cg16003238
2	DNA methylation	1	cg00095526
2	DNA methylation	1	cg12864235
3	mRNA	1	ENSG00000111181.8

3	mRNA	1	ENSG00000196517.7
3	mRNA	1	ENSG00000011426.6
3	mRNA	1	ENSG00000198624.8
3	mRNA	1	ENSG00000196154.7
3	miRNA	1	hsa-miR-132
3	miRNA	1	hsa-miR-129-5p
3	miRNA	1	hsa-miR-433
3	miRNA	1	hsa-miR-29a
3	miRNA	1	hsa-miR-432
3	DNA methylation	1	cg12845808
3	DNA methylation	1	cg22442730
3	DNA methylation	1	cg02489552
3	DNA methylation	1	cg11761535
3	DNA methylation	1	cg04983977
3	DNA methylation	1	cg03670238
4	mRNA	1	ENSG00000196517.7
4	mRNA	1	ENSG00000105643.3
4	mRNA	1	ENSG00000021300.7
4	mRNA	1	ENSG00000111181.8
4	mRNA	1	ENSG00000159176.9
4	mRNA	1	ENSG00000196154.7
4	mRNA	1	ENSG00000011426.6
4	mRNA	1	ENSG00000125534.4
4	mRNA	1	ENSG00000166508.13
4	mRNA	1	ENSG00000170759.10
4	mRNA	1	ENSG00000198624.8
4	mRNA	1	ENSG00000088836.7
4	mRNA	1	ENSG00000171388.9
4	mRNA	1	ENSG00000187984.7
4	miRNA	1	hsa-miR-132
4	miRNA	1	hsa-miR-129-5p
4	miRNA	1	hsa-miR-433
4	miRNA	1	hsa-miR-362-3p
4	miRNA	1	hsa-miR-29a
4	miRNA	1	hsa-miR-432
4	miRNA	1	ebv-miR-BART8
4	DNA methylation	1	cg12845808
4	DNA methylation	1	cg01869233
4	DNA methylation	1	cg24670715
4	DNA methylation	1	cg11052143
4	DNA methylation	1	cg04983977
4	DNA methylation	1	cg16003238
4	DNA methylation	1	cg10923408
4	DNA methylation	1	cg15780361

	5	mRNA	1	ENSG00000111181.8
	5	mRNA	1	ENSG00000105643.3
	5	mRNA	1	ENSG00000011426.6
	5	mRNA	1	ENSG00000166508.13
	5	mRNA	1	ENSG00000125534.4
	5	mRNA	1	ENSG00000188783.5
	5	mRNA	1	ENSG00000170759.10
	5	mRNA	1	ENSG00000157833.7
	5	mRNA	1	ENSG00000154945.6
	5	mRNA	1	ENSG00000153048.6
	5	mRNA	1	ENSG00000021300.7
	5	mRNA	1	ENSG00000198624.8
	5	miRNA	1	hsa-miR-132
	5	miRNA	1	hsa-miR-129-5p
	5	miRNA	1	hsa-miR-433
	5	miRNA	1	hsa-miR-133b
	5	miRNA	1	hsa-miR-29a
	5	miRNA	1	ebv-miR-BART8
	5	miRNA	1	hsa-miR-517c_hsa-miR-519a
	5	miRNA	1	hsa-miR-431
	5	miRNA	1	hsa-miR-25
	5	miRNA	1	hsa-miR-432
	5	miRNA	1	hsa-miR-95
	5	miRNA	1	hsa-miR-129-3p
	5	miRNA	1	hsa-miR-885-5p
	5	miRNA	1	hsa-miR-100
	5	miRNA	1	hsa-miR-362-3p
	5	miRNA	1	hsa-miR-1260
	5	miRNA	1	hsa-miR-99b
	5	miRNA	1	hsa-miR-200a
	5	miRNA	1	hsa-miR-504
	5	miRNA	1	hsa-miR-193b
	5	DNA methylation	1	cg12845808
	5	DNA methylation	1	cg02489552
	5	DNA methylation	1	cg15780361
	5	DNA methylation	1	cg11052143
	5	DNA methylation	1	cg06690548
SCZ	1	Protein	1	P33151
	1	Protein	1	Q15166
	1	Protein	1	P22792
	1	Protein	1	Q15063
	1	Protein	1	P06312
	1	Protein	1	H7C013
	1	Protein	1	P19823

1	Protein	1	P02743
1	Protein	1	Q14624
1	Protein	1	P13671
1	Protein	1	P01860
1	Protein	1	B7ZKJ8
1	Protein	1	Q16610
1	Protein	1	P23142
1	Protein	1	O95445
1	Protein	1	Q13790
1	Protein	1	B1AHL2
1	Protein	1	P00739
1	Metabolite	1	1263
1	Metabolite	1	400
1	Metabolite	1	704
1	Metabolite	1	351
1	Metabolite	1	541
2	Protein	1	P00739
2	Protein	1	H0Y300
2	Protein	1	P00738
2	Protein	1	Q15166
2	Protein	1	P02763
2	Protein	1	P33151
2	Protein	1	P02743
2	Protein	1	Q15063
2	Protein	1	P25774
2	Protein	1	P06312
2	Protein	1	P00390
2	Protein	1	B4E1Z4
2	Protein	1	P24593
2	Protein	1	P43121
2	Protein	1	E7ETH0
2	Protein	1	H7C013
2	Protein	1	P22792
2	Protein	1	Q13790
2	Metabolite	1	400
2	Metabolite	1	712
2	Metabolite	1	775
2	Metabolite	1	538
2	Metabolite	1	1297
2	Metabolite	1	351
2	Metabolite	1	508
3	Protein	1	P33151
3	Protein	1	Q15166
3	Protein	1	P00739

3	Protein	1	H0Y300
3	Protein	1	P00738
3	Protein	1	Q15063
3	Protein	1	P02763
3	Protein	1	P02743
3	Protein	1	P22792
3	Protein	1	P02748
3	Protein	1	P25311
3	Protein	1	P27487
3	Protein	1	P19823
3	Protein	1	Q13790
3	Protein	1	Q14624
3	Protein	1	B1AHL2
3	Protein	1	P17301
3	Protein	1	Q13103
3	Protein	1	B7ZKJ8
3	Protein	1	H7C013
3	Protein	1	P23142
3	Protein	1	P00390
3	Protein	1	P04004
3	Protein	1	P06312
3	Protein	1	P01011
3	Protein	1	Q9BXR6
3	Protein	1	B4E1Z4
3	Protein	1	H7C0L5
3	Protein	1	O95445
3	Protein	1	P43251
3	Metabolite	1	657
3	Metabolite	1	1263
3	Metabolite	1	1039
3	Metabolite	1	351
3	Metabolite	1	400
4	Protein	1	Q15166
4	Protein	1	P43121
4	Protein	1	P33151
4	Protein	1	P06312
4	Protein	1	P00739
4	Protein	1	B4E1Z4
4	Protein	1	P19823
4	Protein	1	P22792
4	Protein	1	Q13790
4	Protein	1	H7C013
4	Protein	1	P02743
4	Protein	1	P00738

4	Protein	1	H0Y300
4	Protein	1	P02763
4	Protein	1	P24593
4	Protein	1	P00390
4	Protein	1	Q15063
4	Protein	1	P25311
4	Protein	1	P02748
4	Protein	1	E7ETH0
4	Protein	1	P01011
4	Protein	1	A0A087WWT3
4	Protein	1	P25774
4	Protein	1	Q16610
4	Protein	1	P27169
4	Protein	1	P01031
4	Protein	1	P35858
4	Protein	1	A0A5F9ZHM4
4	Protein	1	Q03591
4	Protein	1	B1AHL2
4	Metabolite	1	400
4	Metabolite	1	1254
4	Metabolite	1	1263
4	Metabolite	1	538
4	Metabolite	1	351
4	Metabolite	1	992
4	Metabolite	1	1474
4	Metabolite	1	156
4	Metabolite	1	182
4	Metabolite	1	1104
4	Metabolite	1	262
4	Metabolite	1	1259
5	Protein	1	Q15166
5	Protein	1	P22792
5	Protein	1	P00739
5	Protein	1	P33151
5	Protein	1	H7C013
5	Protein	1	H0Y300
5	Protein	1	P00738
5	Protein	1	Q15063
5	Protein	1	A0A087WWT3
5	Metabolite	1	400
5	Metabolite	1	657
5	Metabolite	1	1263
5	Metabolite	1	1039
5	Metabolite	1	351

Note: Features selected by DIABLO are reported for each dataset, repeat, omics block, and latent component. These features were used for comparison with MCOF-derived biomarkers.

Table S7 Training, validation, and comparator settings for MCOF and benchmark models

A. MCOF training and validation

Item	Setting/value
Outer evaluation	Five fixed repeated stratified 80:20 holdouts; identical split files were used across all compared models and revision sensitivity analyses.
Inner validation	Five-fold stratified cross-validation within each outer training set.
Training-only preprocessing	Median imputation and standard scaling were fitted within training data and applied to validation/test data without refitting. Revision feature-budget selection used training-only f_classif within the fixed candidate panels.
Epoch selection and final fit	Early stopping with patience 30; the monitored metric was selected automatically by task type. The median best epoch across inner folds determined the final refit duration on the complete outer training set.
Architecture	Hidden dimension 128; 64 convolutional channels; kernel size 5; dropout 0.30.
Optimization	Batch size 64; learning rate 0.001; weight decay 0.0001; maximum 300 epochs; gradient clipping norm 5.0.
Reproducibility	Base random seed 1; Python 3.9.23; PyTorch 2.8.0+cu128; CUDA 12.8; NVIDIA A800 80GB PCIe.
Scope of feature-budget analysis	Selection was nested within the fixed preselected 200/1000-feature candidate panels; it does not retroactively nest the upstream construction of those panels.

B. Comparator settings

Item	Setting/value
KNN	k-nearest-neighbor classifier with 10 neighbors, evaluated using the same fixed outer splits.
SVM	RBF kernel; C=1; gamma="scale"; probability estimation enabled.
Gaussian naive Bayes	scikit-learn GaussianNB default settings.
Random forest	100 trees with the repeat-specific random seed.
DIABLO	Supervised multi-omics model with five-fold tuning within each outer training set; the same outer splits as MCOF.
MOGONET	500 pretraining and 2,500 joint-training epochs; learning rates 0.001/0.0005/0.001 for encoder pretraining, encoder updating, and VCDN; the same outer splits as MCOF.

Note: Settings were taken from the executed task manifests and validation scripts. All models and sensitivity analyses used the same five outer split assignments. Imputation, scaling, and feature-budget selection were fitted using training data only. The feature-budget analyses remain conditional on the fixed candidate panels and do not reconstruct their upstream generation.

Table S8 Sensitivity of MCOF performance to omics composition and exclusion of PAM50-overlapping genes from the BRCA RNA panel

Panel A. BRCA

Variant	ACC	Macro-F1	Weighted-F1	Macro-AUC	Weighted-AUC	Δ Macro-F1	W/T/L	Exact p	Holm p
Full multi-omics MCOF	0.900 ± 0.022	0.877 ± 0.033	0.899 ± 0.021	0.982 ± 0.003	0.979 ± 0.004	Reference	—	—	—
RNA only	0.900 ± 0.017	0.885 ± 0.027	0.901 ± 0.017	0.983 ± 0.001	0.981 ± 0.002	+0.009	3/0/2	0.5625	1.0000
miRNA only	0.817 ± 0.025	0.757 ± 0.034	0.814 ± 0.025	0.934 ± 0.017	0.939 ± 0.009	-0.120	5/0/0	0.0625	0.6250
Without 28 PAM50-overlapping RNA genes	0.884 ± 0.015	0.857 ± 0.026	0.884 ± 0.015	0.978 ± 0.004	0.976 ± 0.004	-0.019	4/0/1	0.1250	0.3750

Panel B. STAD

Variant	ACC	Macro-F1	Weighted-F1	Macro-AUC	Weighted-AUC	Δ Macro-F1	W/T/L	Exact p	Holm p
Full multi-omics MCOF	0.805 ± 0.030	0.786 ± 0.035	0.806 ± 0.028	0.934 ± 0.020	0.930 ± 0.016	Reference	—	—	—
RNA only	0.823 ± 0.059	0.810 ± 0.060	0.824 ± 0.058	0.914 ± 0.029	0.910 ± 0.029	+0.024	2/0/3	0.4375	1.0000
miRNA only	0.691 ± 0.095	0.661 ± 0.103	0.694 ± 0.091	0.836 ± 0.057	0.824 ± 0.061	-0.125	5/0/0	0.0625	0.6250

Panel C. ROSMAP

Variant	ACC	F1	AUC	MCC	Δ MCC	W/T/L	Exact p	Holm p
Full multi-omics MCOF	0.749 ± 0.040	0.751 ± 0.037	0.836 ± 0.032	0.504 ± 0.087	Reference	—	—	—
mRNA only	0.727 ± 0.027	0.742 ± 0.025	0.806 ± 0.043	0.455 ± 0.053	-0.049	3/0/2	0.3750	1.0000
miRNA only	0.631 ± 0.068	0.642 ± 0.071	0.699 ± 0.104	0.262 ± 0.135	-0.242	5/0/0	0.0625	0.7500
DNA methylation only	0.752 ± 0.044	0.765 ± 0.040	0.817 ± 0.056	0.504 ± 0.088	+0.000	2/0/3	1.0000	1.0000
Without mRNA	0.752 ± 0.026	0.760 ± 0.035	0.835 ± 0.022	0.507 ± 0.048	+0.003	2/0/3	0.9375	1.0000
Without miRNA	0.752 ± 0.051	0.766 ± 0.036	0.834 ± 0.041	0.507 ± 0.107	+0.003	2/0/3	0.9375	1.0000
Without DNA methylation	0.679 ± 0.040	0.699 ± 0.048	0.736 ± 0.050	0.357 ± 0.081	-0.147	5/0/0	0.0625	0.7500

Panel D. SCZ

Variant	ACC	F1	AUC	MCC	Δ MCC	W/T/L	Exact p	Holm p
Full multi-omics MCOF	0.819 ± 0.040	0.814 ± 0.048	0.944 ± 0.040	0.655 ± 0.078	Reference	—	—	—
Protein only	0.771 ± 0.109	0.770 ± 0.130	0.878 ± 0.107	0.574 ± 0.213	-0.082	2/0/3	0.6875	1.0000
Metabolite only	0.800 ± 0.085	0.810 ± 0.072	0.889 ± 0.088	0.602 ± 0.174	-0.054	3/1/1	0.3750	1.0000

Note: Values are mean ± SD across five repeated stratified holdouts. Δ is the variant minus the full multi-omics MCOF for the prespecified primary metric (macro-F1 for BRCA/STAD and MCC for ROSMAP/SCZ); positive values therefore favor the variant. W/T/L gives the numbers of repeats in which full MCOF outperformed, tied, or underperformed the variant. Exact two-sided sign-flip tests and Holm-adjusted p values are exploratory because only five matched repeats were available and held-out samples may overlap across repeats. The BRCA sensitivity analysis removed the 28 PAM50 genes present in the 1,000-feature RNA panel, leaving 972 RNA features.

Table S9 Sensitivity of MCOF performance to training-set-only feature budgets within the fixed candidate panels

Panel A. BRCA

Variant	ACC	Macro-F1	Weighted-F1	Macro-AUC	Weighted-AUC	Δ Macro-F1	W/T/L	Exact p	Holm p
Full multi-omics MCOF	0.900 ± 0.022	0.877 ± 0.033	0.899 ± 0.021	0.982 ± 0.003	0.979 ± 0.004	Reference	—	—	—
Training-only k=100 per omics	0.880 ± 0.021	0.867 ± 0.025	0.882 ± 0.020	0.977 ± 0.007	0.973 ± 0.007	-0.010	3/0/2	0.6250	1.0000
Training-only k=250 per omics	0.877 ± 0.018	0.852 ± 0.030	0.876 ± 0.022	0.981 ± 0.004	0.978 ± 0.004	-0.024	3/0/2	0.2500	1.0000
Training-only k=500 per omics	0.883 ± 0.010	0.850 ± 0.020	0.881 ± 0.010	0.979 ± 0.002	0.977 ± 0.003	-0.026	4/0/1	0.1875	1.0000

Panel B. STAD

Variant	ACC	Macro-F1	Weighted-F1	Macro-AUC	Weighted-AUC	Δ Macro-F1	W/T/L	Exact p	Holm p
Full multi-omics MCOF	0.805 ± 0.030	0.786 ± 0.035	0.806 ± 0.028	0.934 ± 0.020	0.930 ± 0.016	Reference	—	—	—
Training-only k=100 per omics	0.823 ± 0.049	0.806 ± 0.056	0.826 ± 0.046	0.952 ± 0.020	0.951 ± 0.017	+0.020	2/0/3	0.4375	1.0000
Training-only k=250 per omics	0.814 ± 0.052	0.803 ± 0.059	0.816 ± 0.050	0.939 ± 0.026	0.930 ± 0.028	+0.017	1/0/4	0.2500	1.0000
Training-only k=500 per omics	0.809 ± 0.073	0.784 ± 0.089	0.810 ± 0.072	0.929 ± 0.030	0.927 ± 0.025	-0.002	2/0/3	0.9375	1.0000

Panel C. ROSMAP

Variant	ACC	F1	AUC	MCC	Δ MCC	W/T/L	Exact p	Holm p
Full multi-omics MCOF	0.749 ± 0.040	0.751 ± 0.037	0.836 ± 0.032	0.504 ± 0.087	Reference	—	—	—
Training-only k=50 per omics	0.704 ± 0.028	0.704 ± 0.028	0.766 ± 0.024	0.422 ± 0.067	-0.082	4/0/1	0.1875	1.0000
Training-only k=100 per omics	0.744 ± 0.044	0.751 ± 0.046	0.812 ± 0.033	0.491 ± 0.087	-0.013	3/0/2	0.8125	1.0000

Panel D. SCZ

Variant	ACC	F1	AUC	MCC	Δ MCC	W/T/L	Exact p	Holm p
Full multi-omics MCOF	0.819 ± 0.040	0.814 ± 0.048	0.944 ± 0.040	0.655 ± 0.078	Reference	—	—	—

Variant	ACC	F1	AUC	MCC	Δ MCC	W/T/L	Exact p	Holm p
Training-only k=50 per omics	0.790 ± 0.099	0.777 ± 0.125	0.880 ± 0.087	0.606 ± 0.186	-0.050	3/0/2	0.6875	1.0000
Training-only k=100 per omics	0.800 ± 0.179	0.789 ± 0.212	0.880 ± 0.144	0.604 ± 0.352	-0.051	2/0/3	0.9375	1.0000

Note: For each inner-training fold and for the final fit on the complete outer training set, ANOVA F-based feature selection (f_classif) was fitted using training samples only and then applied without refitting. k is the number of features retained per omics layer. These analyses evaluate feature-budget sensitivity within the fixed candidate panels and do not reconstruct the upstream panel-generation procedure. Δ and W/T/L are defined as in Table S8.

Table S10 Class-specific held-out performance of MCOF on the restricted BRCA and STAD subtype tasks

Panel A. BRCA

Class	Test n by repeat	Sensitivity/recall	Specificity	Precision	F1	One-vs-rest AUC
Basal-like	36;36;36;36;36	1.000 ± 0.000	0.992 ± 0.007	0.963 ± 0.029	0.981 ± 0.015	1.000 ± 0.000
HER2-enriched	16;16;16;16;16	0.863 ± 0.135	0.983 ± 0.005	0.810 ± 0.047	0.832 ± 0.086	0.991 ± 0.005
Luminal A	110;110;110;110;110	0.920 ± 0.032	0.932 ± 0.028	0.943 ± 0.022	0.931 ± 0.014	0.978 ± 0.004
Luminal B	39;39;39;39;39	0.764 ± 0.090	0.942 ± 0.029	0.771 ± 0.087	0.762 ± 0.046	0.958 ± 0.008

Panel B. STAD

Class	Test n by repeat	Sensitivity/recall	Specificity	Precision	F1	One-vs-rest AUC
CIN	25;25;25;25;25	0.816 ± 0.067	0.853 ± 0.101	0.886 ± 0.059	0.846 ± 0.019	0.925 ± 0.022
GS	10;10;10;10;10	0.720 ± 0.130	0.906 ± 0.053	0.712 ± 0.110	0.704 ± 0.077	0.904 ± 0.053
MSI	9;9;9;9;9	0.867 ± 0.050	0.926 ± 0.048	0.764 ± 0.121	0.808 ± 0.079	0.975 ± 0.016

Note: Values are mean ± SD across five repeated stratified holdouts. Test n by repeat lists the class-specific sample counts in the five held-out test sets. Sensitivity/recall, specificity, precision, F1, and AUC were calculated one-versus-rest for each class. The BRCA task included Basal-like, HER2-enriched, Luminal A, and Luminal B; the STAD task included CIN, GS, and MSI. These results should not be generalized to normal-like BRCA or EBV-positive STAD.

Table S11 Implementation and numerical robustness checks for MCOF

Panel A. Batch-independence verification for the 20 formal MCOF checkpoints

Scope	Tested	Passed	Batch n	Max |Δ logit|: alone vs batch	Max |Δ logit|: original vs reversed batch	Max gradient on other samples	Tolerance
All four datasets	20	20	8	2.193 × 10−5	0	0	1 × 10−4

Note: Each checkpoint was evaluated with a batch of eight samples. Predictions were compared when each sample was evaluated alone, in its original batch, and after reversing batch order; the selected logit for sample 0 was also differentiated with respect to every batch row. All 20 checkpoints passed the prespecified 1 × 10−4 tolerance. The maximum batch-order difference and maximum cross-sample gradient were both zero, and the maximum alone-versus-batch difference was 2.193 × 10−5. Checkpoint-level results are provided in Supplementary Data S2.

Panel B. Numerical convergence of integrated-gradients rankings

Dataset	Steps compared	Spearman mean ± SD	Top-100 Jaccard mean ± SD	Relative L1 change mean ± SD	Mean |completeness Δ| at later step	Max |completeness Δ| at later step
BRCA	25 vs 50	0.989 ± 0.010	0.819 ± 0.128	0.054 ± 0.030	1.692	36.412
BRCA	50 vs 100	0.998 ± 0.002	0.935 ± 0.053	0.021 ± 0.011	0.416	7.805
ROSMAP	25 vs 50	0.998 ± 0.003	0.969 ± 0.026	0.015 ± 0.009	0.083	0.563
ROSMAP	50 vs 100	1.000 ± 0.000	0.992 ± 0.011	0.002 ± 0.002	0.013	0.129
SCZ	25 vs 50	0.999 ± 0.000	0.942 ± 0.023	0.016 ± 0.005	0.074	0.703
SCZ	50 vs 100	1.000 ± 0.000	0.992 ± 0.011	0.002 ± 0.000	0.011	0.063
STAD	25 vs 50	0.999 ± 0.000	0.931 ± 0.017	0.017 ± 0.004	0.293	9.219
STAD	50 vs 100	1.000 ± 0.000	0.988 ± 0.011	0.003 ± 0.001	0.064	1.527

Note: Convergence was assessed by deterministic Gauss–Legendre quadrature using the first eight held-out samples per observed class in each repeat, with every output class evaluated. The all-zero vector in the checkpoint-specific training-standardized feature space served as the baseline. Between 50 and 100 steps, the mean Spearman rank correlation was at least 0.998 and the mean Top-100 Jaccard similarity was at least 0.935 in every dataset. Completeness deltas decreased at 100 steps in every dataset. Completeness values are reported on the model-logit scale and should be interpreted together with rank concordance and relative L1 change. Class-aggregation sensitivity and chance-adjusted overlap are reported in revised Table S5 and Supplementary Data S1.
